# The role and mechanism of tubulin folding cofactor B in glioblastoma migration

**DOI:** 10.1038/s41420-026-03232-6

**Published:** 2026-06-30

**Authors:** Bingqiu Zhang, Qiang Wang, Chenglong Ouyang, Mei Yang, Qiuxi Chen, Xiaoqiao Chen, Fei Gao, Hui Liu

**Affiliations:** 1 Experimental Teaching Management Center, Chongqing Medical University, Chongqing, China; 2 Institute of Neuroscience, Chongqing Medical University, Chongqing, China; 3 The First Affiliated Hospital of Chongqing Medical University, Chongqing, China

**Keywords:** Microtubules, CNS cancer, Cell invasion, Astrocyte

## Abstract

Glioblastoma (GBM) is the most common primary malignant brain tumor in the central nervous system, characterized by high invasiveness, incidence, and mortality. Tubulin folding cofactor B (TBCB) is a microtubule-associated regulator implicated in microtubule plus-end organization and related structural features; however, its role and underlying mechanisms in GBM remain unclear. This study aimed to elucidate the function and mechanisms of TBCB in GBM cell proliferation and migration. Analyses of public databases (GEO, TCGA, CGGA) and clinical GBM specimens revealed that TBCB was significantly upregulated in GBM compared with normal controls, with higher expression correlating with higher WHO grades and poor prognosis. Functionally, TBCB knockdown in U87 and U251 cells significantly inhibited proliferation and migration (assessed by EdU, cell counting, Transwell and wound-healing assays) and impaired cell process formation; consistently, in vivo experiments using orthotopic intracranial xenograft models confirmed that TBCB knockdown suppressed tumor growth and prolonged survival. Mechanistically, reciprocal Co-IP/IF supported association of TBCB with both EB1 and EB3, indicating that TBCB acts as a functional partner of the microtubule plus-end tracking proteins ( + TIPs) EB1 and EB3, co-localizing at cell process tips and being associated with microtubule plus-end organization/comet-like features. Furthermore, Kyoto Encyclopedia of Genes and Genomes (KEGG) pathway analysis identified the MAPK pathway as a key signaling pathway associated with TBCB; Western blotting showed that TBCB knockdown reduced the p-ERK/ERK ratio, and pharmacological modulation with the ERK agonist TPA and the MEK inhibitor U0126 further indicated that ERK-pathway perturbation regulates TBCB in a time-dependent and nonlinear manner in GBM cells. Collectively, these findings indicate that TBCB promotes GBM cell proliferation and migration through EB1/EB3-associated microtubule plus-end organization and is functionally coupled to the ERK–MAPK pathway in a time-dependent and nonlinear manner, suggesting that TBCB is a candidate regulator of GBM progression that warrants further translational validation.

## Introduction

According to the World Health Organization (WHO) classification of malignant tumors, gliomas, which are the most prevalent primary malignant tumors in the central nervous system in adults, are graded from I to IV [1]. Glioblastoma (GBM), as grade IV, represents the most common and aggressive form of glioma. The median survival time for patients diagnosed with GBM is only 12–15 months [2]. GBM is characterized by infiltrative growth into the surrounding brain parenchyma, often leading to tumor recurrence and neurological dysfunction, posing a serious threat to patients’ lives [3]. Consequently, elucidating the molecular mechanisms that drive GBM growth, migration, and proliferation remains a major focus in neuro-oncology [4].

Tubulin folding cofactor B (TBCB), a member of the tubulin-folding cofactor family (TBCA–TBCE) [5], is a key regulator of microtubule assembly [6], growth and dynamic stability [7]. TBCB is highly expressed in the neuronal growth cone transition zone during neurogenesis [8], co-localizes with microtubules at the edge of early oocytes and accumulates at the distal ends of growing neural processes [9], implicating it in the control of microtubule plus-end growth. In tumor cells, TBCB overexpression promotes microtubule depolymerization [10] and is closely linked to proliferation, migration and invasion [11, 12]. Analysis of public glioma datasets together with our own clinical specimens further suggests that TBCB is upregulated in gliomas, increases with WHO grade and correlates with poor prognosis, indicating a potential role in glioma initiation and progression.

Microtubule plus-ends are crucial for tumor cell proliferation, invasion and migration [13]. However, TBCB does not bind microtubules directly [14]. Most proteins that regulate the microtubule plus-end act through end-binding proteins (EBs) [15]. EBs (EB1, EB2 and EB3) constitute a family of microtubule plus-end tracking proteins [16, 17]; among them, EB1 and EB3 accumulate at the distal ends of growing microtubules in neurons and promote persistent growth [18, 19]. In GBM, high EB1 expression is associated with increased mitosis and migration [20]. The N-terminal calponin-homology (CH) domain of EB1 mediates microtubule binding [21], whereas the C-terminal EEY/F motif interacts with CAP-Gly–containing proteins [22]. Because TBCB contains a C-terminal CAP-Gly domain [14], and EB1/EB3 harbor C-terminal motifs known to engage CAP-Gly-containing partners, TBCB and EB proteins may associate at microtubule plus-end-associated regions; however, the recruitment order and functional directionality of this association remain unresolved.

Transcriptomic analyses of TBCB-silenced glioma cells further pointed to mitogen-activated protein kinase (MAPK) signaling as a prominently enriched pathway. MAPKs, including ERK, p38 and JNK, are key regulators of cytoskeletal stability, cell migration, proliferation and differentiation [23–26]. The ERK pathway is frequently dysregulated in human cancers [27], aberrantly activated in gliomas [28] and promotes tumor progression [29, 30]. Previous work also indicates that ERK signaling modulates TBCB expression in astrocytes [31], and can directly regulate microtubule organization by phosphorylating microtubule-associated proteins. These observations suggest that the MAPK/ERK pathway is a strong candidate upstream regulator of TBCB and its cytoskeletal functions in glioma.

Taken together, these considerations led us to hypothesize that TBCB contributes to GBM progression in association with EB1/EB3-linked microtubule plus-end organization and is functionally coupled to MAPK/ERK signaling. In this study, we combined analyses of public datasets and clinical tissues with in vitro and in vivo functional experiments to: (i) characterize TBCB expression and its association with clinicopathological features in glioma; (ii) define the impact of TBCB on GBM cell proliferation, migration and process formation; (iii) characterize the association and functional coupling between TBCB and the +TIPs EB1 and EB3 at microtubule plus-end-associated regions; and (iv) elucidate the role of MAPK/ERK signaling in controlling TBCB expression and its downstream phenotypes in GBM cells.

## Results

### TBCB is upregulated in GBM and is associated with poor prognosis

**Fig. 1:**
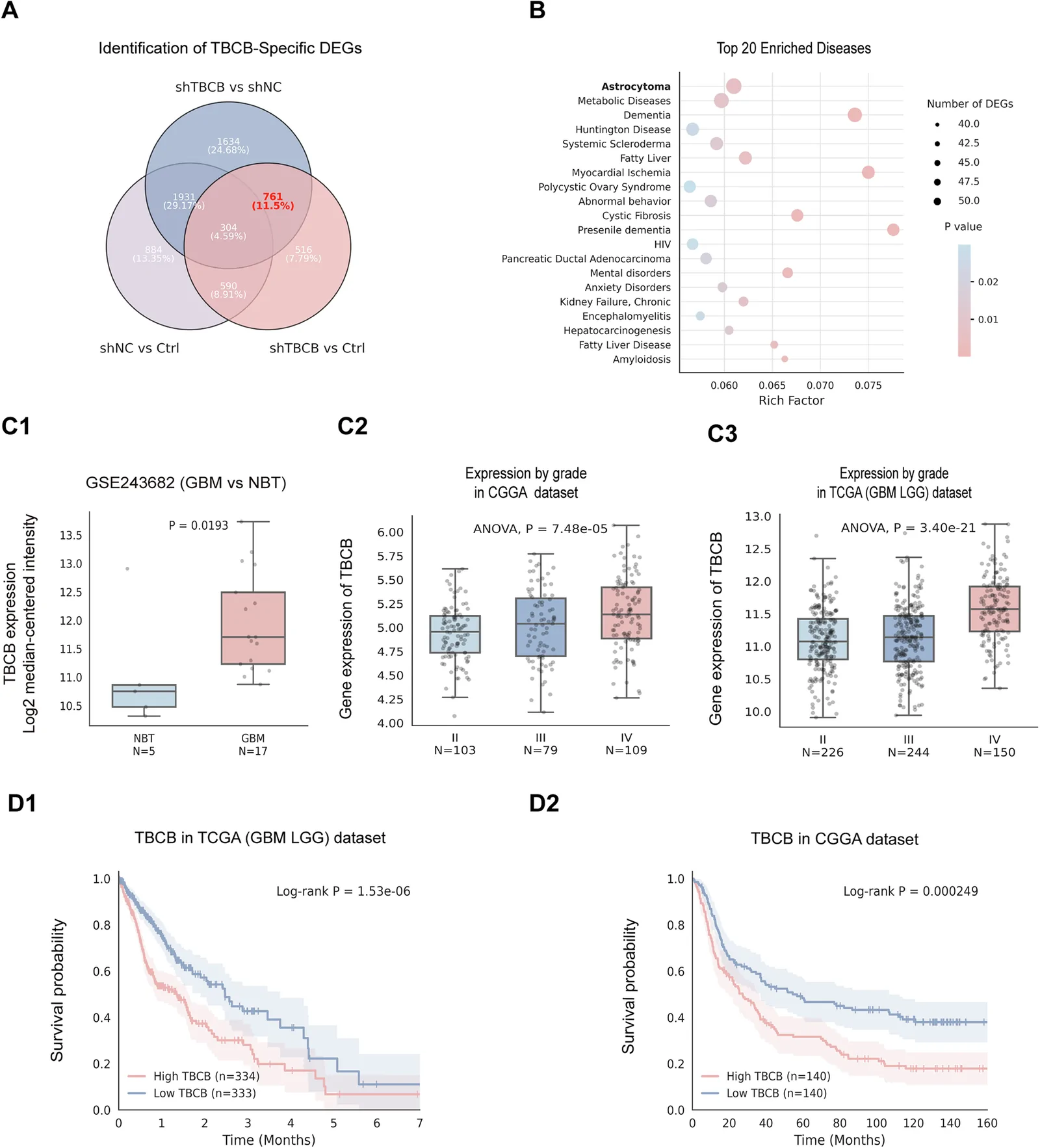
TBCB is upregulated in glioma and associated with poor prognosis. **A** Three-set Venn diagram showing the overlap among differentially expressed genes (DEGs) identified in three pairwise comparisons in HT22 cells: shTBCB vs Ctrl, shTBCB vs shNC, and shNC vs Ctrl. Ctrl untreated control cells, shNC control lentivirus-transduced cells, shTBCB TBCB-targeting lentivirus-transduced cells. The 761 TBCB-related DEGs used for downstream analysis correspond to genes shared by the shTBCB vs Ctrl and shTBCB vs shNC comparisons after exclusion of genes identified in shNC vs Ctrl; the full gene list, together with the corresponding log2 fold changes and nominal *P* values, is provided in Supplementary Table S6. The repository status of the HT22 RNA-seq dataset is provided in the Data availability statement. **B** Disease enrichment analysis of the TBCB-related DEG set using DisGeNET, displaying the top 20 enriched disease terms ranked by enrichment score; dot size indicates the number of DEGs and color encodes the *P* value. Disease terms with *P* < 0.05 were considered significantly enriched. Astrocytoma is among the top enriched categories. **C** TBCB mRNA expression in public glioma datasets: **C1** GEO GSE243682, glioblastoma (GBM) vs normal brain tissue (NBT); **C2** CGGA cohort stratified by WHO grade (II/III/IV); **C3** TCGA cohort showing TBCB expression in lower-grade glioma (LGG) vs GBM and across WHO grades II/III/IV. Group comparisons were performed using one-way ANOVA with multiple comparisons (for normally distributed data with equal variances) or Kruskal–Wallis test with Dunn’s multiple comparisons (for non-normal data). **D** Kaplan–Meier overall survival (OS) curves stratified by TBCB expression level: **D1** TCGA glioma cohort; **D2** CGGA glioma cohort. Survival differences between high- and low-TBCB expression groups were compared using two-sided log-rank tests. Data are presented as mean ± SD where applicable. *P < 0.05, **P < 0.01, ***P < 0.001.

Transcriptomic profiling was performed in HT22 cells under three conditions: untreated control (Ctrl), control lentivirus transduction (shNC), and TBCB-targeting lentiviral knockdown (shTBCB). A set of 761 TBCB-related DEGs, defined as the overlap between shTBCB vs Ctrl and shTBCB vs shNC after exclusion of genes identified in shNC vs Ctrl, was used for downstream DisGeNET enrichment analysis; the full gene list and corresponding log2 fold changes and nominal P values are provided in Supplementary Table S6, and astrocytoma emerged as the top enriched disease term (Fig. 1A, B). In the GEO dataset GSE243682, TBCB mRNA levels were significantly higher in glioblastoma (GBM) than in normal brain tissue (NBT). In the TCGA and CGGA cohorts, TBCB expression progressively increased with WHO grade and was significantly higher in GBM than in lower-grade glioma (LGG) (Fig. 1C). Kaplan–Meier analyses further showed that high TBCB expression was associated with shorter overall survival in both datasets (Fig. 1D), indicating that TBCB upregulation is linked to glioma progression and poor prognosis. The HT22 RNA-seq dataset supporting the disease-enrichment analysis has been submitted to GSA under BioProject PRJCA053575 and Submission ID subCRA057806.

**Fig. 2:**
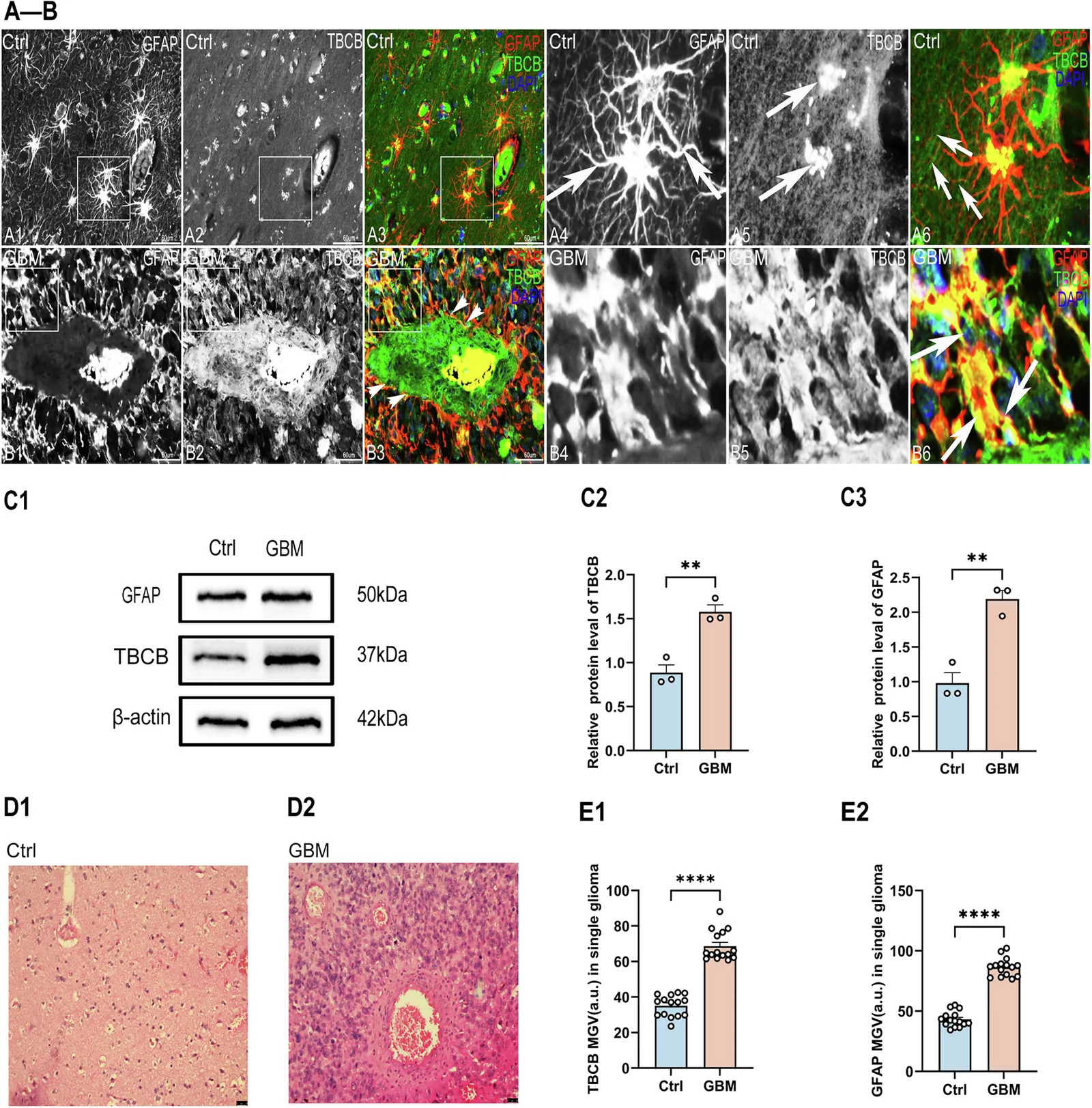
Expression of TBCB and glial fibrillary acidic protein (GFAP) in glioblastoma tissues. Immunofluorescence (IF) staining showing co-expression of GFAP (red) and TBCB (green) in cancer-adjacent control brain tissue (Ctrl, **A**) and glioblastoma (GBM) tissue (**B**). Subpanels 2–6 display grayscale images of GFAP or TBCB and their merged views; panels 4–6 are magnified views of the boxed regions in panels 2–3. Because individual cell boundaries were not consistently resolvable in tissue sections, these IF images were used for descriptive histological comparison and were not subjected to per-cell morphometric quantification. Representative IF images are shown from *n* = 3 biological specimens per group. **C1** Western blot analysis of TBCB and GFAP expression in cancer-adjacent control brain tissue (Ctrl) and GBM tissue. **C2**, **C3** Quantification of TBCB and GFAP protein levels (*n* = 3 biological samples per group). **D1**, **D2** Representative hematoxylin and eosin (H&E) staining images of cancer-adjacent control brain tissue (Ctrl) and GBM tissue. Representative H&E images are shown from *n* = 3 biological specimens per group. **E1**, **E2** Quantification of the mean grey value of TBCB and GFAP in tissue sections (*n* = 3 biological specimens per group). Data are presented as mean ± SD. *P < 0.05, **P < 0.01, ***P < 0.001, ****P < 0.0001.

We next validated these findings in clinical GBM samples. Immunofluorescence staining showed co-expression of glial fibrillary acidic protein (GFAP) and TBCB in both cancer-adjacent control brain tissue and GBM tissue (Fig. 2A, B). In control tissue, GFAP-positive astrocytes with slender processes were readily recognizable, and TBCB signal displayed a coarse granular pattern enriched near microtubule-organizing center (MTOC)-related regions. In GBM tissue, the overall cytoarchitecture was more disorganized, and although the morphology of many cells was not clearly distinguishable, identifiable astrocyte-like cells showed TBCB signal extending along their processes. Because individual cell boundaries were not consistently resolvable in tissue sections, per-cell morphometric quantification was not performed for these IF images.

Western blotting further showed that both TBCB and GFAP protein levels were elevated in GBM tissue relative to cancer-adjacent control tissue (Fig. 2C1–C3). Representative H&E staining confirmed the histopathological distinction between control and GBM tissue (Fig. 2D1, D2). Consistently, mean grey value analysis in tissue sections showed higher TBCB and GFAP signal intensity in GBM tissue than in control tissue (Fig. 2E1, E2). Because MTOC-related organization is linked to proliferative cell behavior [32, 33], these findings suggest that TBCB is associated with GBM proliferative capacity.

TBCB signal was also observed in vascular endothelial cells, astrocytic endfeet around blood vessels, and in the processes of a subset of GBM cells that retained star-like morphology (Fig. 2B). Given the importance of perivascular structures and cell processes for GBM growth and invasion, these observations raised the possibility that TBCB contributes to GBM cell migration. We therefore proceeded to functional assays in GBM cell lines and used SVG P12 cells as a non-neoplastic reference in the subsequent morphology-associated and microtubule-organization analyses.

### TBCB promotes GBM proliferation and migration and is associated with protrusive cell morphology

**Fig. 3:**
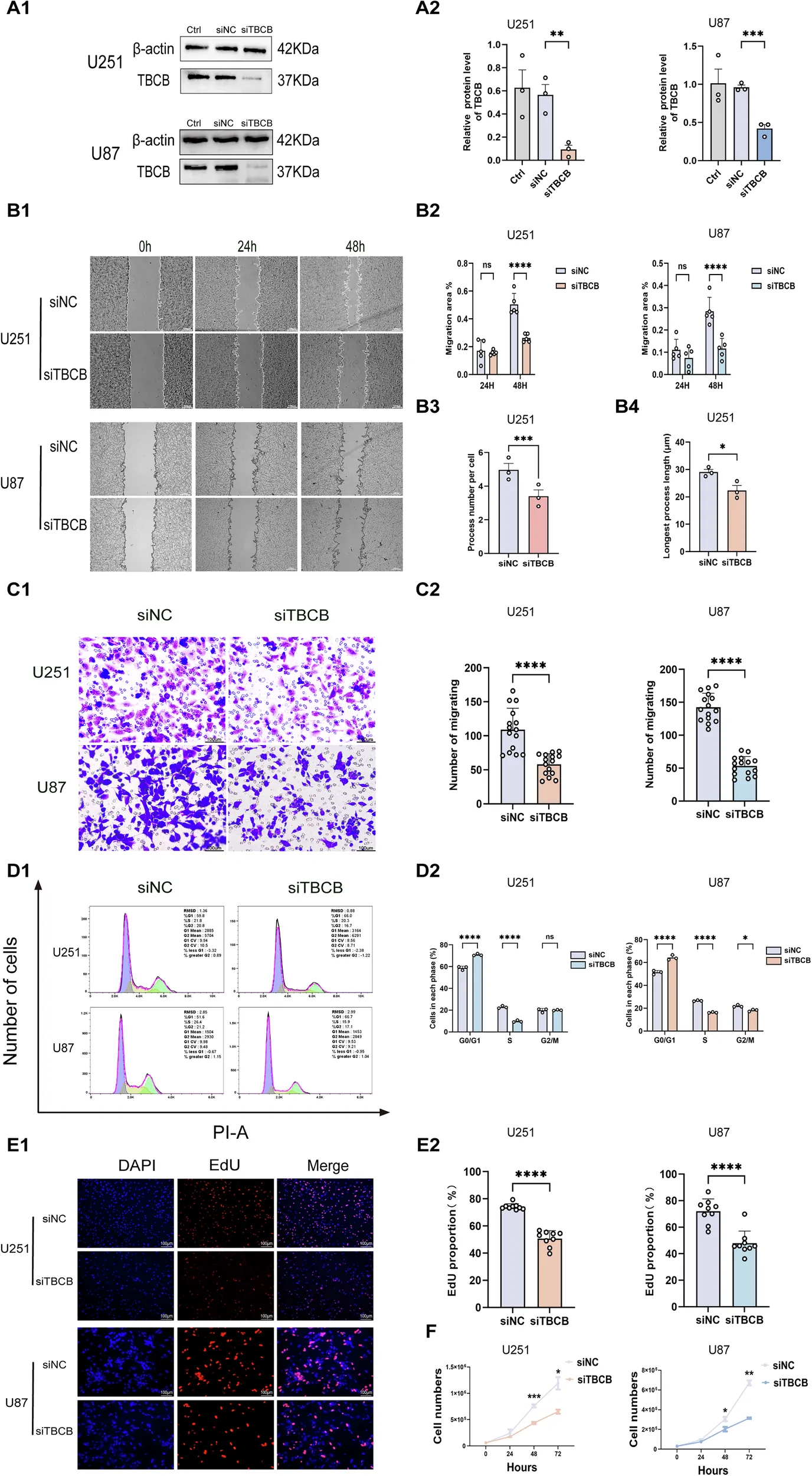
TBCB knockdown inhibits migration-associated protrusive morphology and proliferation and alters cell-cycle distribution in glioblastoma cells in vitro. **A1** Representative Western blots showing TBCB expression in U251-Ctrl, U251-siNC, U251-siTBCB, U87-Ctrl, U87-siNC, and U87-siTBCB cells. **A2** Quantification of TBCB protein levels in U251 and U87 cells. **B1** Representative wound-healing images of U251 and U87 cells at the indicated time points after scratching under low-serum conditions. **B2** Quantification of wound closure in U251 and U87 cells. **B3** Quantification of process number per cell in wound-edge U251 cells at 24 h after scratching. **B4** Quantification of longest process length per cell in wound-edge U251 cells at 24 h after scratching. **C1** Representative Transwell migration images of U251 and U87 cells. **C2** Quantification of migrated cells. **D1** Representative PI DNA-content histograms of U251 and U87 cells after TBCB knockdown. TBCB knockdown increased the G0/G1 fraction and decreased the S-phase fraction in both U251 and U87 cells, whereas the G2/M fraction was not significantly changed. **D2** Quantification of cell-cycle distribution. **E1** Representative EdU staining images of U251 and U87 cells. **E2** Quantification of the percentage of EdU-positive cells. **F** Cell-counting-based growth curves of U251 and U87 cells. For Western blotting, wound-healing, Transwell migration, PI cell-cycle, EdU, and cell-counting assays, *n* = 3 independent biological experiments unless otherwise indicated. For wound-edge morphometric analysis, at least 30 cells per group were analyzed in each independent experiment. Data are presented as mean ± SD. Comparisons between two groups were performed using two-sided Student’s t-tests. *P < 0.05, **P < 0.01, ***P < 0.001, ****P < 0.0001.

**Fig. 4:**
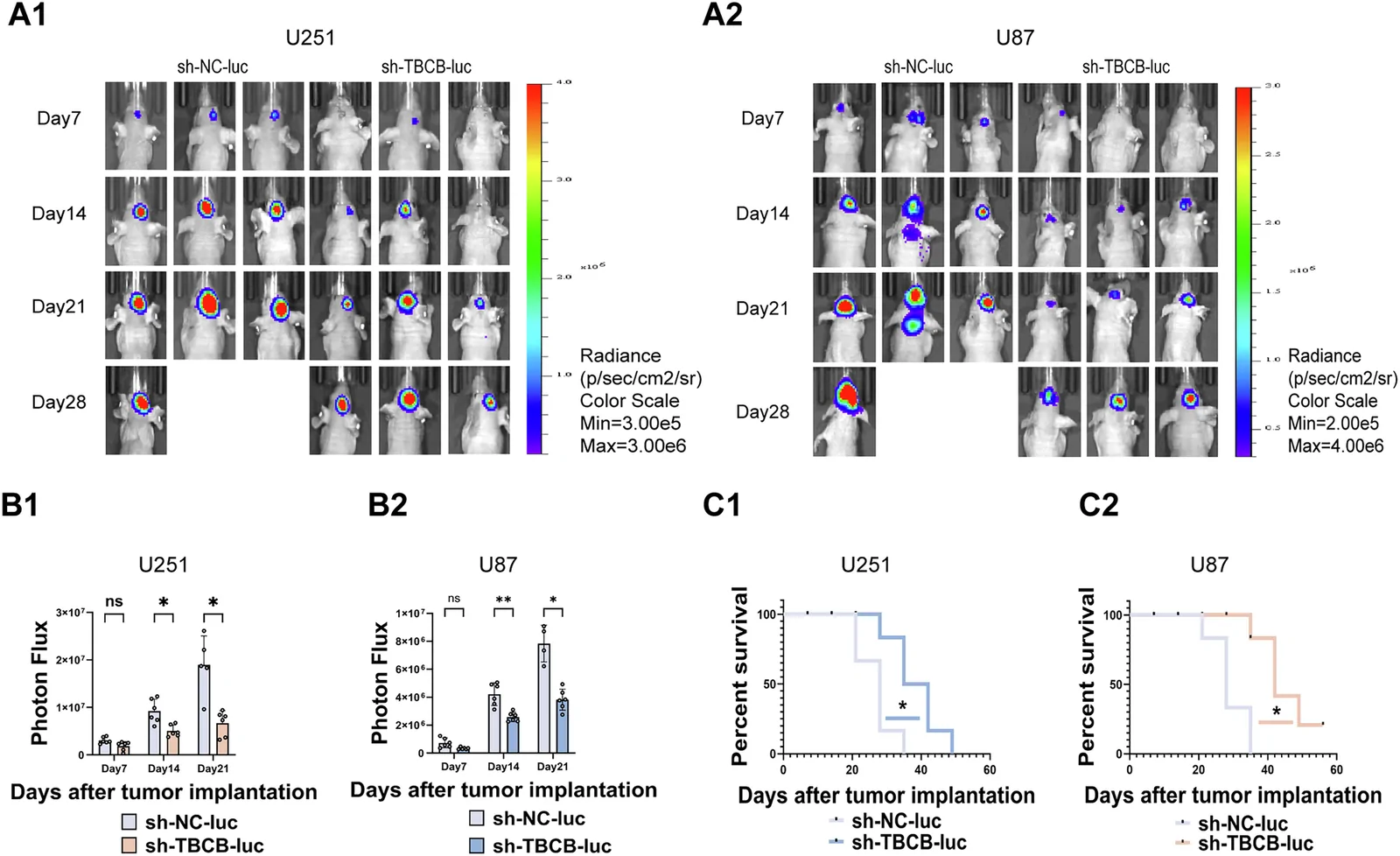
TBCB knockdown suppresses GBM xenograft growth in vivo and prolongs survival. **A** Representative IVIS images of orthotopic intracranial xenografts at the indicated time points. U251 (3 × 10^5 cells/mouse) and U87 (5 × 10^5 cells/mouse) cells stably expressing luciferase and transduced with shTBCB or control shRNA (shNC) were stereotactically injected into the brains of 6-week-old female BALB/c nude mice (*n* = 6 per group). Representative IVIS images of intracranial tumors in U251 (A1) and U87 (A2) xenografts at the indicated time points are shown. **B** Quantification of bioluminescent signal intensity (photons/s/cm²/sr) reflecting tumor burden in U251 (**B1**) and U87 (**B2**) xenografts. Data are presented as mean ± SD. **C** Kaplan–Meier survival curves of mice bearing U251 (**C1**) or U87 (**C2**) tumors expressing shNC or shTBCB. Survival differences between groups were analyzed using two-sided log-rank (Mantel–Cox) tests. *P < 0.05, **P < 0.01, ***P < 0.001 vs shNC.

To assess the functional role of TBCB, we transiently silenced TBCB in U251 and U87 cells using siRNA. Western blotting confirmed efficient knockdown of TBCB protein in both cell lines (Fig. 3A1, A2). TBCB silencing markedly impaired GBM cell migration. In wound-healing assays performed under low-serum conditions, control U251 and U87 cells closed the scratch substantially faster than siTBCB cells (Fig. 3B1, B2). Consistent with this result, Transwell assays showed significantly fewer migrated cells after TBCB knockdown in both cell lines (Fig. 3C1, C2). To convert the wound-edge morphological differences into quantitative readouts, we further analyzed U251 cells at the wound edge 24 h after scratching. Compared with U251-siNC cells, U251-siTBCB cells exhibited significantly fewer processes per cell and a shorter longest process length per cell (Fig. 3B3, B4), providing quantitative support that TBCB depletion suppresses protrusive morphology associated with GBM cell migration. PI DNA-content analysis further showed that TBCB knockdown increased the proportion of cells in G0/G1 phase and decreased the proportion in S phase in both U251 and U87 cells, whereas the G2/M fraction was not significantly altered (Fig. 3D1, D2), consistent with altered cell-cycle progression. Consistently, TBCB depletion reduced the proportion of EdU-positive cells and slowed cell growth in cell-counting assays (Fig. 3E1, E2, F), supporting a role for TBCB in GBM proliferative capacity. Together, these results indicate that TBCB promotes GBM proliferation and migration and is associated with the formation of protrusive cell morphology at the wound edge. These functional effects are summarized in Figs. 3 and 4.

We further evaluated the role of TBCB in vivo using intracranial xenograft models. U251 and U87 cells expressing luciferase and transduced with shTBCB or control shRNA were implanted into nude mouse brains. Bioluminescence imaging demonstrated consistently weaker signals in the shTBCB group than in the shNC group throughout the observation period, indicating reduced tumor growth (Fig. 4A, B). Mice bearing shTBCB tumors also showed significantly prolonged survival compared with controls (Fig. 4C). These findings support a role for TBCB in promoting GBM tumor growth in vivo.

### TBCB modulates microtubule plus-end organization in GBM cell processes

**Fig. 5:**
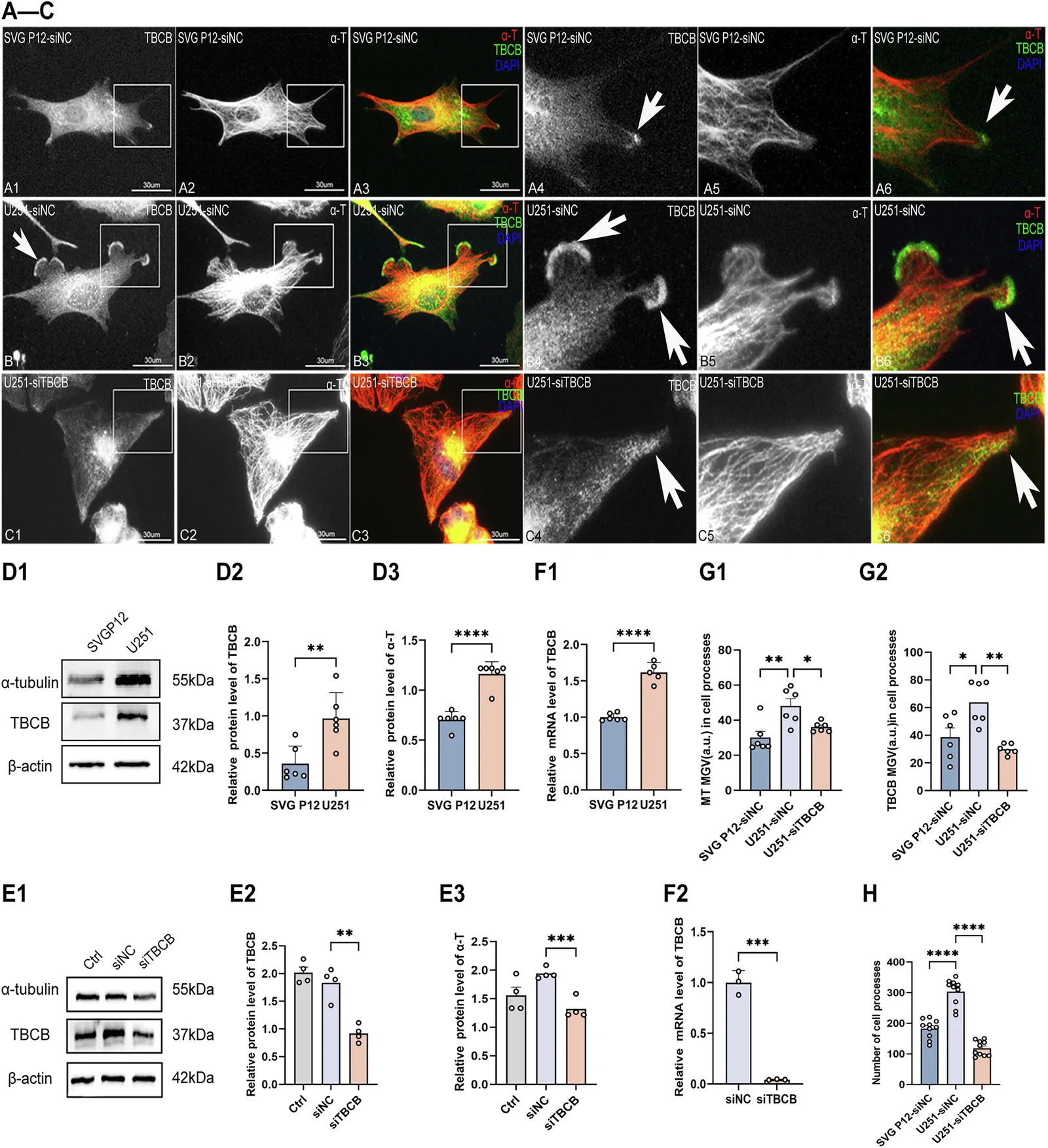
TBCB-associated microtubule organization differs between SVG P12 cells, U251-siNC cells, and TBCB-silenced U251 cells. Representative immunofluorescence (IF) images showing TBCB (green) and α-tubulin (red) in SVG P12-siNC cells (**A**), U251-siNC cells (**B**), and U251-siTBCB cells (**C**). Subpanels show grayscale views of each channel and merged images; higher-magnification views highlight microtubule organization and TBCB distribution in cell processes. **D1** Representative Western blots of α-tubulin, TBCB, and β-actin in untreated SVG P12 and U251 cells. **D2**, **D3** Quantification of TBCB and α-tubulin protein levels in untreated SVG P12 and U251 cells. **E1** Representative Western blots of α-tubulin, TBCB, and β-actin in U251-Ctrl, U251-siNC, and U251-siTBCB cells. **E2**, **E3** Quantification of TBCB and α-tubulin protein levels in U251 cells after TBCB knockdown. **F1** RT-qPCR analysis of TBCB mRNA in untreated SVG P12 and U251 cells. **F2** RT-qPCR analysis of TBCB mRNA in U251-siNC and U251-siTBCB cells. **G1**, **G2** Quantification of α-tubulin and TBCB mean grey values (MGV = integrated density/area) in cell processes. **H** Quantification of cell process number per cell. α-Tubulin serves as a structural readout of the underlying microtubule array. Western blotting, RT-qPCR, and image-based quantification were performed with *n* = 3 independent biological experiments unless otherwise indicated. Data are presented as mean ± SD. *P < 0.05, **P < 0.01, ***P < 0.001, ****P < 0.0001.

Because microtubule plus-end organization is closely linked to process formation [13], we examined the relationship between TBCB distribution and microtubule organization. α-Tubulin staining was used to visualize the overall microtubule array in cell bodies and processes. In SVG P12 cells, α-tubulin–labeled microtubules formed radial, well-organized arrays from the perinuclear region to the cell cortex, and TBCB was diffusely distributed with enrichment at sharp nascent processes (Fig. 5A). In U251 cells, processes were shorter and more numerous, and many had obtuse tips. In these cells, microtubules appeared denser and more disorganized, with interlacing bundles and curled plus-ends, and TBCB expression was markedly increased along these processes and at their tips (Fig. 5B, G).

Knockdown of TBCB in U251 cells restored a more astrocyte-like morphology, characterized by fewer, sharper processes and more orderly microtubule arrays (Fig. 5C, G, H). TBCB signal remained detectable but was reduced and redistributed toward the tips of sharp processes, resembling the pattern observed in SVG P12 cells (Fig. 5A, C). Together, these fixed-cell observations indicate that TBCB is required for the aberrant organization of microtubule plus-ends and the formation of obtuse, migratory processes in GBM cells. Consistent with the imaging findings, baseline comparison panels showed higher TBCB and α-tubulin protein levels, together with higher TBCB mRNA, in U251 cells than in SVG P12 cells (Fig. 5D1–D3, F1). Within U251 cells, TBCB knockdown reduced both TBCB-associated and α-tubulin-associated protein readouts and reduced TBCB mRNA (Fig. 5E1–E3, F2). In addition, mean grey value analysis and process-related quantification further supported the accompanying reduction in process-associated signal intensity and protrusive morphology after TBCB depletion (Fig. 5G1, G2, H). These data support a role for TBCB in process-associated microtubule organization rather than directly demonstrating microtubule dynamic parameters.

### EB1 and EB3 are plus-end binding partners in GBM cells

**Fig. 6:**
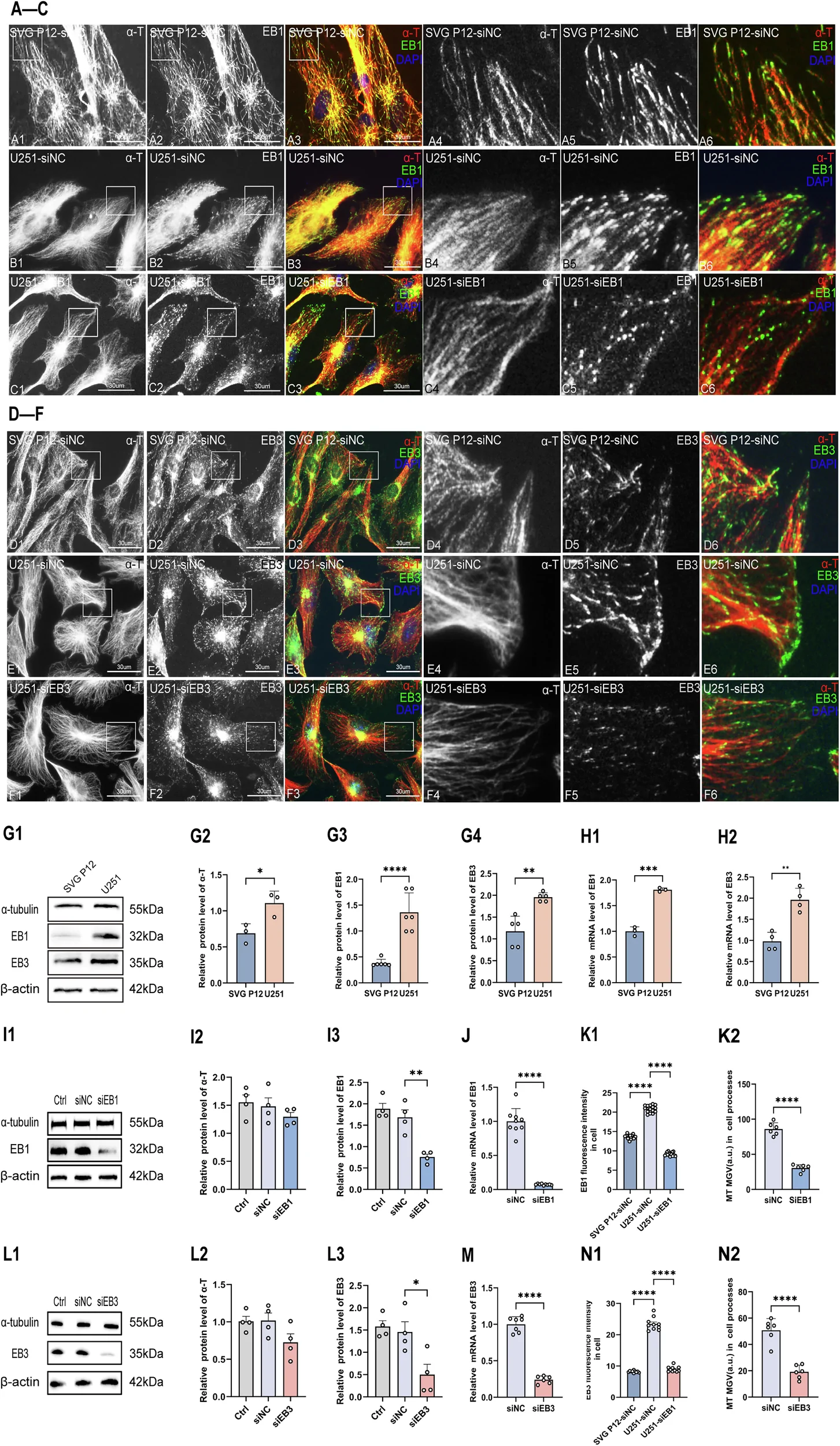
EB1 and EB3 plus-end-associated patterns in SVG P12 cells, U251-siNC cells, and EB1/EB3-silenced U251 cells. Representative IF images showing EB1 (green) and α-tubulin (red) in SVG P12-siNC cells (**A**), U251-siNC cells (**B**), and U251-siEB1 cells (**C**). Representative IF images showing EB3 (green) and α-tubulin (red) in SVG P12-siNC cells (**D**), U251-siNC cells (**E**), and U251-siEB3 cells (**F**). In SVG P12-siNC cells, EB1 and EB3 display characteristic comet-like patterns at microtubule plus-end-associated regions. In U251-siNC cells, EB1/EB3 signals are more abundant and remain comet-like, whereas EB1 or EB3 silencing reduces microtubule content and converts these patterns into shorter, dot-like structures. **G1** Representative Western blots of α-tubulin, EB1, EB3, and β-actin in untreated SVG P12 and U251 cells. **G2**–**G4** Quantification of α-tubulin, EB1, and EB3 protein levels in untreated SVG P12 and U251 cells. **H1**, **H2** RT-qPCR analysis of EB1 and EB3 mRNA in untreated SVG P12 and U251 cells. **I1** Representative Western blots of α-tubulin, EB1, and β-actin in U251-Ctrl, U251-siNC, and U251-siEB1 cells. **I2**, **I3** Quantification of α-tubulin and EB1 protein levels after EB1 silencing. **J** RT-qPCR analysis of EB1 mRNA in U251-siNC and U251-siEB1 cells. **K1** Quantification of EB1 fluorescence intensity. **K2** Quantification of α-tubulin mean grey value in cell processes after EB1 silencing. **L1** Representative Western blots of α-tubulin, EB3, and β-actin in U251-Ctrl, U251-siNC, and U251-siEB3 cells. **L2**, **L3** Quantification of α-tubulin and EB3 protein levels after EB3 silencing. **M** RT-qPCR analysis of EB3 mRNA in U251-siNC and U251-siEB3 cells. **N1** Quantification of EB3 fluorescence intensity. **N2** Quantification of α-tubulin mean grey value in cell processes after EB3 silencing. α-Tubulin labels the underlying microtubule array and serves as a structural reference for interpreting EB1/EB3 plus-end-associated patterns. Western blotting, RT-qPCR, and fluorescence-based quantification were performed with *n* = 3 independent biological experiments unless otherwise indicated. Data are presented as mean ± SD. *P < 0.05, **P < 0.01, ***P < 0.001, ****P < 0.0001.

Because TBCB does not directly bind to microtubules and plus-end regulators typically act via end-binding proteins (EBs) [34], we next examined the localization of EB1 and EB3 in GBM cells. EB1 and EB3 signals were analyzed together with α-tubulin-labeled microtubule arrays. In SVG P12 cells, both EB1 and EB3 formed characteristic comet-like structures at microtubule plus-ends, with comet heads at the tips and tails oriented toward the MTOC (Fig. 6A, D). U251 cells displayed increased microtubule density and length, accompanied by more numerous comet-like EB1 and EB3 signals (Fig. 6B, E). This increase was not limited to subcellular redistribution alone. Baseline Western blotting and RT-qPCR analyses further showed that both EB1 and EB3 were expressed at higher levels in U251 cells than in SVG P12 cells (Fig. 6G1–G4, H1, H2). In U251 cells, corresponding knockdown of EB1 or EB3 reduced both EB abundance and the associated plus-end structural pattern, as shown by Western blotting, RT-qPCR, and fluorescence-based quantification (Fig. 6I1–I3, J, K1, K2 and Fig. 6L1–L3, M, N1, N2). Silencing EB1 or EB3 reduced both EB abundance and the associated plus-end structural pattern, resulting in sparser and more collapsed cortical α-tubulin-labeled microtubule arrays together with shorter and less abundant dot-like EB structures (Fig. 6C, F). These findings indicate that the stronger EB1/EB3 plus-end-associated accumulation observed in GBM cells reflects both altered localization patterns and increased EB1/EB3 abundance, and that EB1/EB3 perturbation disrupts plus-end-associated organization within the microtubule array.

### TBCB is associated with EB1/EB3-containing plus-end complexes in GBM cells

**Fig. 7:**
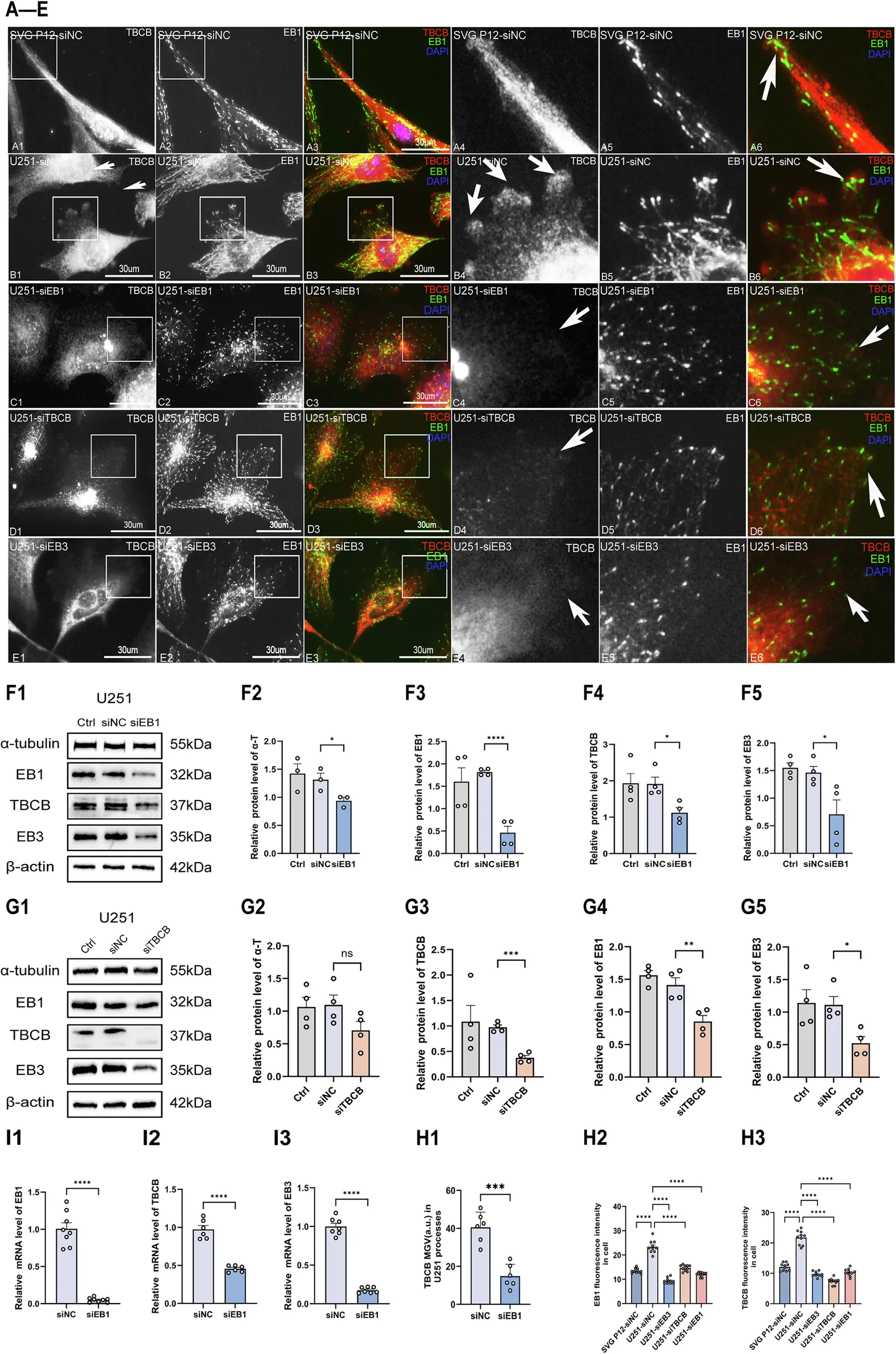
Reciprocal changes in EB1 and TBCB localization patterns after silencing of EB1, TBCB, or EB3 in U251 cells. Representative IF images showing co-expression of TBCB (red) and EB1 (green) in SVG P12-siNC cells (**A**), U251-siNC cells (**B**), U251-siEB1 cells (**C**), U251-siTBCB cells (**D**), and U251-siEB3 cells (**E**). In SVG P12-siNC cells, EB1 comet-like signals colocalize with relatively diffuse TBCB at sharp process tips. In U251-siNC cells, EB1 signals and TBCB accumulation are enhanced at obtuse process borders. Silencing of EB1, TBCB, or EB3 reduces EB1 comet-like features and weakens TBCB enrichment in cell processes. **F1** Representative Western blots of α-tubulin, EB1, TBCB, and EB3 in U251-Ctrl, U251-siNC, and U251-siEB1 cells. **F2**–**F5** Quantification of α-tubulin, EB1, TBCB, and EB3 protein levels after EB1 silencing. **G1** Representative Western blots of α-tubulin, EB1, TBCB, and EB3 in U251-Ctrl, U251-siNC, and U251-siTBCB cells. **G2**–**G5** Quantification of α-tubulin, EB1, TBCB, and EB3 protein levels after TBCB silencing. **H1** Quantification of TBCB mean grey value (MGV = integrated density/area) in U251 cell processes after EB1 silencing. **H2**, **H3** Quantification of EB1 and TBCB fluorescence intensity in the indicated groups. **I1**–**I3** RT-qPCR analysis of EB1, TBCB, and EB3 mRNA levels in U251-siNC and U251-siEB1 cells. These data support a mutually dependent relationship between EB1-associated comet-like features and TBCB enrichment at plus-end-associated regions in GBM cells. Western blotting, RT-qPCR, and fluorescence-based quantification were performed with *n* = 3 independent biological experiments unless otherwise indicated. Data are presented as mean ± SD. *P < 0.05, **P < 0.01, ***P < 0.001, ****P < 0.0001.

We then investigated whether TBCB is functionally associated with EB1/EB3-containing plus-end complexes in GBM cells. In SVG P12 cells, comet-like EB1 signals colocalized with diffusely distributed TBCB at sharp process tips (Fig. 7A). In U251 cells, EB1 comets coexisted with strong TBCB accumulation at the borders of obtuse processes (Fig. 7B). Knockdown of EB1, EB3 or TBCB each reduced the number of obtuse processes and led to smaller, less abundant EB1 comets, accompanied by weakened or lost TBCB accumulation at process tips (Fig. 7C–E). In addition to the imaging phenotypes, Western blot analyses after EB1 or TBCB silencing showed coordinated changes in α-tubulin, EB1, TBCB, and EB3 protein levels, and RT–qPCR after EB1 silencing showed corresponding reductions in EB1, TBCB, and EB3 mRNA (Fig. 7F1–F5, G1–G5, I1–I3). Process-based quantification further confirmed reduced TBCB mean grey value and altered EB1/TBCB fluorescence patterns after perturbation (Fig. 7H1–H3).

**Fig. 8:**
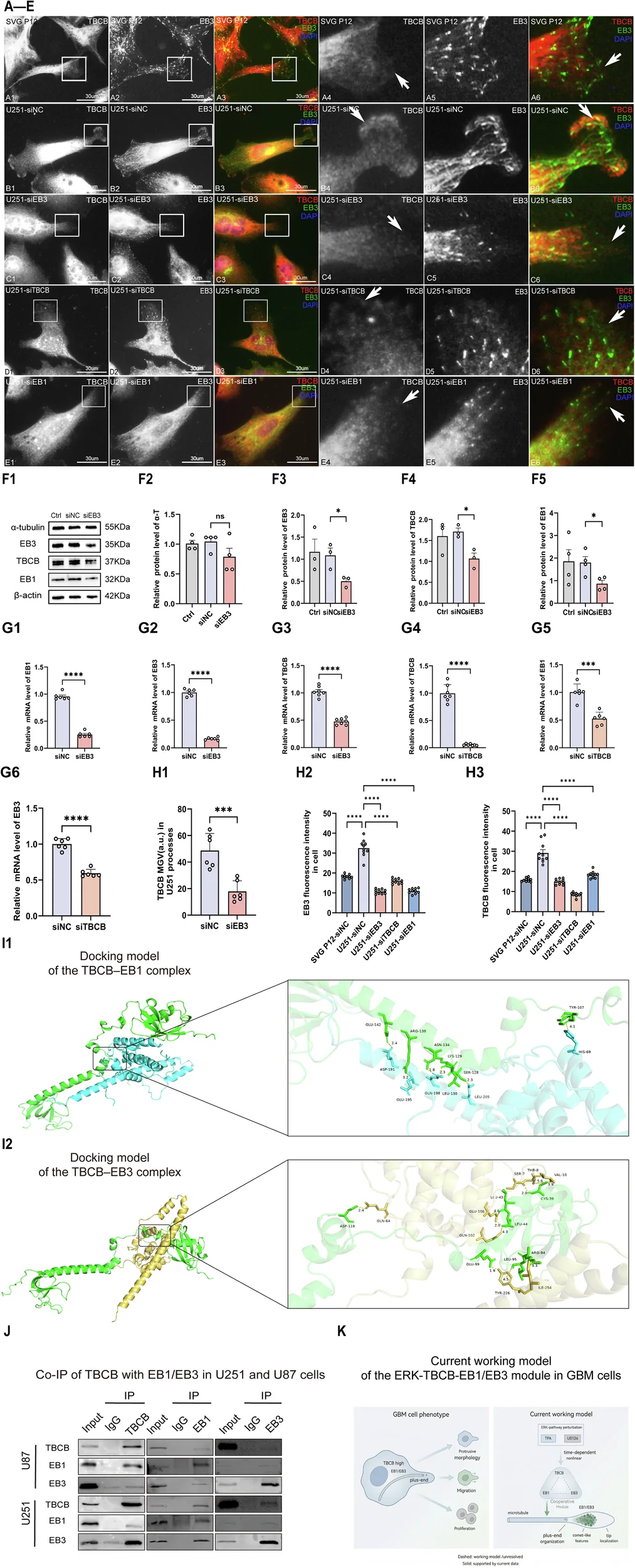
Reciprocal changes in EB3 and TBCB localization patterns after silencing of EB3, TBCB, or EB1 in U251 cells. Representative IF images showing co-expression of TBCB (red) and EB3 (green) in SVG P12 cells (**A**), U251-siNC cells (**B**), U251-siEB3 cells (**C**), U251-siTBCB cells (**D**), and U251-siEB1 cells (**E**). In SVG P12 cells, EB3 comet-like signals colocalize with TBCB at sharp process regions. In U251-siNC cells, EB3 and TBCB signals are enriched in obtuse cell processes. Silencing of EB3, TBCB, or EB1 reduces EB3 comet-like features and diminishes TBCB accumulation in cell processes. **F1** Representative Western blots of α-tubulin, EB3, TBCB, and EB1 in U251-Ctrl, U251-siNC, and U251-siEB3 cells. **F2**–**F5** Quantification of α-tubulin, EB3, TBCB, and EB1 protein levels after EB3 silencing. **G1**–**G3** RT-qPCR analysis of EB1, EB3, and TBCB mRNA levels in U251-siNC and U251-siEB3 cells. **G4**–**G6** RT-qPCR analysis of TBCB, EB1, and EB3 mRNA levels in U251-siNC and U251-siTBCB cells. **H1** Quantification of the mean grey value (MGV = integrated density/area) of TBCB in U251 cell processes after EB3 silencing. **H2**, **H3** Quantification of EB3 and TBCB fluorescence intensity in the indicated groups. **I1**, **I2** Enlarged views of the predicted TBCB–EB1 and TBCB–EB3 docking models shown as supportive structural analyses; docking scores and evaluation metrics for the top-ranked models are summarized in Supplementary Table S5. **J** Reciprocal co-immunoprecipitation (Co-IP) validation of TBCB association with EB1 and EB3 in U251 and U87 cells, including input and IgG controls. Western blotting, RT-qPCR, and fluorescence-based quantification were performed with *n* = 3 independent biological experiments unless otherwise indicated. Docking analyses are computational/supportive and are not expressed as biological replicate counts. Representative Co-IP blots are shown. Data are presented as mean ± SD where applicable. *P < 0.05, **P < 0.01, ***P < 0.001, ****P < 0.0001. **K** Refined working-model schematic summarizing the current interpretation of the ERK–TBCB–EB1/EB3-associated module in GBM cells. Elevated TBCB is associated with invasion-related GBM phenotypes, including protrusive morphology, migration, and proliferation. Current data support association of TBCB with EB1 and EB3 and support their cooperative involvement in microtubule plus-end-associated organization, comet-like features, and tip localization in GBM cells. Pharmacological perturbation with TPA and U0126 further indicates a time-dependent and nonlinear relationship between ERK-pathway perturbation and TBCB-associated regulation. Solid elements denote observations directly supported by the current data, whereas dashed elements indicate working-model or unresolved relationships.

A similar pattern was observed for EB3: EB3 comets colocalized with TBCB at sharp processes in SVG P12 cells and at obtuse processes in U251 cells, and silencing EB3, TBCB, or EB1 reduced EB3 comet size and number and diminished TBCB accumulation (Fig. 8A–E). Complementary protein and mRNA panels further showed coordinated changes in EB3, TBCB, and EB1 after EB3 or TBCB perturbation, and process-based quantification supported the reduction in TBCB mean grey value and EB3/TBCB fluorescence intensity after EB3 silencing (Fig. 8F1–F5, G1–G6, H1–H3). In parallel, these data provide reciprocal expression-level support in addition to the imaging phenotypes. Molecular docking and reciprocal co-immunoprecipitation further supported association between TBCB and EB1/EB3 (Fig. 8I, J). The top HDOCK models for TBCB–EB1 and TBCB–EB3 yielded highly favorable docking scores (approximately −270 and −260, respectively) with high confidence, consistent with plausible interaction interfaces (Supplementary Table S5). Taken together, these data support a physical and functional association among TBCB, EB1, and EB3 at microtubule plus-end-associated regions in GBM cells. The reciprocal changes in comet-like features, tip enrichment, and expression-level readouts are more consistent with mutually dependent organization than with a fully resolved linear hierarchy. Thus, the current evidence supports a cooperative plus-end-associated module rather than a resolved epistatic order among TBCB, EB1, and EB3.

### ERK signaling regulates TBCB expression in GBM cells

To identify upstream pathways controlling TBCB, we performed RNA-seq in U87 and U251 cells after TBCB knockdown and conducted Kyoto Encyclopedia of Genes and Genomes (KEGG) enrichment analysis of differentially expressed genes (absolute fold change ≥ 1.2, *P* < 0.05). Thirty-six pathways were commonly enriched in both cell lines, among which the MAPK signaling pathway was significantly represented (Fig. S1A, B), suggesting a potential link between MAPK/ERK signaling and TBCB. Western blotting revealed that phosphorylation of ERK, p38 and JNK was markedly increased in U251 cells compared with SVG P12 cells (Fig. S1C), consistent with global MAPK activation in GBM. Upon TBCB knockdown, only the p-ERK/ERK ratio was significantly reduced, whereas p-p38/p38 and p-JNK/JNK were largely unchanged (Fig. S1D), implicating ERK as the MAPK branch most closely associated with TBCB. The U87/U251 RNA-seq dataset supporting this analysis is publicly available in GSA-Human under accession HRA018476 and BioProject PRJCA053575.

We next performed a time-resolved pharmacological modulation assay in U251 cells to clarify this relationship. At the early 1-h endpoint, TPA markedly increased the p-ERK/ERK ratio and DUSP6 mRNA, whereas U0126 reduced both readouts; by contrast, TBCB protein and mRNA showed only minor early changes (Fig. S2D1–D5). At the delayed 12-h endpoint after a 1-h drug pulse and medium replacement, however, both TPA and U0126 reduced TBCB protein and TBCB mRNA despite persistent divergence in ERK-pathway readouts (Fig. S2E1–E5). Representative delayed-endpoint immunofluorescence images are shown as descriptive context in Fig. S2A–C. Together, these data indicate that ERK regulation of TBCB in GBM cells is time-dependent and nonlinear.

## Discussion

### The function of TBCB at microtubule plus-ends

Our integrative analyses support TBCB upregulation as a consistent molecular feature of malignant glioma and motivate a cytoskeleton-centered interpretation of its function in GBM. Beyond its established role in tubulin folding and microtubule turnover, excess TBCB has been reported to destabilize tubulin heterodimers and newly assembled microtubules, resulting in microtubule depolymerization and impaired neurite/growth-cone integrity [8, 10]. In the context of GBM, our observations are compatible with a model in which elevated TBCB perturbs EB-associated plus-end organization within nascent cellular processes, thereby facilitating the formation of atypical, invasion-associated protrusions.

Specifically, GBM cells exhibited aberrant microtubule organization within processes, including curled or disordered plus-ends, concomitant with increased TBCB accumulation at process tips. In contrast, TBCB depletion restored a more “astrocyte-like” morphology characterized by sharper processes and more orderly microtubule arrays. These findings suggest that appropriate TBCB dosage is required to maintain balanced plus-end-associated organization and orderly microtubule arrangement, and that pathological elevation of TBCB may shift these structural features toward unstable or misdirected patterns that support process remodeling and cell motility. The current GBM dataset therefore supports a requirement for elevated TBCB in maintaining protrusive morphology and plus-end-associated organization in a malignant cellular background, but it does not establish that TBCB overexpression alone is sufficient to impose a GBM-like phenotype on normal astrocytes.

Notably, TBCB preferentially accumulated in nascent processes rather than mature elongated processes, and manipulating TBCB levels altered the number of newly formed protrusions. This spatial restriction argues that TBCB acts early during protrusion initiation/extension, potentially by shaping local plus-end-associated organization and coordinating the recruitment of additional +TIPs and membrane-proximal regulators. Although our imaging suggests TBCB is positioned close to the cell cortex relative to EB signals, whether TBCB directly guides membrane-associated complexes or influences actin–microtubule crosstalk remains to be determined.

### TBCB enrichment at MTOC-related regions is associated with GBM proliferative capacity

Microtubule-organizing centers (MTOCs) anchor microtubule minus ends and coordinate centrosome-dependent microtubule nucleation, functions that are closely linked to proliferative behavior and mitotic organization [34–37]. TBCB has been reported to localize to centrosomes and spindle-related structures [14], providing a plausible basis for its enrichment at MTOC-related regions in GBM cells. In our study, strong TBCB signal at MTOC-related regions in U251 cells coincided with higher proliferative capacity, whereas TBCB knockdown reduced EdU incorporation, slowed cell growth, and shifted cell-cycle distribution toward an increased G0/G1 fraction with a decreased S-phase fraction, without a significant change in G2/M.

These findings support an association between TBCB enrichment at MTOC-related regions and GBM proliferative capacity, and are consistent with altered cell-cycle progression after TBCB depletion. However, the current dataset does not establish a specific mitotic defect or define the precise checkpoint or cell-cycle step regulated by TBCB. In particular, the lack of a significant G2/M accumulation argues against concluding a dominant mitotic arrest based on the present data alone.

Future studies combining refined cell-cycle assays, mitotic-marker analysis, and direct assessment of centrosome/spindle architecture will be required to determine whether TBCB primarily affects G1/S entry, S-phase progression, or other aspects of proliferative control in GBM cells.

### TBCB, EB1 and EB3 likely form a cooperative plus-end-associated module in GBM cells

Our data indicate that TBCB, EB1 and EB3 are closely associated at microtubule plus-end-associated regions in GBM cells, and that perturbation of any one component is accompanied by coordinated changes in the localization features of the others. Knockdown of TBCB, EB1 or EB3 each reduced comet-like features and suppressed obtuse process formation, supporting a cooperative relationship among these proteins in organizing plus-end-associated structures. EB family members can form heterodimers and coordinate the recruitment of multiple +TIP-associated partners [38, 39]; within this framework, EB1/EB3-containing assemblies may provide part of a cooperative structural context for TBCB enrichment at plus-end-associated regions, although the precise recruitment order remains unresolved.

Mechanistically, docking analysis suggests that the C-terminal CAP-Gly domain of TBCB can engage the C-terminal EEY/F motif of EB1/EB3, consistent with a plausible physical interface. Reciprocal Co-IP provides the main biochemical support for association between TBCB, EB1, and EB3 in GBM cells. Taken together, these data support a physical and functional association among TBCB, EB1, and EB3 at plus-end-associated regions, but do not define assembly order or stoichiometry. The coordinated changes observed in EB1, EB3, and TBCB at the mRNA/protein level are unlikely to reflect direct one-to-one transcriptional regulation. Instead, they are more consistent with indirect, network-level coupling within a cooperative and dosage-sensitive plus-end-associated module. Because EB1/EB3 act as core +TIP adaptors and TBCB is linked to tubulin availability and plus-end-associated organization, perturbation of any one component may alter local retention at microtubule tips, broader cytoskeletal homeostasis, and secondary feedback to transcript and protein levels. In this sense, the coordinated expression changes observed here are more compatible with indirect coupling within an integrated cytoskeletal module than with a simple linear upstream–downstream relationship. This interpretation is also consistent with the context-dependent behavior of TBCB dosage: in our GBM model, TBCB is already aberrantly upregulated, and in previous fixed-cell astrocyte studies from our group, perturbation of EB1/EB3 was accompanied by synchronous changes in TBCB-associated plus-end patterns, whereas TBCB overexpression induced abnormal obtuse or “huge” nascent processes rather than a simple enhancement of normal protrusions. Together, these observations argue that excessive TBCB does not merely strengthen a normal pathway, but may instead distort the balance of plus-end-associated assemblies.

Resolving whether TBCB primarily affects EB residence, +TIP composition, tubulin availability, or other aspects of plus-end-associated organization, and determining how these structural changes feed back to transcript and protein levels, will require bidirectional rescue/epistasis analysis, structure–function interrogation of the CAP-Gly/EEY interface, and quantitative live-cell imaging. Taken together, the current data support a cooperative working model for the TBCB–EB1–EB3 axis rather than a fully resolved linear pathway. This association-level working model is summarized schematically in Fig. 8K.

### ERK signaling regulates TBCB expression in GBM cells

Transcriptome profiling and pathway enrichment implicated MAPK signaling as a prominent pathway associated with TBCB perturbation, and biochemical analyses indicated that ERK activity is tightly coupled to TBCB expression in GBM cells. MAPK/ERK signaling is frequently hyperactivated in glioma and is known to regulate cytoskeletal remodeling, migration, and proliferation through multiple downstream effectors. In our experiments, GBM cells displayed elevated baseline MAPK activation, and TBCB knockdown selectively reduced ERK phosphorylation without markedly altering p38 or JNK branches, supporting a preferential association between TBCB and ERK signaling.

Interestingly, time-resolved analysis indicated that the ERK–TBCB relationship in GBM cells is nonlinear and strongly context-dependent. At 1 h, TPA increased p-ERK/ERK and DUSP6, whereas TBCB mRNA and protein changed only modestly. At the delayed 12-h endpoint, both TPA and U0126 reduced TBCB mRNA and protein despite divergent ERK readouts, indicating that deviation of ERK activity in either direction can converge on delayed TBCB downregulation. In our previous fixed-cell astrocyte studies, ERK activation was associated with increased TBCB expression and enhanced nascent process formation in a non-neoplastic background. Together, these findings suggest that ERK control of TBCB-associated morphology is context-dependent and may be reshaped in GBM by altered signaling setpoints, feedback circuitry, and cytoskeletal remodeling. The DUSP6 response after TPA is consistent with feedback engagement, but because p-ERK/ERK and DUSP6 remained elevated at the delayed endpoint while TBCB decreased, the present data do not support a simple model in which TPA-induced TBCB downregulation is explained solely by canonical upstream feedback suppression of ERK signaling. Additional studies will be required to distinguish delayed transcriptional repression from altered RNA stability or protein turnover. Consistent with this interpretation, the time-resolved U251 experiments showed early divergence in ERK-pathway readouts but delayed convergent downregulation of TBCB. In this schematic summary, ERK-pathway perturbation is depicted as a time-dependent and nonlinear influence on the TBCB–EB1–EB3-associated module rather than as a fully resolved one-way hierarchy (Fig. 8K).

Conversely, TBCB-mediated microtubule remodeling may also feed back to ERK signaling. Microtubules can organize signaling complexes and influence mechanotransduction-sensitive pathways through changes in cell shape, adhesion, cortical tension, and scaffold-associated localization of kinases or phosphatases. In this context, altered TBCB/EB1/EB3-associated plus-end organization could influence ERK-pathway output indirectly by changing the spatial organization of scaffold-associated signaling components at protrusive fronts or MTOC-related regions. Our observation that TBCB knockdown reduces p-ERK/ERK is consistent with reciprocal cytoskeleton-signaling coupling, although the current data do not distinguish whether ERK changes arise from direct biochemical regulation, altered cytoskeletal mechanics, or scaffold-dependent signal redistribution. We therefore interpret the ERK–TBCB relationship as potentially bidirectional and context-dependent rather than as a simple one-way upstream pathway.

From a translational perspective, the ERK–TBCB–EB1/EB3-associated cytoskeletal module identified here provides a framework for future studies of treatment response in GBM. It will be important to determine whether TBCB status influences sensitivity or adaptive responses to standard chemoradiotherapy, microtubule-directed approaches, and MEK/ERK-pathway modulation. Given the association of this axis with protrusive morphology and invasion-related behavior, future work should also test whether its perturbation contributes to treatment persistence or migratory escape after therapy.

### Limitations and future directions

This study has several limitations. First, although we established a mechanistic association between TBCB, EB1, and EB3 through reciprocal co-immunoprecipitation, docking analysis, and coordinated localization changes, the current data do not yet resolve the full directionality or temporal hierarchy of these interactions at microtubule plus ends. Future live-cell imaging and perturbation studies will be needed to define how TBCB influences EB-comet behavior and microtubule organization in real time. Second, although our GBM data support the necessity of TBCB for maintaining the elongated, process-bearing phenotype of glioblastoma cells, they do not establish sufficiency in a normal astrocyte background within the current study. Therefore, although the contrast discussed here is informed by our previous astrocyte work showing ERK-associated increases in TBCB and nascent process formation, it does not substitute for a direct within-study normal-versus-GBM comparison. Third, although our time-resolved analyses indicate that the ERK–TBCB relationship is context-dependent and nonlinear in GBM cells, the upstream regulatory circuitry and downstream morphological effectors remain incompletely defined. Future work will be needed to determine whether the delayed TBCB decrease after ERK-pathway perturbation is mediated predominantly by transcriptional control, altered RNA stability, post-translational turnover, or a combination of these mechanisms. Fourth, the orthotopic xenograft experiments were performed in BALB/c nude mice, which lack an intact adaptive immune system and do not fully reproduce the immune microenvironment of human GBM. Accordingly, the current in vivo data support effects on tumor growth and survival, but do not address immune-microenvironment-dependent invasion, tumor-immune crosstalk, or immune-dependent treatment responses. In particular, this model does not allow us to evaluate how TBCB-associated cytoskeletal remodeling may interact with tumor-associated macrophages/microglia (TAMs), myeloid cell recruitment, or TAM-mediated extracellular matrix remodeling and invasion. These immune and stromal interactions will require immunocompetent or humanized GBM models in future studies. Our study did not include pharmacological targeting of TBCB or in vivo therapeutic intervention experiments; therefore, any translational implication regarding TBCB as a drug target should be considered preliminary. Future work should examine whether the ERK–TBCB–EB1/EB3 axis influences treatment responses, adaptive resistance, and cell-death programs in GBM, particularly in the context of standard chemoradiotherapy, microtubule-directed approaches, and MEK/ERK-pathway modulation.

## Materials and methods

### Cell culture

Human fetal glial cells (SVG P12) and human glioblastoma cell lines (U87 and U251) were obtained from the Cell Bank of the Chinese Academy of Sciences (Shanghai, China). Cells were cultured in high-glucose Dulbecco’s modified Eagle’s medium (DMEM; Gibco, Thermo Fisher Scientific; Cat. No. 11995065) supplemented with 10% fetal bovine serum (FBS; GeminiBio; Cat. No. 100500) and 1% penicillin–streptomycin (Beyotime, Shanghai, China; Cat. No. C0222). Cells were maintained at 37 °C in a humidified incubator with 5% CO₂ and were used within 10 passages. Cell line authentication by STR profiling and routine mycoplasma testing were performed (documentation available).

### Small interfering RNA (siRNA) transfection

Cells were transfected with siRNA oligonucleotides using Lipofectamine™ 3000 (Invitrogen, Thermo Fisher Scientific, USA) for 48–72 h in 6-well or 24-well plates according to the manufacturer’s instructions. For each well of a 6-well plate, 5 μL siRNA and 3.75 μL Lipofectamine 3000 were mixed in 250 μL Opti-MEM (Gibco, USA). For each well of a 24-well plate, 1 μL siRNA and 0.75 μL Lipofectamine 3000 were mixed in 50 μL Opti-MEM. A non-targeting siRNA (siNC) served as the negative control. 48 h after transfection, cells were harvested for qRT-PCR analysis. 72 h after transfection, cells were harvested for Western blotting and immunofluorescence analyses. siRNAs were synthesized by GenePharma (China), and sequences are provided in Supplementary Table S1.

### Lentiviral infection

U251 and U87 cells were infected with lentiviruses for stable TBCB knockdown or control shRNA as indicated. shTBCB or control shRNA (shNC) lentiviruses were used at an MOI of 2, and cells were exposed to virus for 48 h in the presence of polybrene (8 μg/mL). Stable cell populations were selected with puromycin (U87: 5 μg/mL; U251: 4 μg/mL) for 4 days according to the supplier’s instructions. Where indicated (e.g., luciferase-labeled cells), blasticidin selection was performed (U87: 10 μg/mL; U251: 6 μg/mL) for 7 days. Target sequences are listed in Supplementary Table S2.

### Immunofluorescent staining and hematoxylin–eosin (H&E) staining

Human tissue specimens and ethics. Human glioblastoma (GBM) tumor tissues (*n* = 3) and adjacent non-tumor brain tissues (*n* = 3) were obtained from patients undergoing surgical resection at our center. Histopathological diagnoses were established according to the World Health Organization (WHO) 2024 classification of tumors of the central nervous system. The study protocol, including the application for a waiver of informed consent, was reviewed and approved by the Clinical Research Ethics Committee of the First Affiliated Hospital of Chongqing Medical University (Chongqing, China; Approval No. K2024-166-01). All procedures were conducted in accordance with the Declaration of Helsinki and relevant institutional guidelines.

The paraffin sections were deparaffinized, rehydrated and boiled. Sections were permeabilized with 0.5% Triton X-100 at 37 °C for 30 min. Afterward, the sections were blocked in 5% bovine serum albumin (BSA) for 1 h at 37 °C, incubated with the primary antibodies (details are provided in Supplementary Table S3) at 4 °C overnight and secondary antibodies (FITC goat anti-rabbit IgG; FITC goat anti-mouse IgG; Cy3 goat anti-mouse IgG; Cy3 goat anti-rat IgG; EARTH, China) for 1 h at room temperature. Sections were then stained with DAPI (C1005, Beyotime, Shanghai, China) and mounted in Fluorescence Mounting Medium (ab104135, Abcam, Cambridge, UK). Cellular IF staining was performed as previously described [40]. Images were acquired using an inverted fluorescence microscope (Leica DMI8, Germany), and fluorescence intensity was analyzed with ImageJ (version 1.53c).

For hematoxylin–eosin (H&E) staining, paraffin-embedded brain tissue sections were deparaffinized, rehydrated, stained with hematoxylin and eosin using a routine protocol, then dehydrated, cleared and mounted. H&E stained sections were observed and photographed under a bright-field microscope (representative images are shown in Fig. 2D).

### Western blotting

Total cellular protein was extracted from cells using RIPA buffer (Beyotime, China) in the presence of PMSF (Beyotime, China). Protein concentration was measured using a BCA Protein Assay Kit (Beyotime, China). Western blotting was performed according to a previous study [40]. Proteins were transferred onto PVDF membranes (Millipore; 0.22 μm; Cat. No. ISEQ00010). Specific signals were visualized using WesternBright ECL HRP substrate (Advansta, USA) and detected using a BioRad imaging system (BioRad, USA). Band quantification and normalization were performed using standard procedures.

### Quantitative real-time PCR

qRT-PCR was performed as previously described [40]. Total RNA was reverse-transcribed using PrimeScript™ RT reagent Kit with gDNA Eraser (Takara; Cat. No. RR036A). qPCR was performed using TB Green® Premix Ex Taq™ II (Tli RNaseH Plus) (Takara; Cat. No. CN830A) according to the manufacturer’s instructions. Primer sequences are listed in Supplementary Table S4.

### EdU incorporation assay

Cell proliferation was assessed using a Cy3-labeled EdU Cell Proliferation Assay Kit (K1075, APExBIO). U87 and U251 cells were seeded onto glass coverslips in 24-well plates with 500 μL complete medium containing 10% FBS per well. Upon reaching the logarithmic growth phase, a 10 μM EdU working solution was prepared by diluting a 10 mM EdU stock solution 1:1000 according to the manufacturer’s instructions. Cells were incubated with EdU at 37 °C for 2 h. Fixation, permeabilization and click reaction were performed following the kit protocol. Hoechst dye provided in the kit was used for nuclear counterstaining. Images were acquired using an inverted fluorescence microscope (Leica). For quantification, 5 random fields per group were analyzed, and experiments were repeated three times independently.

### Cell counting assay

Cell growth curves were generated using an automatic cell counting system. U87 and U251 cells transfected with siTBCB or control siRNA (siNC) were seeded into 24-well plates at a density of 5.0 × 10⁴ cells/well in 500 μL complete medium (defined as 0 h). Cells were harvested every 24 h for 3 consecutive days. After resuspension, viable cells were counted using a disposable cell counting slide (RWD DS50) and an automatic cell counter (RWD C100). Experiments were performed with three independent biological replicates.

### Cell-cycle analysis by flow cytometry

Cell-cycle distribution was assessed using a Cell Cycle Analysis Kit (red fluorescence; Elabscience, Cat. No. E-CK-A351) according to the manufacturer’s instructions. U251 and U87 cells transfected with siNC or siTBCB were collected for analysis. Approximately 5 × 10^5 cells per sample were harvested, centrifuged at 300 × g for 5 min, washed once with PBS, and resuspended in 0.3 mL PBS. Cells were then fixed by slow dropwise addition of 1.2 mL pre-chilled absolute ethanol and stored at −20 °C overnight.

After fixation, cells were centrifuged at 300 × g for 5 min to remove the fixative, resuspended in 1 mL PBS, and rehydrated at room temperature for 15 min. The cells were then pelleted again and incubated with 100 μL RNase A at 37 °C for 30 min, followed by the addition of 400 μL PI staining solution (50 μg/mL). Samples were mixed thoroughly and incubated in the dark at 2–8 °C for 30 min before flow-cytometric analysis.

Fluorescence signals were acquired on a Sony SA3800 flow cytometer using 488 nm excitation. For each sample, 2 × 10^4 events were collected. The raw data were analyzed using FlowJo software (version 10.8), and the percentages of cells in G0/G1, S, and G2/M phases were quantified using standard DNA-content modeling. Three independent biological replicates were performed for each condition.

### Transwell migration assay

Cell migration was assessed using Transwell inserts (8.0 μm pore size; Corning, NY, USA). A total of 3 × 10⁴ cells were seeded into the upper chamber in 200 μL DMEM containing 2% FBS. The lower chamber contained 700 μL DMEM with 20% FBS. After 24 h, non-migrating cells were removed from the upper surface, and migrated cells on the lower surface were fixed for 15 min and stained with 0.1% crystal violet solution (Solarbio, China) for 30 min. Five random fields per insert were imaged, and cell numbers were quantified using ImageJ. Experiments were repeated three times independently. Images were acquired using an inverted microscope (TCS SP5; Leica, Germany).

### Wound-healing assay

U87 and U251 cells were cultured in 6-well plates to approximately 80–90% confluence. A sterile 200 μL pipette tip was used to create a scratch wound in the center of each well. Cells were then cultured in medium containing 1% FBS to minimize proliferation-associated effects on wound closure. Images were captured at 0 h, 24 h and 48 h under an inverted microscope (TCS SP5; Leica, Germany). Wound closure was quantified using ImageJ as the percentage of remaining wound area relative to 0 h. Experiments were repeated three times independently. For wound-edge morphometric analysis, bright-field or phase-contrast images were acquired from the wound-edge region at 24 h after scratching. In each independent experiment, five random wound-edge fields were captured per group using identical microscope settings. Only cells located within approximately 0–150 μm of the wound edge, with clearly discernible boundaries and without obvious overlap with neighboring cells, were included in the analysis; mitotic cells and cells that were shrunken, fragmented, detached, or out of focus were excluded. A cell process was defined as a slender protrusion extending from the cell-body margin with a clearly traceable contour and a length of at least 10 μm. After scale calibration in Fiji/ImageJ, the number of processes per cell was counted manually. Process length was measured using the segmented-line tool along the central axis of each protrusion, from the point at which the process emerged from the cell body to the most distal clearly identifiable tip. All qualifying protrusions of each cell were measured, and the longest process length per cell was recorded. At least 30 cells per group were analyzed in each independent experiment, and three independent experiments were performed. Process number per cell and longest process length per cell were used as the quantitative endpoints. Data are presented as mean ± SD, and comparisons between two groups were performed using two-sided Student’s t-tests.

### Drug treatment

U251 cells were treated with vehicle (Vehicle, A), TPA, or U0126 for 1 h. For the early endpoint, cells were collected immediately after the 1-h treatment. For the delayed endpoint, drug-containing medium was removed after 1 h, replaced with fresh complete medium, and cells were collected 12 h later. Protein lysates were analyzed by Western blotting for ERK, p-ERK, TBCB, and β-tubulin. RNA was extracted in parallel for RT-qPCR analysis of DUSP6 and TBCB. Statistical comparisons among Vehicle, TPA, and U0126 groups were performed separately at each time point using one-way ANOVA followed by Dunnett’s multiple-comparisons test.

### Co-immunoprecipitation (Co-IP) and IP-MS proteomics

Cells were lysed on ice for ~30 min using Western & IP Cell Lysis Buffer (Beyotime; Cat. No. P0013J) supplemented with a protease inhibitor cocktail (Beyotime; Cat. No. P1005), followed by centrifugation at 20,000 × g for 15 min at 4 °C. The supernatant was collected and incubated with anti-TBCB or anti-EB1/EB3 antibodies (or isotype control IgG) with rotation at 4 °C for at least 2 h (up to overnight). Protein A/G magnetic beads (MCE; Protein A/G Magnetic Beads; Cat. No. HY-K0202) were added and incubated with rotation at 4 °C for ≥2 h or overnight. Beads were collected using a magnetic rack and washed at least three times with lysis buffer, followed by PBS washes to reduce nonspecific binding. Immunoprecipitated proteins were eluted in SDS loading buffer, resolved by SDS-PAGE, and analyzed by Western blotting. Reciprocal Co-IP was performed in U251 and U87 cells, and input and IgG controls were included in each experiment.

For TBCB-bait IP-MS/AP-MS experiments, U87 and U251 cell lysates were subjected to immunoprecipitation using anti-TBCB antibody, with IgG/control immunoprecipitations processed in parallel. Immunoprecipitated proteins were prepared for LC-MS/MS analysis by the service provider using standard proteomics workflows, including enzymatic digestion, liquid chromatography-tandem mass spectrometry on a Q Exactive HF-X instrument, and database searching. Candidate TBCB-associated proteins were prioritized based on enrichment relative to control immunoprecipitations and consistency with microtubule plus-end-associated biology. The resulting Homo sapiens TBCB-bait AP-MS dataset has been deposited in ProteomeXchange via the iProX partner repository under ProteomeXchange accession PXD077829, iProX Project ID IPX0016905000, and iProX Subproject ID IPX0016905001.

### RNA-seq and bioinformatic analysis

Total RNA extraction, library construction and sequencing were performed by Personalbio (Shanghai, China) using standard strand-specific mRNA-seq protocols on an Illumina NovaSeq 6000 platform. For the human glioblastoma cell-line RNA-seq dataset, U87 and U251 cells from NC control, TBCB-related and EB1-related perturbation groups were sequenced as paired-end FASTQ files. Raw reads were filtered with fastp (version 0.22.0) [41], and clean reads were aligned to the appropriate mouse or human reference genome using HISAT2 (version 2.1.0) [42]. Gene-level read counts were obtained with HTSeq (version 0.9.1) [43]. Differentially expressed genes (DEGs) were identified in R (R Foundation for Statistical Computing, Vienna, Austria) [44] using DESeq2 (version 1.38.3) [45]. For the DEG screening used in the present study, genes with an absolute fold change ≥ 1.2 and a nominal *P* value < 0.05 were considered significant. The human U87/U251 RNA-seq data are publicly available in GSA-Human under accession HRA018476 and BioProject PRJCA053575. The HT22 mouse RNA-seq dataset used for disease-enrichment analysis has been submitted to GSA under BioProject PRJCA053575 and Submission ID subCRA057806; the final public CRA accession will be updated after completion of database file processing.

Pathway enrichment analysis of DEGs was performed using clusterProfiler (version 4.6.0) [46]. KEGG pathway over-representation analysis [47, 48] was carried out with a hypergeometric test and Benjamini–Hochberg adjustment, and pathways with *P* < 0.05 were regarded as significantly enriched. For the HT22 transcriptome dataset, three groups were analyzed: untreated control cells (Ctrl), control lentivirus-transduced cells (shNC), and TBCB-targeting lentivirus-transduced cells (shTBCB). The gene set used for downstream disease-enrichment analysis consisted of DEGs shared by the shTBCB vs Ctrl and shTBCB vs shNC comparisons, excluding genes identified in the shNC vs Ctrl comparison. Disease enrichment analysis of this gene set was performed against the DisGeNET database of curated human gene-disease associations [49] using the enricher function in clusterProfiler. Unless otherwise stated, transcriptomic and enrichment analyses were carried out on the Personalbio GenesCloud platform.

### Intracranial xenograft and in vivo imaging

Six-week-old female BALB/c nude mice (~20 g) were housed under specific pathogen-free (SPF) conditions. All animal experiments were approved by the Animal Ethics Committee of Chongqing Medical University (Approval No. IACUC-CQMU-2024-0987). U87 and U251 cells were collected after transduction with shTBCB or control shRNA (shNC) lentiviruses, washed and resuspended in sterile PBS.

Mice were anesthetized with tribromoethanol (0.2 mL/10 g, intraperitoneal injection) and fixed in a stereotaxic apparatus. The cell suspension (total volume 5 μL) was injected into the right frontal lobe using stereotaxic coordinates relative to bregma: anteroposterior (AP) + 0.5 mm, mediolateral (ML) + 1.0 mm, and dorsoventral (DV) − 3.5 mm. The injection rate was 1 μL/min; the needle was left in place for 5 min after injection and then slowly withdrawn. The burr hole was sealed with bone wax. The inoculation dose was 5 × 10⁵ cells/mouse for U87 cells and 3 × 10⁵ cells/mouse for U251 cells.

Tumor growth was monitored by in vivo bioluminescence imaging using an IVIS system (Revvity; IVIS Spectrum 2). Mice were anesthetized with isoflurane for imaging. D-luciferin sodium salt (Meilunbio; CAS No. 103404757) was dissolved in sterile DPBS (without Ca²⁺/Mg²⁺) to prepare a 15 mg/mL working solution, sterilized through a 0.2 μm filter, and used immediately or stored in aliquots at −20 °C/ − 80 °C protected from light. D-luciferin was administered at 150 mg/kg (10 μL/g body weight; e.g., intravenous injection using a 25–27 G needle) prior to imaging. Images were acquired ~10 min later using consistent exposure parameters. Signal intensity was quantified by defining ROIs over the intracranial luminescent area and measuring photon flux (photons/s/cm²/sr). Animals were randomized to groups (*n* = 6 per group), and ROI analysis was performed in a blinded manner. Humane endpoints included sustained hunching, markedly reduced activity, a pronounced decrease in food intake, body weight loss > 20% from baseline, or severe neurological deficits. When endpoints were reached, mice were euthanized after overdose anesthesia followed by cervical dislocation.

### Database and public bioinformatic analysis

Transcriptomic and clinical data of glioma patients were obtained from public databases, including TCGA, CGGA, and the GEO dataset GSE243682. For GSE243682, raw count data and sample annotations were downloaded, and TBCB mRNA expression was calculated as log₂(count + 1). Samples were classified as GBM or normal brain tissue based on sample source descriptions, and TBCB expression was compared using the Wilcoxon rank-sum (Mann–Whitney) test. For TCGA and CGGA, normalized RNAseq expression matrices and corresponding clinical information (e.g., WHO grade, IDH status, progression type, overall survival) were used as provided by the databases. Associations between TBCB expression and clinicopathological variables were assessed using one-way ANOVA with multiple comparisons (for normally distributed data with equal variances) or Kruskal-Wallis test with Dunn’s multiple comparisons (for non-normal data) for comparisons across three or more groups (e.g., WHO grades), as appropriate. Overall survival was analyzed using the Kaplan–Meier method, and differences between high and low TBCB expression groups (dichotomized by the median expression within each cohort) were evaluated using two-sided log-rank tests.

All public database analyses and visualizations were performed in R [44] using standard statistical and plotting packages, including survival and survminer [50, 51]. CGGA data were downloaded from the official CGGA portal, and TCGA data were downloaded from the official TCGA portal.

### Protein structure prediction and molecular docking

The three-dimensional structures of TBCB and EB1/EB3, as well as their putative complexes, were predicted using AlphaFold3 (version 202507-05) [52]. Protein–protein docking between TBCB and EB1 or EB3 was performed with the HDOCK web server [53] using default parameters. The resulting docking models were evaluated using the PDBePISA server [54] to analyze interfacial interactions and oligomeric assemblies. Docking scores and evaluation metrics for the top-ranked complexes are summarized in Supplementary Table S5. Structural visualization and figure preparation were carried out using PyMOL (Schrödinger, LLC).

### Statistical analysis

All quantitative data are presented as mean ± standard deviation (SD) unless otherwise specified. For in vitro experiments, *n* denotes the number of independent biological replicates; for animal experiments, *n* denotes the number of animals per group. For wound-edge morphometric analysis, individual cell measurements were first summarized within each independent experiment, and these experiment-level values were used for statistical comparisons. Sample sizes (*n*) are indicated in the figure legends. All statistical tests were two-sided, and *P* < 0.05 was considered statistically significant. Comparisons between two groups were performed using unpaired Student’s t-tests for approximately normally distributed data or the Mann–Whitney U test for non-normally distributed data. For comparisons among three or more groups, one-way ANOVA followed by appropriate post hoc tests was applied. Survival curves were generated using the Kaplan–Meier method and compared using two-sided log-rank tests. Graphs and statistical analyses of experimental data were performed using GraphPad Prism 9 (GraphPad Software, San Diego, CA, USA). Statistical analyses of public datasets (GEO, TCGA, CGGA) were carried out in R (version 4.3.1) [44] as described in Section “Database and public bioinformatic analysis”.

### Ethics

The use of human tissue specimens was reviewed and approved by the Clinical Research Ethics Committee of the First Affiliated Hospital of Chongqing Medical University (Approval No. K2024-166-01). All animal experiments were approved by the Animal Ethics Committee of Chongqing Medical University (Approval No. IACUC-CQMU-2024-0987). The requirement for informed consent was waived by the Clinical Research Ethics Committee of the First Affiliated Hospital of Chongqing Medical University (Approval No. K2024-166-01).

## Supplementary information

Supplementary Figure Legends

Supplemental Material Figure 1

Supplemental Material Figure 2

Supplementary_Material_WB

Supplemental Table

## Data Availability

The human U87/U251 RNA-seq raw data generated in this study are publicly available in the Genome Sequence Archive for Human (GSA-Human), National Genomics Data Center, under accession HRA018476 and BioProject PRJCA053575 (https://ngdc.cncb.ac.cn/gsa-human/browse/HRA018476). This record was released on 13 May 2026 and contains 12 public runs comprising 24 paired-end RNA-seq FASTQ files generated on the Illumina NovaSeq 6000 platform. The HT22 mouse RNA-seq dataset used for disease-enrichment analysis has been submitted to the Genome Sequence Archive (GSA) under BioProject PRJCA053575 and Submission ID subCRA057806; at the time of final-file submission, file/database processing had been completed for eight runs and remained ongoing for one run (CRR2454624/bc3), and the final public CRA accession will be updated after completion of database file processing. The U87/U251 TBCB-bait IP-MS proteomics data have been deposited in the ProteomeXchange Consortium via the iProX partner repository [55, 56] under ProteomeXchange accession PXD077829, iProX Project ID IPX0016905000, and iProX Subproject ID IPX0016905001. The iProX record is currently private, and public release will be enabled upon publication if required.
