# Supplementary material for: The role and mechanism of tubulin folding cofactor B in glioblastoma migration
Source: Cell Death Discov. 2026 Jun 30;12:376. doi: 10.1038/s41420-026-03232-6 (PMC13586178; doi:10.1038/s41420-026-03232-6)

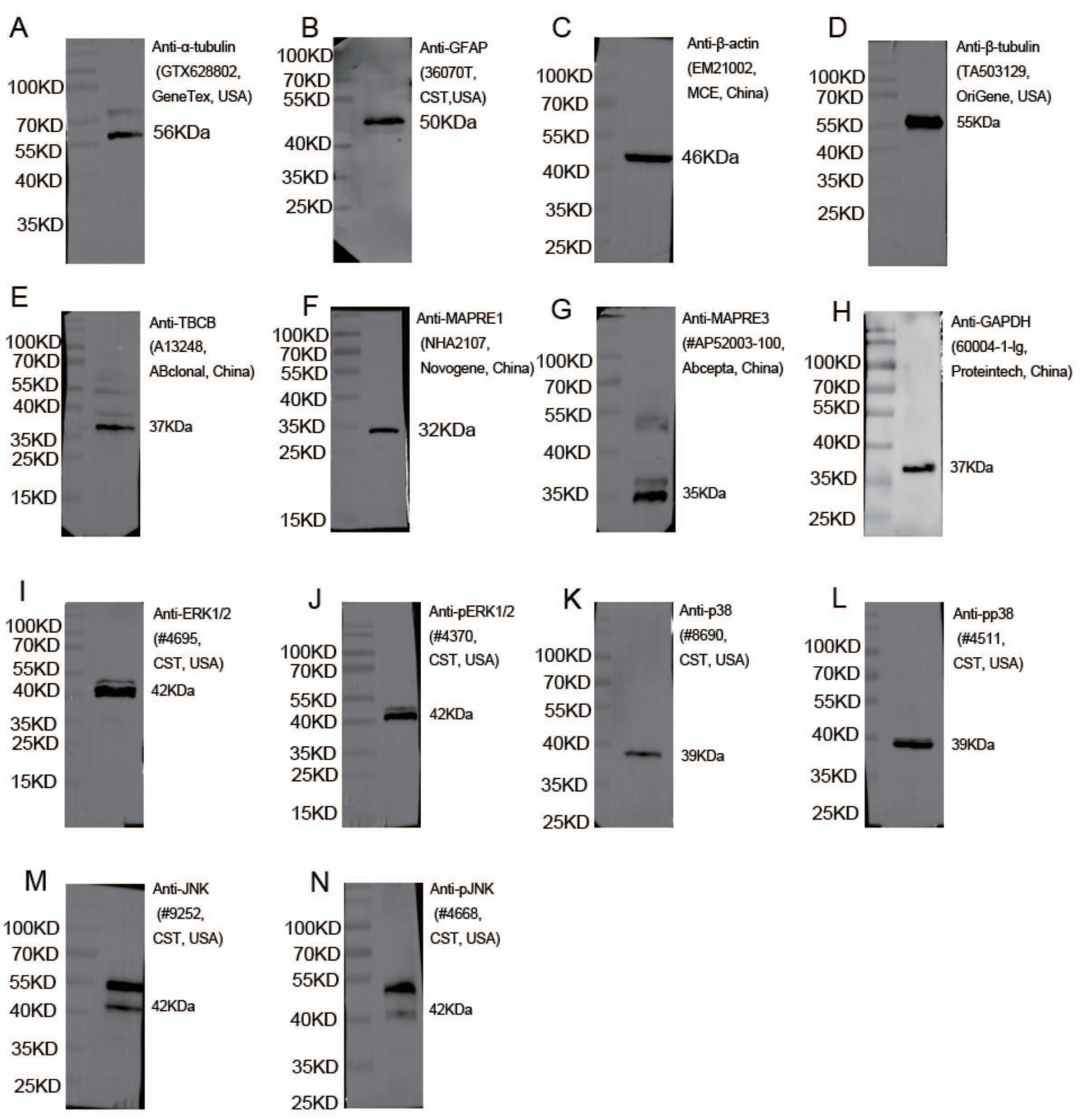

FIG2

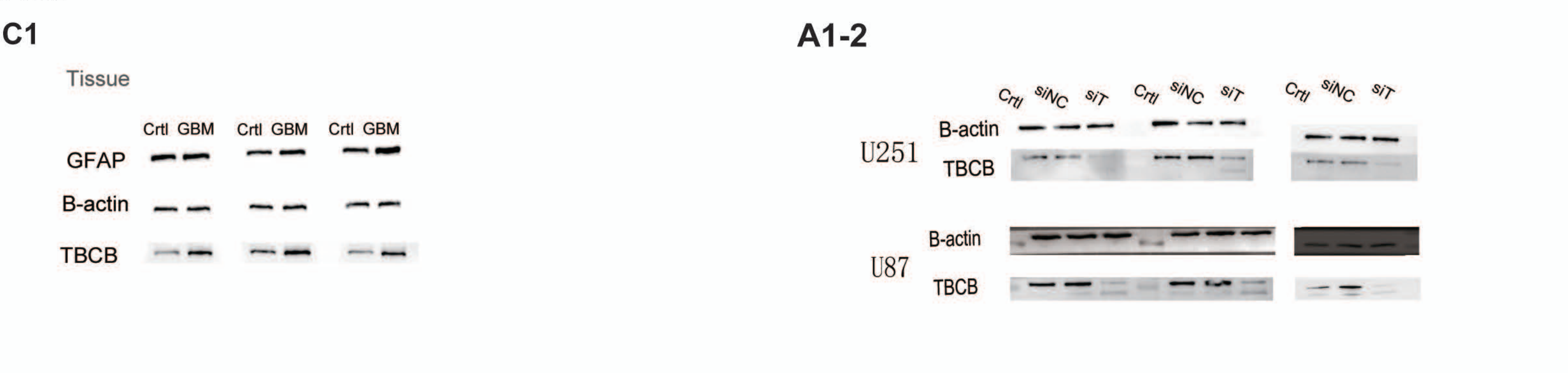

FIG5+FIG6

FIG5

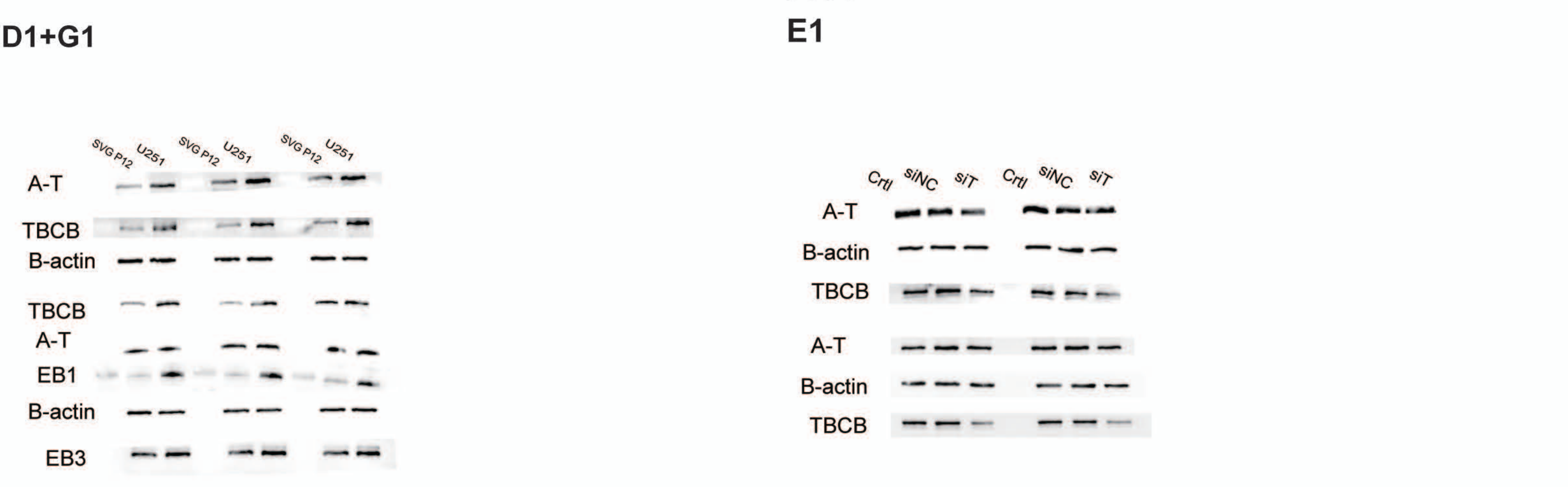

FIG6

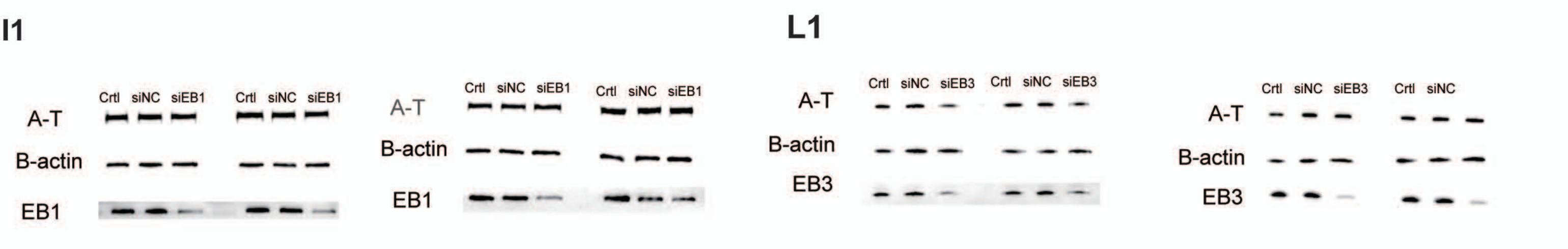

FIG7 FIG8

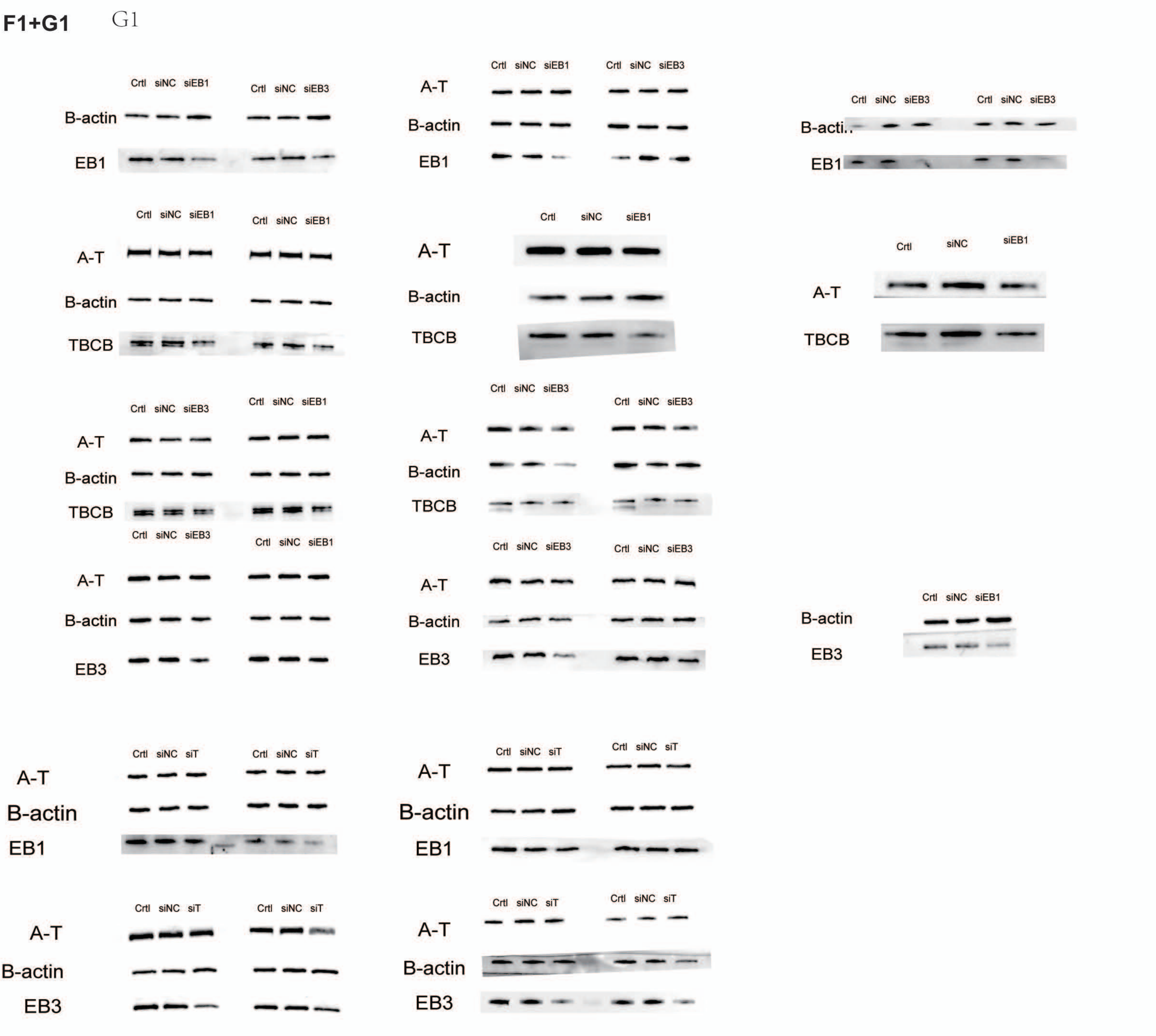

FIG8

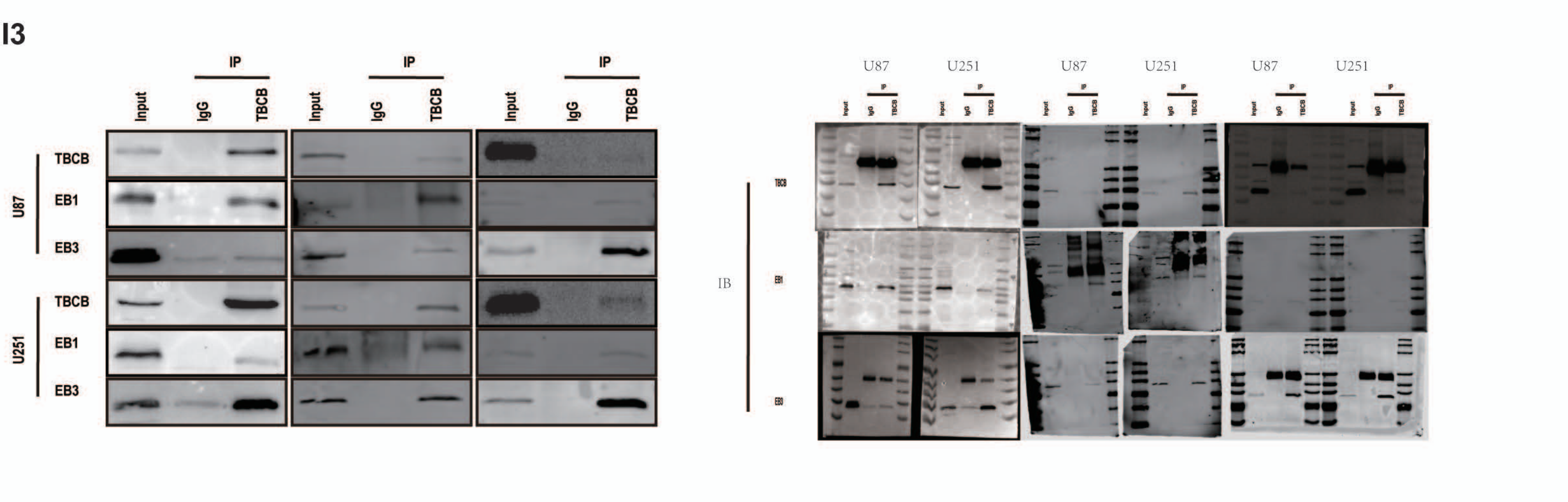

FIG S1

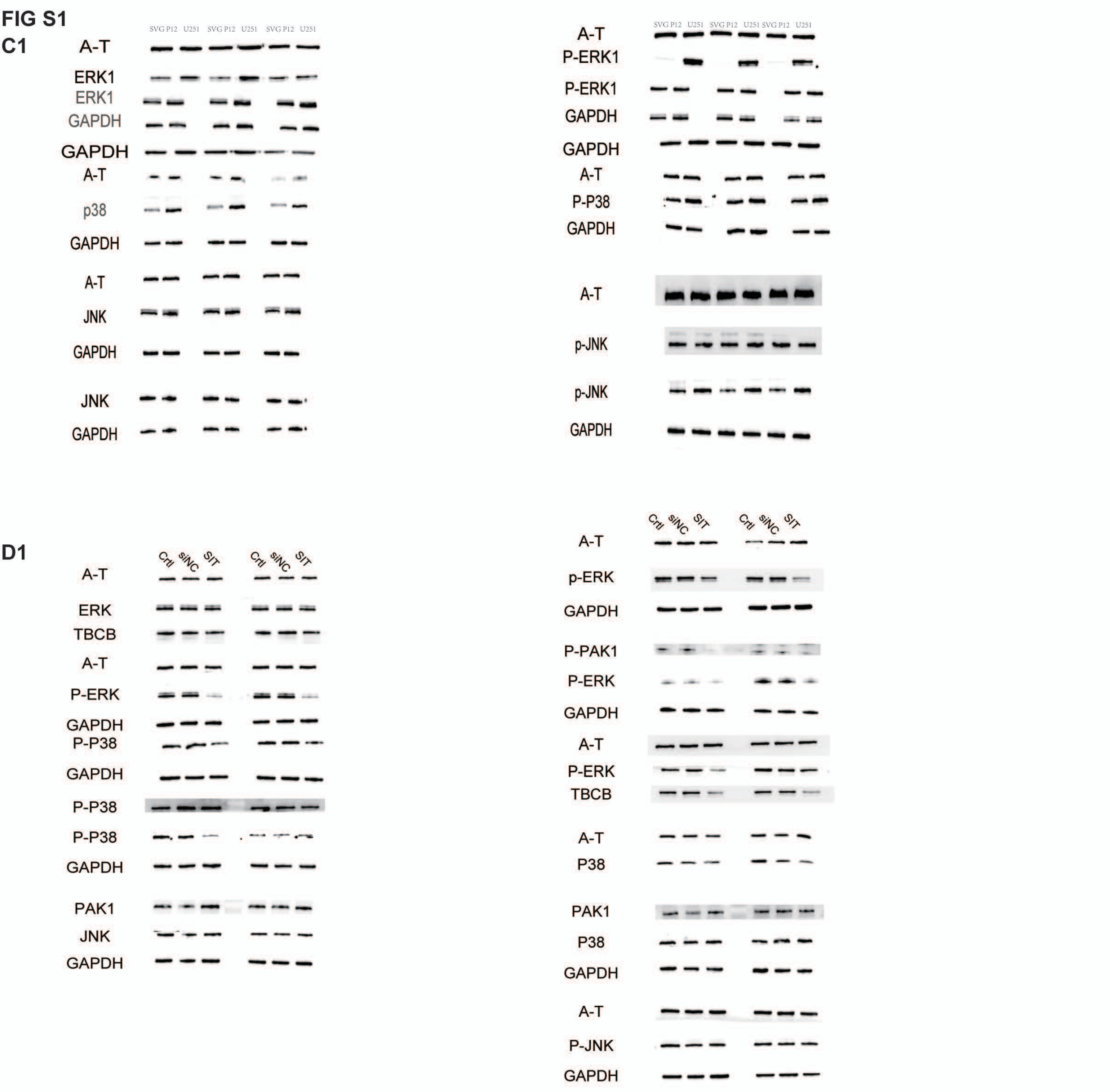

FIG S2

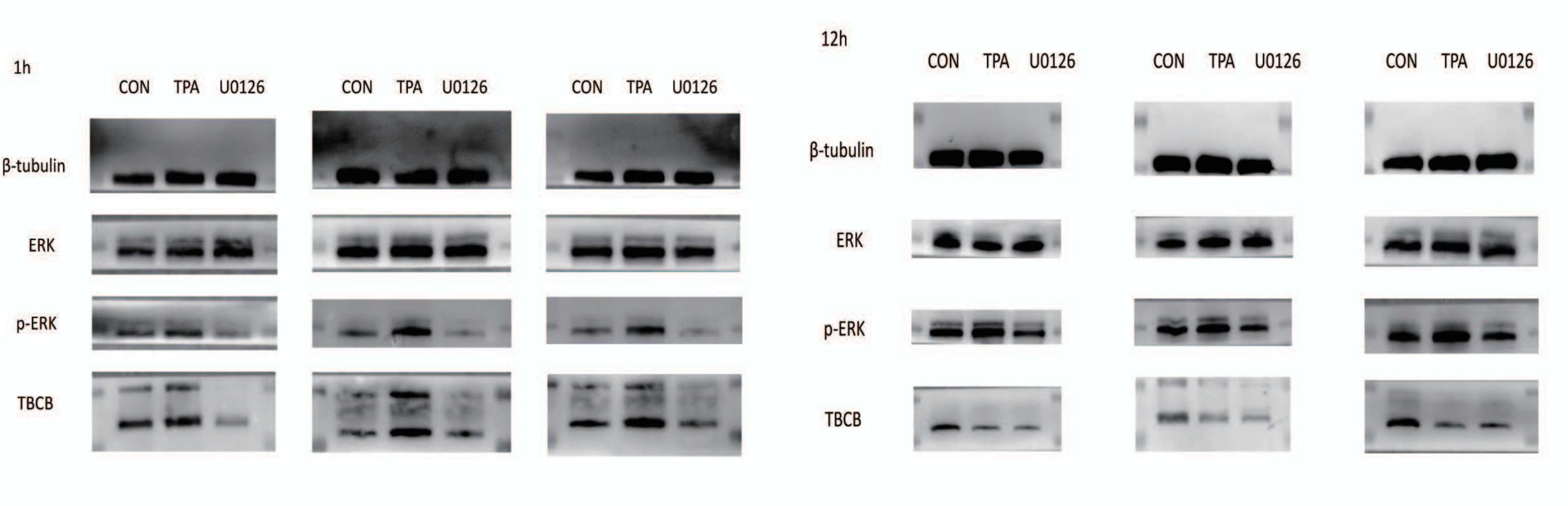

Supplement: Supplementary file 1 [file 41420_2026_3232_MOESM4_ESM.pdf]
