# Supplementary material for: The role and mechanism of tubulin folding cofactor B in glioblastoma migration
Source: Cell Death Discov. 2026 Jun 30;12:376. doi: 10.1038/s41420-026-03232-6 (PMC13586178; doi:10.1038/s41420-026-03232-6)
Supplement: Supplementary file 2 [file 41420_2026_3232_MOESM1_ESM.docx]

Supplementary Figure Legends

Supplementary Figure S1. Transcriptomic and biochemical analyses implicate MAPK/ERK signaling in TBCB-associated GBM phenotypes.

(A) Venn diagram showing the intersection of KEGG pathways significantly enriched (P < 0.05) among differentially expressed genes (DEGs) in U87 and U251 cells after TBCB knockdown (siTBCB). A total of 36 KEGG pathways are commonly enriched in both cell lines. (B) Bar plot of the top 10 common KEGG pathways ranked by gene count derived from the 36 shared pathways in (A). The MAPK signaling pathway (highlighted in red) is significantly enriched in both cell lines and was selected as a candidate pathway for further functional validation. (C) Western blot analysis of the MAPK (ERK, p38, JNK) signaling pathway in normal astrocytes (SVG P12) and glioblastoma cells (U251), showing phosphorylation levels and relative ratios of p-ERK/ERK, p-p38/p38 and p-JNK/JNK. The p-ERK/ERK, p-p38/p38 and p-JNK/JNK ratios are increased in U251 cells compared with SVG P12 cells (P < 0.05). (D1) Representative Western blots of TBCB, ERK, p-ERK, p38, p-p38, JNK, p-JNK, and β-tubulin in U251-Ctrl, U251-siNC, and U251-siTBCB cells. (D2) Quantification of TBCB protein levels. (D3) Quantification of the p-ERK/ERK ratio. (D4) Quantification of the p-p38/p38 ratio. (D5) Quantification of the p-JNK/JNK ratio. The p-ERK/ERK ratio is decreased in the siTBCB group compared with the U251-siNC group, whereas the p-p38/p38 and p-JNK/JNK ratios show no statistically significant changes. Data are presented as mean ± SD (n = 3 independent biological experiments for the Western blot quantification shown in C and D). *P < 0.05; ns, not significant.

Supplementary Figure S2. Time-dependent and nonlinear regulation of TBCB by ERK-pathway modulation in U251 cells. (A–C) Representative immunofluorescence images showing TBCB (green) and α-tubulin (red) in U251 cells treated with vehicle (Vehicle, A), the ERK activator TPA (B), or the MEK1/2 inhibitor U0126 (C). Cells were treated for 1 h, followed by medium replacement, and fixed at the delayed 12-h endpoint. These panels are included as descriptive context for delayed-endpoint morphology and TBCB/α-tubulin distribution.

(D1) Representative Western blots of ERK, p-ERK, TBCB, and β-tubulin in U251 cells collected immediately after 1 h treatment.

(D2–D3) Quantification of the p-ERK/ERK ratio and TBCB protein level at the 1-h endpoint.

(D4–D5) RT-qPCR analysis of DUSP6 and TBCB mRNA at the 1-h endpoint.

(E1) Representative Western blots of ERK, p-ERK, TBCB, and β-tubulin in U251 cells collected at the delayed 12-h endpoint after a 1-h drug pulse and medium replacement.

(E2–E3) Quantification of the p-ERK/ERK ratio and TBCB protein level at the delayed 12-h endpoint.

(E4–E5) RT-qPCR analysis of DUSP6 and TBCB mRNA at the delayed 12-h endpoint.

At 1 h, TPA increased p-ERK/ERK and DUSP6, whereas U0126 decreased both readouts, whereas TBCB showed modest and non-convergent early changes rather than the convergent downregulation observed at the delayed 12-h endpoint. At the delayed 12-h endpoint, however, both TPA and U0126 reduced TBCB protein and TBCB mRNA despite persistent divergence in ERK-pathway readouts. These data support a time-dependent and nonlinear ERK–TBCB relationship in GBM cells. Quantitative Western blotting and RT-qPCR analyses were performed with n = 3 independent biological experiments unless otherwise indicated. Data are presented as mean ± SD. Statistical comparisons were performed separately at each time point using one-way ANOVA followed by Dunnett's multiple-comparisons test. *P < 0.05, **P < 0.01, ***P < 0.001, ****P < 0.0001; ns, not significant.
