# Supplementary material for: The role and mechanism of tubulin folding cofactor B in glioblastoma migration
Source: Cell Death Discov. 2026 Jun 30;12:376. doi: 10.1038/s41420-026-03232-6 (PMC13586178; doi:10.1038/s41420-026-03232-6)
Supplement: Supplementary file 3 [file 41420_2026_3232_MOESM5_ESM.docx]

**Supplementary Tables**

**Supplementary Table S1. List of siRNA sequences for this study.**

| **Gene symbol** | **Target sequence** |
| --- | --- |
| siTBCB | forward GCAUGGAACUGGAGCUGUATT |
| siTBCB | reverse UGGUCAAUGACAUGG AUGCTT |
| siEB1 | forward GCAGAGGAUUGUAGAUAUUTT |
| siEB1 | reverse AAUAUCUACAAUCCUCUGCTT |
| siEB3 | forward GACGCAAACUAUGAUGGAATT |
| siEB3 | reverse UUCCAUCAUAGUUUGCGUCTT |
| Negative control siRNA | forward UUCUCCGAACGUGUCACGUTT |
| Negative control siRNA | reverse ACGUGACACGUUCGGAGAATT |

**Supplementary Table S2. List of targeted sequences for this study.**

| **Gene symbol** | **lentiviral vectors** | **Target sequence** |
| --- | --- | --- |
| shTBCB | LV3(H1/GFP&Puro) | 5’-GCATCCATGTCATTGACCATT-3’ |
| shNC | LV3(H1/GFP&Puro) | 5’-TTCTCCGAACGTGTCACGT-3’ |

**Supplementary Table S3. List of antibodies used for immunofluorescence and western blot.**

| **Antibodie** | **Experiment** | **Source** |
| --- | --- | --- |
| Anti-TBCB Anti-TBCB | 1:500 for WB and 1:50 for IF 1:80 for IP | Rabbit, A13248, ABclonal, China Rabbit, ab237483, ABcam, USA |
| Anti-MAPRE1 | 1:2000 for WB and 1:100 for IF | Rat, NHA2107, Novogene, China |
| Anti-MAPRE3 | 1:2000 for WB and 1:100 for IF | Rabbit, #AP52003-100, Abcepta, China |
| Anti-ERK1/2 | 1:1000 for WB | Rabbit, #4695, Cell Signaling Technology, USA |
| Anti-pERK1/2 | 1:1000 for WB | Rabbit, #4370, Cell Signaling Technology, USA |
| Anti-p38 | 1:1000 for WB | Rabbit, #8690, Cell Signaling Technology, USA |
| Anti-pp38 | 1:800 for WB | Rabbit, #4511, Cell Signaling Technology, USA |
| Anti-JNK | 1:1000 for WB | Rabbit, #9252, Cell Signaling Technology, USA |
| Anti-p-JNK | 1:800 for WB | Rabbit, #4668, Cell Signaling Technology, USA |
| Anti-β-actin | 1:5000 for WB | Mouse, EM21002, MCE, China |
| Anti-GFAP | 1:5000 for WB and 1:500 for IF | Mouse, 36070T, Cell Signaling Technology, USA |
| Anti-α-T | 1:5000 for WB and 1:400 for IF | Mouse, GTX628802, GeneTex, USA |
| Anti-β-T | 1:5000 for WB | Mouse, TA503129, OriGene, USA |

**Supplementary Table S4. List of primers used for real-time PCR.**

| **Gene symbol** | **Forward primer** | **Reverse primer** |
| --- | --- | --- |
| TBCB | GAGAAGCGATACAGCCGCA | TACAGGGTAGGAGCCCAGGA |
| EB1 | TGCCGAGAGCCGAAGA | CATAAACTGACAATACGCAGCC |
| EB3 | CCACAGAGGAAGGATTCGCA | CCGACCGTGTTAGGAAAGGA |
| β-actin | TGGCACCCAGCACAATGAA | CTAAGTCATAGTCCGCCTAGA |
| DUSP6 | TTGGAGGAATTCGGCATCAAGTA | TGTACCAAGACACCACAGTTCTT |

**Supplementary Table S5. Docking scores and evaluation metrics of the top 10 ranked TBCB–EB1 and TBCB–EB3 docking models predicted by HDOCK.**

**Note.** Models were ranked according to docking score. Confidence score and ligand RMSD (Å) are reported as provided by the HDOCK output summary pages.

**Supplementary Table S5A. TBCB–EB1 docking models**

| **Rank** | **Docking score** | **Confidence score** | **Ligand RMSD (Å)** |
| --- | --- | --- | --- |
| 1 | -272.06 | 0.9199 | 36.87 |
| 2 | -202.70 | 0.7415 | 29.52 |
| 3 | -200.05 | 0.7313 | 56.44 |
| 4 | -198.36 | 0.7246 | 53.50 |
| 5 | -194.55 | 0.7091 | 43.01 |
| 6 | -194.38 | 0.7084 | 22.80 |
| 7 | -194.35 | 0.7083 | 38.79 |
| 8 | -192.14 | 0.6991 | 38.83 |
| 9 | -191.77 | 0.6975 | 41.49 |
| 10 | -190.95 | 0.6940 | 29.55 |

**Supplementary Table S5B. TBCB–EB3 docking models**

| **Rank** | **Docking score** | **Confidence score** | **Ligand RMSD (Å)** |
| --- | --- | --- | --- |
| 1 | -260.05 | 0.9003 | 54.35 |
| 2 | -258.13 | 0.8968 | 55.21 |
| 3 | -234.63 | 0.8446 | 50.37 |
| 4 | -230.52 | 0.8335 | 52.71 |
| 5 | -229.21 | 0.8298 | 61.73 |
| 6 | -209.69 | 0.7674 | 44.43 |
| 7 | -208.79 | 0.7642 | 60.32 |
| 8 | -206.72 | 0.7566 | 45.46 |
| 9 | -206.22 | 0.7548 | 51.20 |
| 10 | -203.23 | 0.7436 | 49.00 |

**Supplementary Table S6. Full list of the 761 TBCB-related DEGs used for downstream analysis.**

Columns report gene identifiers, gene names, log2 fold changes and P values for the two TBCB-related comparisons, together with whether the gene was significant in shNC vs Ctrl and whether it was included in the final 761-gene set.

| Gene symbol | Gene name | shTBCB vs Ctrl_log2FC | shTBCB vs Ctrl_Pval | shTBCB vs shNC_log2FC | shTBCB vs shNC_Pval | shNC vs Ctrl_significant? | Included in 761-gene set? |
| --- | --- | --- | --- | --- | --- | --- | --- |
| ENSMUSG00000000244 | Tspan32 | -0.6219 | 0.001914 | -0.6092 | 0.0001241 | No | Yes |
| ENSMUSG00000000318 | Clec10a | -2.353 | 0.03615 | -3.058 | 3.585e-09 | No | Yes |
| ENSMUSG00000000600 | Krit1 | 0.3552 | 0.004924 | 0.3591 | 2.777e-06 | No | Yes |
| ENSMUSG00000000690 | Hoxb6 | 0.4665 | 0.01459 | 0.7827 | 1.633e-08 | No | Yes |
| ENSMUSG00000000823 | Zfp512b | 0.3492 | 0.008009 | 0.5647 | 1.852e-08 | No | Yes |
| ENSMUSG00000000901 | Mmp11 | -0.907 | 1.907e-11 | -0.6433 | 3.503e-07 | No | Yes |
| ENSMUSG00000000903 | Vpreb3 | -1.07 | 0.004061 | -1.007 | 0.005393 | No | Yes |
| ENSMUSG00000000957 | Mmp14 | -0.6406 | 5.3e-06 | -0.656 | 5.782e-12 | No | Yes |
| ENSMUSG00000001227 | Sema6b | -0.3563 | 0.0318 | -0.5838 | 3.077e-06 | No | Yes |
| ENSMUSG00000001507 | Itga3 | -2.004 | 0.0005353 | -1.855 | 6.418e-10 | No | Yes |
| ENSMUSG00000001576 | Ergic1 | -0.3995 | 0.0002826 | -0.5456 | 1.466e-08 | No | Yes |
| ENSMUSG00000001642 | Akr1b3 | -0.4296 | 0.001744 | -0.3968 | 7.457e-07 | No | Yes |
| ENSMUSG00000001751 | Naglu | -0.954 | 1.372e-13 | -0.8775 | 8.521e-15 | No | Yes |
| ENSMUSG00000001768 | Rin2 | -0.7615 | 0.0451 | -0.6292 | 4.345e-07 | No | Yes |
| ENSMUSG00000001864 | Aif1l | -1.09 | 0.0004039 | -0.7378 | 2.8e-07 | No | Yes |
| ENSMUSG00000001946 | Esam | 1.6236 | 0.0401 | 2.1084 | 0.01183 | No | Yes |
| ENSMUSG00000001998 | Ap4e1 | 0.3423 | 0.04378 | 0.513 | 3.077e-08 | No | Yes |
| ENSMUSG00000002059 | Rab34 | 1.9289 | 0.000557 | 1.7157 | 0.0009706 | No | Yes |
| ENSMUSG00000002108 | Nr1h3 | -0.4511 | 0.03402 | -0.4165 | 0.01068 | No | Yes |
| ENSMUSG00000002233 | Rhoc | -0.2924 | 0.02289 | -0.4868 | 6.415e-09 | No | Yes |
| ENSMUSG00000002341 | Ncan | 2.2528 | 0.0409 | 3.9121 | 0.001488 | No | Yes |
| ENSMUSG00000002603 | Tgfb1 | -0.7964 | 0.0003551 | -0.903 | 4.462e-17 | No | Yes |
| ENSMUSG00000002831 | Plin4 | -0.6997 | 0.03785 | -0.5399 | 0.00035 | No | Yes |
| ENSMUSG00000002885 | Adgre5 | -0.7961 | 8.184e-07 | -0.8139 | 1.679e-10 | No | Yes |
| ENSMUSG00000002985 | Apoe | 0.7921 | 0.0003512 | 1.1148 | 3.682e-07 | No | Yes |
| ENSMUSG00000002997 | Prkar2b | -0.7618 | 0.0007159 | -0.971 | 2.989e-11 | No | Yes |
| ENSMUSG00000003053 | Cyp2c29 | 2.7596 | 0.04824 | 4.748 | 0.007678 | No | Yes |
| ENSMUSG00000003119 | Cdk12 | 0.332 | 0.005863 | 0.3284 | 0.0001375 | No | Yes |
| ENSMUSG00000003199 | Mpnd | -0.3121 | 0.01824 | -0.4044 | 0.0001782 | No | Yes |
| ENSMUSG00000003355 | Fkbp11 | -0.9306 | 0.02489 | -1.454 | 2.187e-08 | No | Yes |
| ENSMUSG00000003411 | Rab3b | 5.5125 | 8.722e-05 | 5.4863 | 6.743e-05 | No | Yes |
| ENSMUSG00000003541 | Ier3 | -0.767 | 0.002994 | -0.8476 | 8.586e-06 | No | Yes |
| ENSMUSG00000003814 | Calr | -0.4969 | 0.002771 | -0.5682 | 1.393e-11 | No | Yes |
| ENSMUSG00000003929 | Zfp81 | 0.3346 | 0.006393 | 0.4128 | 4.825e-06 | No | Yes |
| ENSMUSG00000004105 | Angptl2 | -1.154 | 0.0001571 | -1.246 | 6.726e-17 | No | Yes |
| ENSMUSG00000004371 | Il11 | 0.9475 | 0.0009591 | 1.3656 | 1.551e-07 | No | Yes |
| ENSMUSG00000004561 | Mettl17 | 0.5928 | 0.001079 | 0.4779 | 3.927e-07 | No | Yes |
| ENSMUSG00000004565 | Pnpla6 | 0.4067 | 0.02334 | 0.7483 | 2.491e-10 | No | Yes |
| ENSMUSG00000004626 | Stxbp2 | 0.84 | 0.01721 | 1.1932 | 0.0008502 | No | Yes |
| ENSMUSG00000004988 | Fxyd4 | -1.597 | 0.0249 | -1.49 | 0.02189 | No | Yes |
| ENSMUSG00000005043 | Sgsh | -0.5808 | 0.0001658 | -0.4864 | 9.078e-05 | No | Yes |
| ENSMUSG00000005045 | Chd5 | -0.538 | 0.01469 | -0.527 | 0.004783 | No | Yes |
| ENSMUSG00000005534 | Insr | -0.3717 | 0.01126 | -0.4068 | 5.04e-05 | No | Yes |
| ENSMUSG00000005575 | Ube2m | -0.3503 | 0.00457 | -0.4116 | 7.401e-06 | No | Yes |
| ENSMUSG00000005628 | Tmod4 | 0.8826 | 0.005373 | 0.7556 | 0.0009589 | No | Yes |
| ENSMUSG00000005897 | Nr2c1 | -0.4128 | 0.02152 | -0.3811 | 0.007172 | No | Yes |
| ENSMUSG00000005899 | Smpd4 | 0.3591 | 0.01085 | 0.3037 | 0.003536 | No | Yes |
| ENSMUSG00000006095 | Tbcb | -2.54 | 3.418e-39 | -2.701 | 1.376e-95 | No | Yes |
| ENSMUSG00000006301 | Tmbim1 | -0.4679 | 0.0006869 | -0.6042 | 4.168e-09 | No | Yes |
| ENSMUSG00000006313 | Upk1a | 1.174 | 0.04129 | 1.4466 | 0.03886 | No | Yes |
| ENSMUSG00000006342 | Susd2 | 1.2001 | 0.01526 | 1.7804 | 7.188e-06 | No | Yes |
| ENSMUSG00000006360 | Crip1 | -1.64 | 0.03284 | -2.094 | 0.005235 | No | Yes |
| ENSMUSG00000006457 | Actn3 | -1.48 | 0.01137 | -1.013 | 0.002197 | No | Yes |
| ENSMUSG00000006740 | Kif5b | 0.2946 | 0.04488 | 0.3189 | 0.0004058 | No | Yes |
| ENSMUSG00000006764 | Tph2 | -0.6887 | 0.001089 | -0.8589 | 2.21e-10 | No | Yes |
| ENSMUSG00000006850 | Tmco6 | -0.6402 | 0.01788 | -0.5566 | 0.009984 | No | Yes |
| ENSMUSG00000007570 | Fance | -0.43 | 0.02338 | -0.4056 | 0.01158 | No | Yes |
| ENSMUSG00000007817 | Zmiz1 | -0.4718 | 0.003767 | -0.491 | 8.983e-06 | No | Yes |
| ENSMUSG00000007946 | Phox2a | 2.993 | 0.02318 | 4.9793 | 0.002931 | No | Yes |
| ENSMUSG00000008540 | Mgst1 | -0.7778 | 0.01041 | -0.5327 | 4.249e-06 | No | Yes |
| ENSMUSG00000008855 | Hdac5 | -0.6834 | 0.01895 | -0.3377 | 0.02169 | No | Yes |
| ENSMUSG00000009185 | Ccl8 | -3.698 | 0.01117 | -3.472 | 0.006666 | No | Yes |
| ENSMUSG00000009394 | Syn2 | -1.356 | 0.003563 | -0.8741 | 0.0009879 | No | Yes |
| ENSMUSG00000009731 | Kcnd1 | 1.5526 | 0.002833 | 1.8691 | 1.036e-06 | No | Yes |
| ENSMUSG00000009876 | Cox4i2 | -3.219 | 0.0006921 | -2.74 | 0.0006587 | No | Yes |
| ENSMUSG00000010097 | Nxf1 | 0.6712 | 6.928e-09 | 0.4356 | 2.915e-07 | No | Yes |
| ENSMUSG00000010307 | Tmem86a | -0.8584 | 0.0002723 | -0.656 | 6.468e-05 | No | Yes |
| ENSMUSG00000012640 | Zfp715 | 0.4053 | 0.0008688 | 0.3713 | 2.301e-05 | No | Yes |
| ENSMUSG00000013495 | Tmem175 | -0.4907 | 0.01391 | -0.7353 | 6.902e-08 | No | Yes |
| ENSMUSG00000014606 | Slc25a11 | -0.5076 | 2.404e-05 | -0.6278 | 1.807e-13 | No | Yes |
| ENSMUSG00000014686 | Ceacam16 | -1.037 | 0.006183 | -1.662 | 2.526e-07 | No | Yes |
| ENSMUSG00000015027 | Galns | -0.652 | 0.0005351 | -0.6637 | 1.176e-06 | No | Yes |
| ENSMUSG00000015243 | Abca1 | -0.8983 | 0.008704 | -0.6244 | 1.081e-09 | No | Yes |
| ENSMUSG00000015291 | Gdi1 | 0.3977 | 0.01266 | 0.412 | 0.0001948 | No | Yes |
| ENSMUSG00000015354 | Pcolce2 | -0.5756 | 0.005221 | -0.9409 | 3.043e-08 | No | Yes |
| ENSMUSG00000015377 | Dennd6b | 0.5379 | 0.03907 | 0.3138 | 0.01355 | No | Yes |
| ENSMUSG00000015461 | Atf6b | -0.4271 | 0.00118 | -0.2702 | 0.01003 | No | Yes |
| ENSMUSG00000015711 | Prune1 | -0.6369 | 2.224e-05 | -0.4689 | 9.013e-06 | No | Yes |
| ENSMUSG00000015968 | Cacna1d | -1.54 | 0.01264 | -1.637 | 0.01935 | No | Yes |
| ENSMUSG00000016018 | Mtrex | 0.3147 | 0.01397 | 0.3431 | 0.0002233 | No | Yes |
| ENSMUSG00000016024 | Lbp | -0.6718 | 0.0003992 | -0.7635 | 5.447e-06 | No | Yes |
| ENSMUSG00000016194 | Hsd11b1 | -1.914 | 3.369e-05 | -1.074 | 0.02918 | No | Yes |
| ENSMUSG00000016200 | Syt14 | 2.5877 | 0.01437 | 2.7498 | 0.01683 | No | Yes |
| ENSMUSG00000016552 | Foxred2 | -0.3673 | 0.009615 | -0.3474 | 0.0002093 | No | Yes |
| ENSMUSG00000017188 | Coa3 | -0.5489 | 0.001866 | -0.5331 | 0.0001507 | No | Yes |
| ENSMUSG00000017697 | Ada | -0.49 | 0.04797 | -0.907 | 2.247e-06 | No | Yes |
| ENSMUSG00000017754 | Pltp | -0.6943 | 0.001251 | -0.2924 | 0.007904 | No | Yes |
| ENSMUSG00000017929 | B4galt5 | -0.9663 | 0.0004299 | -0.4872 | 3.869e-06 | No | Yes |
| ENSMUSG00000018543 | 1700001P01Rik | 1.5754 | 0.0003562 | 1.0885 | 0.007796 | No | Yes |
| ENSMUSG00000018593 | Sparc | -0.4696 | 0.00272 | -0.4978 | 0.006443 | No | Yes |
| ENSMUSG00000018669 | Cdk5rap3 | -0.4414 | 0.03596 | -0.5256 | 0.001656 | No | Yes |
| ENSMUSG00000018819 | Lsp1 | -0.6505 | 0.03249 | -1.14 | 7.549e-06 | No | Yes |
| ENSMUSG00000018849 | Wwc1 | -1.358 | 0.000412 | -0.8816 | 0.04742 | No | Yes |
| ENSMUSG00000018906 | P4ha2 | -0.5714 | 0.01727 | -0.368 | 0.0143 | No | Yes |
| ENSMUSG00000018924 | Alox15 | 2.8018 | 0.004751 | 3.5672 | 0.0003061 | No | Yes |
| ENSMUSG00000019122 | Ccl9 | -0.8779 | 0.00669 | -1.33 | 8.489e-18 | No | Yes |
| ENSMUSG00000019143 | Hars2 | 0.3564 | 0.007049 | 0.3039 | 0.001237 | No | Yes |
| ENSMUSG00000019312 | Grb7 | 1.1796 | 0.02825 | 2.078 | 0.0007417 | No | Yes |
| ENSMUSG00000019590 | Cyb561 | -1.148 | 0.000852 | -0.9611 | 3.283e-07 | No | Yes |
| ENSMUSG00000019767 | Ccdc170 | -2.287 | 0.0007866 | -1.548 | 0.02618 | No | Yes |
| ENSMUSG00000019791 | Hint3 | 0.4856 | 0.0249 | 0.298 | 0.04419 | No | Yes |
| ENSMUSG00000019841 | Rev3l | 0.4508 | 0.001375 | 0.3684 | 7.439e-06 | No | Yes |
| ENSMUSG00000019966 | Kitl | -0.8151 | 0.0003978 | -0.4665 | 3.55e-07 | No | Yes |
| ENSMUSG00000019987 | Arg1 | 1.344 | 0.02224 | 2.5343 | 0.0003643 | No | Yes |
| ENSMUSG00000020017 | Hal | 1.537 | 0.01034 | 1.4765 | 0.01465 | No | Yes |
| ENSMUSG00000020096 | Tbata | -5.001 | 0.002047 | -4.379 | 0.03401 | No | Yes |
| ENSMUSG00000020216 | Jsrp1 | 1.5533 | 0.0006659 | 1.936 | 5.386e-08 | No | Yes |
| ENSMUSG00000020246 | Hcfc2 | 0.3678 | 0.001227 | 0.4821 | 1.988e-08 | No | Yes |
| ENSMUSG00000020288 | Ahsa2 | 0.7226 | 0.0005332 | 0.5558 | 0.001141 | No | Yes |
| ENSMUSG00000020372 | Rack1 | -0.2747 | 0.01636 | -0.3416 | 5.849e-05 | No | Yes |
| ENSMUSG00000020400 | Tnip1 | -0.5353 | 0.03542 | -0.634 | 0.0004014 | No | Yes |
| ENSMUSG00000020424 | Castor1 | -0.7353 | 0.001827 | -0.4866 | 0.01126 | No | Yes |
| ENSMUSG00000020447 | Npc1l1 | 0.6811 | 0.04798 | 1.4211 | 3.279e-05 | No | Yes |
| ENSMUSG00000020463 | Ppp4r3b | 0.3086 | 0.00894 | 0.3313 | 9.211e-05 | No | Yes |
| ENSMUSG00000020484 | Xbp1 | -0.427 | 0.001368 | -0.3109 | 0.003518 | No | Yes |
| ENSMUSG00000020486 | Septin4 | -0.7163 | 0.01688 | -0.7487 | 0.004952 | No | Yes |
| ENSMUSG00000020592 | Sdc1 | -0.6981 | 0.0009475 | -0.6474 | 1.502e-08 | No | Yes |
| ENSMUSG00000020600 | Slc7a15 | 0.7425 | 0.01779 | 1.0028 | 0.0002856 | No | Yes |
| ENSMUSG00000020695 | Mrc2 | -0.3862 | 0.04419 | -0.6321 | 2.054e-10 | No | Yes |
| ENSMUSG00000020776 | Fbf1 | -0.3683 | 0.02455 | -0.4141 | 0.001221 | No | Yes |
| ENSMUSG00000020792 | Exoc7 | -0.4572 | 0.007462 | -0.3563 | 0.005741 | No | Yes |
| ENSMUSG00000020839 | Tmigd1 | -0.9661 | 0.01905 | -0.4369 | 0.01543 | No | Yes |
| ENSMUSG00000020848 | Doc2b | 0.756 | 0.01115 | 1.1581 | 7.234e-05 | No | Yes |
| ENSMUSG00000020865 | Abcc3 | -1.685 | 0.004254 | -1.236 | 0.0004436 | No | Yes |
| ENSMUSG00000020900 | Myh10 | 0.7222 | 0.02773 | 0.8217 | 0.0005234 | No | Yes |
| ENSMUSG00000020901 | Pik3r5 | -1.261 | 0.005683 | -1.858 | 1.422e-12 | No | Yes |
| ENSMUSG00000020908 | Myh3 | 1.0132 | 0.03403 | 1.4401 | 0.0009839 | No | Yes |
| ENSMUSG00000020932 | Gfap | 0.8767 | 0.03892 | 1.4117 | 0.0008282 | No | Yes |
| ENSMUSG00000020945 | Lyzl6 | 5.3664 | 0.0009581 | 3.2656 | 0.01248 | No | Yes |
| ENSMUSG00000020982 | Nemf | 0.4041 | 0.0006188 | 0.3376 | 3.28e-05 | No | Yes |
| ENSMUSG00000020994 | Pnn | 0.6891 | 0.0006393 | 0.376 | 8.868e-06 | No | Yes |
| ENSMUSG00000021047 | Nova1 | 0.5211 | 0.0001138 | 0.551 | 1.687e-06 | No | Yes |
| ENSMUSG00000021108 | Prkch | -1.177 | 0.01821 | -1.566 | 8.033e-09 | No | Yes |
| ENSMUSG00000021130 | Galnt16 | 2.2888 | 0.00158 | 3.1563 | 8.845e-05 | No | Yes |
| ENSMUSG00000021281 | Tnfaip2 | -0.6284 | 0.0006164 | -0.8231 | 3.816e-13 | No | Yes |
| ENSMUSG00000021302 | Ggps1 | 0.2952 | 0.0147 | 0.4791 | 1.743e-06 | No | Yes |
| ENSMUSG00000021390 | Ogn | 1.2832 | 0.001934 | 1.1958 | 5.205e-08 | No | Yes |
| ENSMUSG00000021404 | Serpinb9c | -3.061 | 3.551e-05 | -3.044 | 7.079e-07 | No | Yes |
| ENSMUSG00000021482 | Prxl2c | 0.31 | 0.03059 | 0.4915 | 0.000353 | No | Yes |
| ENSMUSG00000021500 | Ddx46 | 0.3214 | 0.003188 | 0.2675 | 0.001226 | No | Yes |
| ENSMUSG00000021510 | Zfp729a | 0.3442 | 0.03295 | 0.2743 | 0.001444 | No | Yes |
| ENSMUSG00000021738 | Atxn7 | 0.4308 | 0.000341 | 0.4686 | 2.983e-07 | No | Yes |
| ENSMUSG00000021752 | Kctd6 | 0.5001 | 0.0009578 | 0.5596 | 7.053e-07 | No | Yes |
| ENSMUSG00000021815 | Mss51 | 0.9306 | 0.000868 | 0.9434 | 0.0008112 | No | Yes |
| ENSMUSG00000021824 | Ap3m1 | 0.2636 | 0.04108 | 0.3067 | 0.0002138 | No | Yes |
| ENSMUSG00000021876 | Rnase4 | -1.274 | 0.005856 | -0.5565 | 0.01013 | No | Yes |
| ENSMUSG00000021902 | Phf7 | -0.4834 | 0.02618 | -0.3999 | 0.01667 | No | Yes |
| ENSMUSG00000021904 | Sema3g | -0.6098 | 0.03849 | -0.661 | 0.002665 | No | Yes |
| ENSMUSG00000021913 | Ogdhl | -0.3841 | 0.002553 | -0.389 | 0.0002673 | No | Yes |
| ENSMUSG00000021945 | Zmym2 | 0.3625 | 0.001359 | 0.4369 | 3.39e-07 | No | Yes |
| ENSMUSG00000021972 | Hmbox1 | 0.3826 | 0.002402 | 0.4338 | 1.724e-05 | No | Yes |
| ENSMUSG00000022144 | Gdnf | -1.595 | 0.003267 | -1.622 | 0.0005877 | No | Yes |
| ENSMUSG00000022176 | Rem2 | 2.9124 | 3.46e-05 | 1.4328 | 0.008689 | No | Yes |
| ENSMUSG00000022185 | Acin1 | 0.2949 | 0.005767 | 0.3607 | 7.812e-06 | No | Yes |
| ENSMUSG00000022205 | Sub1 | 0.3602 | 0.001689 | 0.2747 | 0.001139 | No | Yes |
| ENSMUSG00000022217 | Emc9 | 2.4315 | 0.02233 | 3.1951 | 0.002048 | No | Yes |
| ENSMUSG00000022261 | Sdc2 | 0.4002 | 0.0002283 | 0.3994 | 1.103e-06 | No | Yes |
| ENSMUSG00000022357 | Klhl38 | -1.686 | 0.0014 | -2.22 | 9.589e-07 | No | Yes |
| ENSMUSG00000022438 | Parvb | -0.5909 | 0.0001203 | -0.3188 | 0.01952 | No | Yes |
| ENSMUSG00000022476 | Polr3h | -0.3158 | 0.025 | -0.5712 | 1.352e-07 | No | Yes |
| ENSMUSG00000022518 | 4930562C15Rik | 0.7401 | 7.779e-05 | 1.0382 | 6.05e-09 | No | Yes |
| ENSMUSG00000022528 | Hes1 | -0.8088 | 0.007401 | -0.4388 | 0.02977 | No | Yes |
| ENSMUSG00000022594 | Lynx1 | -0.7986 | 0.003741 | -0.5097 | 0.00183 | No | Yes |
| ENSMUSG00000022753 | Tmem30c | 2.0246 | 0.00328 | 3.9709 | 5.942e-05 | No | Yes |
| ENSMUSG00000022757 | Tfg | 0.393 | 0.0007259 | 0.4297 | 1.29e-06 | No | Yes |
| ENSMUSG00000022769 | Sdf2l1 | -0.5751 | 0.01417 | -0.5808 | 3.11e-05 | No | Yes |
| ENSMUSG00000022773 | Ypel1 | 0.3857 | 0.03186 | 0.7909 | 1.087e-05 | No | Yes |
| ENSMUSG00000022878 | Adipoq | 2.0999 | 0.04772 | 5.4321 | 0.0004367 | No | Yes |
| ENSMUSG00000022887 | Masp1 | -0.8985 | 7.044e-06 | -0.9832 | 5.208e-09 | No | Yes |
| ENSMUSG00000022895 | Ets2 | -0.3017 | 0.02646 | -0.3505 | 0.004112 | No | Yes |
| ENSMUSG00000022961 | Son | 0.4096 | 0.004128 | 0.2711 | 0.001524 | No | Yes |
| ENSMUSG00000022978 | Mis18a | 0.3157 | 0.02891 | 0.2635 | 0.01841 | No | Yes |
| ENSMUSG00000023044 | Csad | 0.483 | 0.04447 | 0.519 | 0.001979 | No | Yes |
| ENSMUSG00000023064 | Sncg | -0.9811 | 0.01296 | -0.6042 | 2.703e-05 | No | Yes |
| ENSMUSG00000023367 | Tmem176a | -1.177 | 2.363e-06 | -1.045 | 3.714e-06 | No | Yes |
| ENSMUSG00000023805 | Synj2 | -0.3737 | 0.009139 | -0.3037 | 0.008645 | No | Yes |
| ENSMUSG00000023885 | Thbs2 | -1.375 | 1.956e-07 | -1.596 | 1.969e-39 | No | Yes |
| ENSMUSG00000023992 | Trem2 | -2.094 | 0.02979 | -1.707 | 0.002367 | No | Yes |
| ENSMUSG00000024045 | Akap8 | 0.4562 | 6.461e-05 | 0.3584 | 3.922e-05 | No | Yes |
| ENSMUSG00000024053 | Emilin2 | -1.462 | 7.137e-16 | -1.399 | 1.144e-21 | No | Yes |
| ENSMUSG00000024136 | Dnase1l2 | 1.4732 | 0.01539 | 1.0614 | 0.01239 | No | Yes |
| ENSMUSG00000024164 | C3 | -1.977 | 0.005784 | -1.486 | 0.01335 | No | Yes |
| ENSMUSG00000024173 | Tpsab1 | -1.942 | 5.251e-10 | -1.794 | 6.301e-13 | No | Yes |
| ENSMUSG00000024298 | Zfp871 | 0.3885 | 0.008365 | 0.4889 | 7.159e-07 | No | Yes |
| ENSMUSG00000024369 | Nelfe | -0.38 | 0.005794 | -0.6084 | 3.662e-09 | No | Yes |
| ENSMUSG00000024395 | Lims2 | -1.865 | 7.572e-05 | -1.119 | 0.001829 | No | Yes |
| ENSMUSG00000024409 | Psors1c2 | 0.9726 | 0.01979 | 0.9966 | 0.007977 | No | Yes |
| ENSMUSG00000024426 | Atat1 | 0.4973 | 0.02519 | 0.5275 | 0.003609 | No | Yes |
| ENSMUSG00000024474 | Ik | 0.3588 | 0.004841 | 0.4098 | 1.45e-05 | No | Yes |
| ENSMUSG00000024579 | Pcyox1l | -0.5282 | 0.004713 | -0.5927 | 1.905e-05 | No | Yes |
| ENSMUSG00000024589 | Nedd4l | -0.2886 | 0.01718 | -0.31 | 0.002635 | No | Yes |
| ENSMUSG00000024646 | Cyb5a | -0.4889 | 0.03615 | -0.4085 | 5.606e-05 | No | Yes |
| ENSMUSG00000024665 | Fads2 | 1.5352 | 0.002716 | 1.2779 | 0.0006929 | No | Yes |
| ENSMUSG00000024736 | Tmem132a | -0.595 | 0.007092 | -0.7638 | 1.634e-06 | No | Yes |
| ENSMUSG00000024737 | Slc15a3 | -0.9513 | 7.659e-06 | -0.9986 | 5.469e-14 | No | Yes |
| ENSMUSG00000024780 | Cdc37l1 | 0.3394 | 0.0016 | 0.3713 | 4.178e-06 | No | Yes |
| ENSMUSG00000024824 | Rad9a | -0.5097 | 0.01811 | -0.5667 | 5.473e-05 | No | Yes |
| ENSMUSG00000024846 | Cst6 | -1.022 | 0.001157 | -0.4471 | 0.01891 | No | Yes |
| ENSMUSG00000024936 | Kcnk7 | 0.7969 | 0.03185 | 0.8732 | 0.04626 | No | Yes |
| ENSMUSG00000025013 | Tll2 | 5.7032 | 7.194e-05 | 5.679 | 4.579e-05 | No | Yes |
| ENSMUSG00000025024 | Smndc1 | 0.3491 | 0.005167 | 0.4357 | 7.073e-06 | No | Yes |
| ENSMUSG00000025044 | Msr1 | 0.776 | 0.03171 | 0.826 | 0.01117 | No | Yes |
| ENSMUSG00000025069 | Gsto2 | -1.108 | 4.755e-06 | -1.523 | 3.833e-09 | No | Yes |
| ENSMUSG00000025130 | P4hb | -0.3591 | 0.005457 | -0.3167 | 0.0004355 | No | Yes |
| ENSMUSG00000025150 | Cbr2 | -0.6128 | 0.005334 | -0.8546 | 4.689e-13 | No | Yes |
| ENSMUSG00000025185 | Loxl4 | -0.5231 | 0.03727 | -0.6429 | 0.0006633 | No | Yes |
| ENSMUSG00000025209 | Twnk | -0.3129 | 0.01673 | -0.5691 | 1.411e-07 | No | Yes |
| ENSMUSG00000025323 | Sp4 | 0.5139 | 0.01047 | 0.5664 | 7.938e-05 | No | Yes |
| ENSMUSG00000025380 | Fscn2 | 1.9636 | 0.0004645 | 1.821 | 0.0003183 | No | Yes |
| ENSMUSG00000025381 | Cnpy2 | -0.3594 | 0.009883 | -0.4428 | 0.0001964 | No | Yes |
| ENSMUSG00000025466 | Fuom | -0.6173 | 0.004055 | -0.7494 | 2.704e-06 | No | Yes |
| ENSMUSG00000025473 | Adam8 | 1.3048 | 0.001281 | 1.771 | 2.237e-06 | No | Yes |
| ENSMUSG00000025479 | Cyp2e1 | 5.0665 | 0.002056 | 5.0433 | 0.001539 | No | Yes |
| ENSMUSG00000025510 | Cd151 | -0.5591 | 0.000384 | -0.5301 | 7.148e-06 | No | Yes |
| ENSMUSG00000025512 | Chid1 | -0.6416 | 0.003059 | -0.5335 | 4.267e-05 | No | Yes |
| ENSMUSG00000025650 | Col7a1 | 0.7977 | 0.0163 | 0.5653 | 0.00143 | No | Yes |
| ENSMUSG00000025792 | Slc25a10 | -0.8049 | 0.03186 | -1.326 | 1.95e-15 | No | Yes |
| ENSMUSG00000025854 | Fam20c | -0.5245 | 0.04334 | -0.4516 | 0.0007907 | No | Yes |
| ENSMUSG00000025912 | Mybl1 | 0.3241 | 0.01834 | 0.3086 | 0.001751 | No | Yes |
| ENSMUSG00000026082 | Rev1 | 0.3629 | 0.0092 | 0.361 | 0.000623 | No | Yes |
| ENSMUSG00000026096 | Osgepl1 | 0.3726 | 0.01906 | 0.3713 | 0.0047 | No | Yes |
| ENSMUSG00000026111 | Unc50 | 0.3373 | 0.008823 | 0.3699 | 9.461e-05 | No | Yes |
| ENSMUSG00000026204 | Ptprn | 0.9457 | 0.0217 | 1.0046 | 0.01401 | No | Yes |
| ENSMUSG00000026238 | Ptma | -0.2927 | 0.01224 | -0.5112 | 2.71e-11 | No | Yes |
| ENSMUSG00000026240 | Cops7b | 0.3063 | 0.0294 | 0.3811 | 9.081e-05 | No | Yes |
| ENSMUSG00000026278 | Bok | -0.6103 | 0.0008293 | -0.4995 | 0.0004945 | No | Yes |
| ENSMUSG00000026305 | Lrrfip1 | -0.5003 | 0.01082 | -0.6903 | 9.194e-13 | No | Yes |
| ENSMUSG00000026409 | Pfkfb2 | 0.4442 | 0.01685 | 0.3564 | 0.0006866 | No | Yes |
| ENSMUSG00000026437 | Cdk18 | -0.5004 | 0.02407 | -0.3828 | 0.005564 | No | Yes |
| ENSMUSG00000026458 | Ppfia4 | 1.1411 | 0.009798 | 0.9657 | 0.01681 | No | Yes |
| ENSMUSG00000026479 | Lamc2 | -0.5535 | 0.00155 | -0.4302 | 0.0007061 | No | Yes |
| ENSMUSG00000026564 | Dusp27 | -0.5504 | 0.007541 | -0.4105 | 0.00316 | No | Yes |
| ENSMUSG00000026667 | Uhmk1 | 0.36 | 0.001428 | 0.43 | 3.211e-06 | No | Yes |
| ENSMUSG00000026688 | Mgst3 | -0.567 | 0.02709 | -0.4564 | 0.001249 | No | Yes |
| ENSMUSG00000026709 | Dars2 | -0.3269 | 0.03113 | -0.5719 | 3.635e-06 | No | Yes |
| ENSMUSG00000026739 | Bmi1 | 0.3187 | 0.02495 | 0.3017 | 0.001079 | No | Yes |
| ENSMUSG00000026753 | Ppp6c | 0.3094 | 0.02094 | 0.303 | 0.000586 | No | Yes |
| ENSMUSG00000026796 | Niban2 | -0.5271 | 0.003564 | -0.7169 | 9.639e-14 | No | Yes |
| ENSMUSG00000026939 | Tmem141 | -0.6195 | 0.03244 | -0.5505 | 0.01794 | No | Yes |
| ENSMUSG00000026941 | Mamdc4 | 0.8029 | 2.247e-05 | 0.89 | 9.617e-09 | No | Yes |
| ENSMUSG00000026959 | Grin1 | 5.361 | 0.0004132 | 5.3342 | 0.0002964 | No | Yes |
| ENSMUSG00000027220 | Syt13 | -0.9994 | 0.01465 | -0.584 | 0.0004469 | No | Yes |
| ENSMUSG00000027248 | Pdia3 | -0.3123 | 0.0427 | -0.4675 | 3.002e-07 | No | Yes |
| ENSMUSG00000027249 | F2 | 1.7085 | 0.04252 | 2.538 | 0.006979 | No | Yes |
| ENSMUSG00000027254 | Map1a | -0.959 | 0.0003707 | -0.6063 | 4.889e-06 | No | Yes |
| ENSMUSG00000027272 | Ubr1 | 0.4983 | 8.251e-05 | 0.3493 | 9.638e-05 | No | Yes |
| ENSMUSG00000027309 | Dnaaf9 | -0.3564 | 0.0058 | -0.4122 | 2.123e-05 | No | Yes |
| ENSMUSG00000027339 | Rassf2 | -0.8242 | 0.03056 | -0.5679 | 0.001071 | No | Yes |
| ENSMUSG00000027346 | Gpcpd1 | -0.6083 | 0.0005264 | -0.7302 | 5.5e-05 | No | Yes |
| ENSMUSG00000027368 | Dusp2 | -0.9285 | 0.03466 | -0.8022 | 0.04447 | No | Yes |
| ENSMUSG00000027399 | Il1a | -0.9857 | 0.03395 | -1.297 | 0.001394 | No | Yes |
| ENSMUSG00000027460 | Angpt4 | -1.568 | 0.0009179 | -0.9688 | 0.002691 | No | Yes |
| ENSMUSG00000027488 | Snta1 | -1.186 | 0.01087 | -0.9355 | 0.0173 | No | Yes |
| ENSMUSG00000027489 | Necab3 | 0.5451 | 0.01792 | 0.9252 | 1.31e-05 | No | Yes |
| ENSMUSG00000027505 | Fam209 | 1.4231 | 0.0002757 | 1.4829 | 0.0001961 | No | Yes |
| ENSMUSG00000027533 | Fabp5 | -1.151 | 4.113e-05 | -1.211 | 6.267e-07 | No | Yes |
| ENSMUSG00000027605 | Acss2 | 0.3254 | 0.04273 | 0.2903 | 0.01969 | No | Yes |
| ENSMUSG00000027712 | Anxa5 | -0.2794 | 0.0115 | -0.5418 | 7.053e-09 | No | Yes |
| ENSMUSG00000027840 | Wnt2b | 0.8594 | 0.009914 | 0.8387 | 0.0008947 | No | Yes |
| ENSMUSG00000027843 | Ptpn22 | 0.6172 | 0.02837 | 0.8022 | 0.0004232 | No | Yes |
| ENSMUSG00000027848 | Olfml3 | -0.6241 | 0.004979 | -0.8718 | 7.45e-09 | No | Yes |
| ENSMUSG00000027859 | Ngf | -0.8172 | 0.03489 | -1.511 | 3.435e-08 | No | Yes |
| ENSMUSG00000027889 | Ampd2 | -0.3679 | 0.01073 | -0.3364 | 0.0001751 | No | Yes |
| ENSMUSG00000027896 | Slc16a4 | 0.5576 | 0.006085 | 0.6116 | 1.322e-06 | No | Yes |
| ENSMUSG00000027944 | Hax1 | -0.7476 | 0.00124 | -0.9324 | 5.014e-10 | No | Yes |
| ENSMUSG00000027967 | Neurog2 | 2.2362 | 0.00423 | 5.5602 | 2.974e-05 | No | Yes |
| ENSMUSG00000028034 | Fubp1 | 0.6922 | 8.448e-08 | 0.5791 | 2.841e-10 | No | Yes |
| ENSMUSG00000028048 | Gba | -0.3957 | 0.00206 | -0.2898 | 0.001509 | No | Yes |
| ENSMUSG00000028060 | Khdc4 | 0.47 | 0.0008189 | 0.3558 | 0.0003795 | No | Yes |
| ENSMUSG00000028088 | Fmo5 | -0.978 | 0.04573 | -0.9934 | 0.02151 | No | Yes |
| ENSMUSG00000028100 | Nudt17 | 0.8404 | 0.0154 | 1.0292 | 0.002303 | No | Yes |
| ENSMUSG00000028164 | Manba | -0.7153 | 4.25e-06 | -0.4266 | 0.0002089 | No | Yes |
| ENSMUSG00000028251 | Tstd3 | -0.723 | 0.002511 | -0.4998 | 0.007544 | No | Yes |
| ENSMUSG00000028270 | Gbp2 | -0.9511 | 0.006673 | -1.058 | 0.006243 | No | Yes |
| ENSMUSG00000028271 | Gtf2b | 0.3142 | 0.01331 | 0.2757 | 0.003386 | No | Yes |
| ENSMUSG00000028378 | Ptgr1 | -0.605 | 0.01219 | -0.2739 | 0.006763 | No | Yes |
| ENSMUSG00000028381 | Ugcg | -0.3491 | 0.02332 | -0.3191 | 0.0003006 | No | Yes |
| ENSMUSG00000028393 | Alad | -0.4719 | 0.001728 | -0.6114 | 9.833e-10 | No | Yes |
| ENSMUSG00000028410 | Dnaja1 | 0.3262 | 0.02386 | 0.5391 | 1.998e-08 | No | Yes |
| ENSMUSG00000028413 | B4galt1 | -0.5481 | 5.106e-05 | -0.3674 | 1.097e-05 | No | Yes |
| ENSMUSG00000028414 | Fktn | 0.5681 | 0.0004613 | 0.5184 | 3.43e-08 | No | Yes |
| ENSMUSG00000028441 | 1110017D15Rik | 1.7662 | 0.0139 | 1.5146 | 0.02216 | No | Yes |
| ENSMUSG00000028494 | Plin2 | -0.881 | 0.00465 | -0.3834 | 0.001129 | No | Yes |
| ENSMUSG00000028525 | Pde4b | -3.516 | 0.006983 | -3.197 | 0.002971 | No | Yes |
| ENSMUSG00000028583 | Pdpn | -0.9503 | 0.007498 | -0.5477 | 1.76e-05 | No | Yes |
| ENSMUSG00000028602 | Tnfrsf8 | -1.519 | 7.867e-07 | -1.078 | 0.0001484 | No | Yes |
| ENSMUSG00000028621 | Cyb5rl | -0.4772 | 0.03437 | -0.4838 | 0.002484 | No | Yes |
| ENSMUSG00000028634 | Hivep3 | -0.9871 | 1.21e-06 | -0.6 | 0.001099 | No | Yes |
| ENSMUSG00000028743 | Akr7a5 | -0.499 | 0.01074 | -0.4272 | 0.005373 | No | Yes |
| ENSMUSG00000028755 | Cda | -0.9481 | 0.01097 | -0.9078 | 8.316e-05 | No | Yes |
| ENSMUSG00000028763 | Hspg2 | -0.6203 | 2.568e-07 | -0.7472 | 1.084e-12 | No | Yes |
| ENSMUSG00000028776 | Tinagl1 | -1.459 | 6.691e-07 | -1.222 | 7.563e-08 | No | Yes |
| ENSMUSG00000028842 | Ago3 | 0.4028 | 0.001914 | 0.4432 | 7.919e-06 | No | Yes |
| ENSMUSG00000028885 | Smpdl3b | -0.9222 | 0.0001271 | -0.8146 | 6.63e-07 | No | Yes |
| ENSMUSG00000028980 | H6pd | -0.7269 | 0.03857 | -0.3858 | 0.006982 | No | Yes |
| ENSMUSG00000029003 | Mad2l2 | -0.3302 | 0.02417 | -0.4196 | 0.0002143 | No | Yes |
| ENSMUSG00000029027 | Dffb | 0.4368 | 0.04146 | 0.4719 | 0.004653 | No | Yes |
| ENSMUSG00000029068 | Ccnl2 | 0.3826 | 0.04854 | 0.2954 | 0.0001577 | No | Yes |
| ENSMUSG00000029072 | Tas1r3 | 0.8466 | 0.02545 | 1.097 | 8.164e-05 | No | Yes |
| ENSMUSG00000029086 | Prom1 | 5.9993 | 0.0003308 | 5.9743 | 0.0001498 | No | Yes |
| ENSMUSG00000029123 | Stk32b | -3.475 | 0.0001358 | -2.791 | 0.002788 | No | Yes |
| ENSMUSG00000029134 | Plb1 | -1.842 | 0.01162 | -1.496 | 0.003512 | No | Yes |
| ENSMUSG00000029136 | Rbks | -0.7809 | 0.03835 | -0.7167 | 0.04856 | No | Yes |
| ENSMUSG00000029174 | Tbc1d1 | -0.5818 | 0.004974 | -0.279 | 0.03933 | No | Yes |
| ENSMUSG00000029361 | Nos1 | -1.439 | 0.0399 | -1.361 | 0.03756 | No | Yes |
| ENSMUSG00000029375 | Cxcl15 | -2.074 | 6.184e-05 | -1.455 | 0.03787 | No | Yes |
| ENSMUSG00000029401 | Rilpl2 | -0.4556 | 0.02809 | -0.5893 | 0.0001198 | No | Yes |
| ENSMUSG00000029470 | P2rx4 | -0.6693 | 1.227e-06 | -0.6562 | 6.119e-08 | No | Yes |
| ENSMUSG00000029478 | Ncor2 | -0.5446 | 1.07e-05 | -0.3723 | 0.0002374 | No | Yes |
| ENSMUSG00000029490 | Mfsd7a | -0.6903 | 0.04169 | -0.6033 | 0.009536 | No | Yes |
| ENSMUSG00000029517 | Ankrd7 | 6.3223 | 6.589e-07 | 4.2452 | 2.37e-05 | No | Yes |
| ENSMUSG00000029521 | Chek2 | 0.3024 | 0.01164 | 0.2952 | 0.005689 | No | Yes |
| ENSMUSG00000029570 | Lfng | -1.116 | 0.006465 | -1.297 | 1.833e-06 | No | Yes |
| ENSMUSG00000029591 | Ung | -0.4947 | 0.003898 | -0.5777 | 3.141e-05 | No | Yes |
| ENSMUSG00000029658 | Wdr95 | -1.396 | 0.005166 | -1.614 | 0.0003141 | No | Yes |
| ENSMUSG00000029810 | Tmem176b | -0.8002 | 0.004056 | -0.7312 | 0.00535 | No | Yes |
| ENSMUSG00000029832 | Nfe2l3 | 0.8046 | 0.01964 | 1.2194 | 2.296e-07 | No | Yes |
| ENSMUSG00000029998 | Pcyox1 | -0.6099 | 0.002099 | -0.278 | 0.002587 | No | Yes |
| ENSMUSG00000030029 | Lrig1 | -0.4928 | 0.03408 | -0.7227 | 2.495e-05 | No | Yes |
| ENSMUSG00000030084 | Plxna1 | -0.3695 | 0.006861 | -0.2753 | 0.003336 | No | Yes |
| ENSMUSG00000030096 | Slc6a6 | -0.7831 | 2.303e-07 | -0.4838 | 0.0002888 | No | Yes |
| ENSMUSG00000030116 | Mfap5 | -0.4922 | 0.04521 | -0.7642 | 6.026e-05 | No | Yes |
| ENSMUSG00000030123 | Plxnd1 | -0.7703 | 0.001113 | -0.3378 | 0.01164 | No | Yes |
| ENSMUSG00000030131 | Mug2 | 1.3675 | 0.01828 | 2.7761 | 0.0008441 | No | Yes |
| ENSMUSG00000030214 | Plbd1 | 1.9646 | 0.001574 | 2.4595 | 0.0003063 | No | Yes |
| ENSMUSG00000030255 | Sspn | -0.4034 | 0.02071 | -0.518 | 0.001031 | No | Yes |
| ENSMUSG00000030257 | Srgap3 | -0.4653 | 0.02977 | -0.6742 | 2.401e-06 | No | Yes |
| ENSMUSG00000030263 | Irag2 | -1.291 | 0.006929 | -1.399 | 0.001242 | No | Yes |
| ENSMUSG00000030386 | Zfp606 | 0.4345 | 0.00316 | 0.4319 | 2.163e-05 | No | Yes |
| ENSMUSG00000030393 | Zik1 | 0.4102 | 0.01025 | 0.3118 | 0.008378 | No | Yes |
| ENSMUSG00000030543 | Mesp2 | 4.1239 | 0.03825 | 4.0988 | 0.03717 | No | Yes |
| ENSMUSG00000030583 | Sipa1l3 | -0.2648 | 0.04415 | -0.3422 | 0.002594 | No | Yes |
| ENSMUSG00000030695 | Aldoa | -0.6704 | 0.009299 | -0.286 | 0.04178 | No | Yes |
| ENSMUSG00000030744 | Rps3 | -0.2671 | 0.04176 | -0.3631 | 9.758e-06 | No | Yes |
| ENSMUSG00000030748 | Il4ra | -0.7556 | 0.003795 | -0.861 | 2.168e-09 | No | Yes |
| ENSMUSG00000030760 | Acer3 | 0.2909 | 0.02699 | 0.3229 | 0.0005345 | No | Yes |
| ENSMUSG00000030781 | Slc5a2 | 1.1013 | 0.03907 | 2.5638 | 9.657e-05 | No | Yes |
| ENSMUSG00000030793 | Pycard | 2.207 | 0.00956 | 1.4055 | 0.04829 | No | Yes |
| ENSMUSG00000030868 | Dctn5 | 0.3619 | 0.002195 | 0.4577 | 2.779e-07 | No | Yes |
| ENSMUSG00000031098 | Syt8 | -0.901 | 0.004096 | -0.9374 | 0.0001888 | No | Yes |
| ENSMUSG00000031142 | Cacna1f | -1.662 | 0.0134 | -1.415 | 0.03535 | No | Yes |
| ENSMUSG00000031146 | Plp2 | -0.3136 | 0.01237 | -0.5459 | 5.936e-08 | No | Yes |
| ENSMUSG00000031200 | Mtcp1 | 0.5118 | 0.01351 | 0.4271 | 0.0002094 | No | Yes |
| ENSMUSG00000031201 | Brcc3 | 0.4927 | 0.004103 | 0.4051 | 1.199e-05 | No | Yes |
| ENSMUSG00000031302 | Nlgn3 | 1.6011 | 2.72e-05 | 2.1413 | 1.653e-08 | No | Yes |
| ENSMUSG00000031303 | Map3k15 | 1.2801 | 0.01619 | 1.4858 | 0.003105 | No | Yes |
| ENSMUSG00000031389 | Arhgap4 | 0.8975 | 0.009374 | 0.6861 | 0.01217 | No | Yes |
| ENSMUSG00000031411 | Pramex1 | 4.9842 | 0.001702 | 4.9576 | 0.002293 | No | Yes |
| ENSMUSG00000031433 | Rbm41 | 0.3572 | 0.02768 | 0.4376 | 0.0001297 | No | Yes |
| ENSMUSG00000031442 | Mcf2l | -0.802 | 3.095e-06 | -0.6718 | 1.749e-06 | No | Yes |
| ENSMUSG00000031443 | F7 | -1.869 | 0.001315 | -1.234 | 0.02987 | No | Yes |
| ENSMUSG00000031465 | Angpt2 | 0.7198 | 0.04495 | 0.961 | 0.0003748 | No | Yes |
| ENSMUSG00000031502 | Col4a1 | -0.5833 | 2.179e-07 | -0.8136 | 8.891e-18 | No | Yes |
| ENSMUSG00000031506 | Ptpn7 | -0.836 | 0.003014 | -0.6984 | 0.002406 | No | Yes |
| ENSMUSG00000031516 | Dctn6 | 0.2657 | 0.04703 | 0.3194 | 0.0006533 | No | Yes |
| ENSMUSG00000031530 | Dusp4 | -0.8412 | 0.00124 | -1.203 | 2.43e-20 | No | Yes |
| ENSMUSG00000031565 | Fgfr1 | -0.5724 | 0.0007595 | -0.6771 | 1.144e-11 | No | Yes |
| ENSMUSG00000031586 | Rbpms | -0.4259 | 0.01541 | -0.4413 | 3.744e-06 | No | Yes |
| ENSMUSG00000031600 | Vps37a | 0.4885 | 0.003774 | 0.4177 | 3.875e-06 | No | Yes |
| ENSMUSG00000031662 | Snx20 | -0.5851 | 0.003975 | -0.395 | 0.01695 | No | Yes |
| ENSMUSG00000031732 | Phlpp2 | 0.37 | 0.002307 | 0.3135 | 0.0008656 | No | Yes |
| ENSMUSG00000031750 | Il34 | -0.7532 | 0.04799 | -1.294 | 1.777e-05 | No | Yes |
| ENSMUSG00000031765 | Mt1 | -0.6883 | 1.658e-06 | -0.5626 | 0.0001986 | No | Yes |
| ENSMUSG00000031778 | Cx3cl1 | -1.141 | 0.002062 | -0.9041 | 1.062e-07 | No | Yes |
| ENSMUSG00000031808 | Slc27a1 | -0.8271 | 1.123e-10 | -0.7856 | 2.554e-14 | No | Yes |
| ENSMUSG00000031825 | Crispld2 | -2.283 | 0.00275 | -2.062 | 1.804e-07 | No | Yes |
| ENSMUSG00000031875 | Cmtm3 | -0.4026 | 0.0008428 | -0.3345 | 0.0004836 | No | Yes |
| ENSMUSG00000031906 | Smpd3 | -0.8869 | 0.03045 | -1.035 | 0.007237 | No | Yes |
| ENSMUSG00000031930 | Wwp2 | -0.2739 | 0.01632 | -0.4146 | 3.087e-07 | No | Yes |
| ENSMUSG00000031932 | Gpr83 | 4.4838 | 0.01526 | 4.455 | 0.01288 | No | Yes |
| ENSMUSG00000032038 | St3gal4 | -0.4656 | 0.006111 | -0.4998 | 3.76e-05 | No | Yes |
| ENSMUSG00000032123 | Dpagt1 | -0.3789 | 0.008578 | -0.5913 | 3.28e-10 | No | Yes |
| ENSMUSG00000032131 | Abcg4 | 0.6906 | 0.02516 | 1.3956 | 8.008e-11 | No | Yes |
| ENSMUSG00000032207 | Lipc | -1.931 | 0.002854 | -1.629 | 0.01064 | No | Yes |
| ENSMUSG00000032245 | Cln6 | -0.5433 | 0.00813 | -0.5727 | 0.0005895 | No | Yes |
| ENSMUSG00000032278 | Paqr5 | -5.093 | 0.002816 | -4.292 | 0.04357 | No | Yes |
| ENSMUSG00000032281 | Acsbg1 | -1.902 | 1.325e-11 | -1.594 | 2.162e-08 | No | Yes |
| ENSMUSG00000032294 | Pkm | -0.383 | 0.002455 | -0.3897 | 4.151e-05 | No | Yes |
| ENSMUSG00000032338 | Hcn4 | 2.598 | 0.04429 | 2.5422 | 0.03749 | No | Yes |
| ENSMUSG00000032456 | Nmnat3 | -0.9949 | 0.0008688 | -0.8857 | 0.0002499 | No | Yes |
| ENSMUSG00000032501 | Trib1 | -0.5583 | 0.01695 | -0.3859 | 0.003798 | No | Yes |
| ENSMUSG00000032527 | Pccb | 0.2957 | 0.02736 | 0.301 | 0.00324 | No | Yes |
| ENSMUSG00000032548 | Slco2a1 | -0.511 | 0.0008321 | -0.5277 | 9.482e-07 | No | Yes |
| ENSMUSG00000032611 | 1700102P08Rik | 1.4937 | 0.0002918 | 1.7356 | 4.717e-07 | No | Yes |
| ENSMUSG00000032641 | Gpr19 | -0.6679 | 0.02284 | -0.6191 | 0.004898 | No | Yes |
| ENSMUSG00000032657 | Fam189b | 1.2293 | 0.000482 | 0.7045 | 0.01238 | No | Yes |
| ENSMUSG00000032766 | Gng11 | 0.3552 | 0.02243 | 0.5505 | 6.335e-05 | No | Yes |
| ENSMUSG00000032812 | Arap1 | -0.5423 | 0.002274 | -0.4902 | 3.318e-05 | No | Yes |
| ENSMUSG00000032842 | Abcc10 | -0.4526 | 0.01002 | -0.7167 | 9.089e-08 | No | Yes |
| ENSMUSG00000032860 | P2ry2 | -0.655 | 0.001355 | -0.4126 | 0.013 | No | Yes |
| ENSMUSG00000032902 | Slc16a1 | -0.4538 | 8.582e-05 | -0.5622 | 2.741e-10 | No | Yes |
| ENSMUSG00000032908 | Sgpp2 | -1.715 | 0.01448 | -1.798 | 0.002913 | No | Yes |
| ENSMUSG00000032942 | Ucp3 | -1.512 | 0.01234 | -1.166 | 0.04429 | No | Yes |
| ENSMUSG00000032946 | Rasgrp2 | 1.6731 | 0.008613 | 1.8799 | 0.01824 | No | Yes |
| ENSMUSG00000033004 | Mycbp2 | 0.3016 | 0.02035 | 0.3281 | 0.0003627 | No | Yes |
| ENSMUSG00000033177 | Tmprss7 | 2.3367 | 0.001334 | 3.7108 | 5.16e-06 | No | Yes |
| ENSMUSG00000033187 | BC016579 | 2.5863 | 0.005412 | 2.9888 | 0.004955 | No | Yes |
| ENSMUSG00000033287 | Kctd17 | -0.5384 | 0.0003857 | -0.577 | 1.52e-07 | No | Yes |
| ENSMUSG00000033318 | Gstt2 | -0.7856 | 0.007418 | -0.6997 | 0.01675 | No | Yes |
| ENSMUSG00000033419 | Snap91 | -2.011 | 0.0001075 | -1.608 | 0.01355 | No | Yes |
| ENSMUSG00000033545 | Znrf1 | -0.375 | 0.01649 | -0.4944 | 4.67e-05 | No | Yes |
| ENSMUSG00000033615 | Cplx1 | 4.604 | 0.008479 | 4.5806 | 0.007085 | No | Yes |
| ENSMUSG00000033684 | Qsox1 | -0.402 | 0.001143 | -0.4324 | 3.383e-07 | No | Yes |
| ENSMUSG00000033715 | Akr1c14 | -1.688 | 0.007831 | -2.145 | 2.379e-06 | No | Yes |
| ENSMUSG00000033760 | Rbm4b | 0.4698 | 0.0103 | 0.3361 | 0.000222 | No | Yes |
| ENSMUSG00000033847 | Pla2g4c | 1.5411 | 3.101e-07 | 1.0537 | 8.651e-08 | No | Yes |
| ENSMUSG00000033863 | Klf9 | -0.4359 | 0.0005262 | -0.4603 | 3.163e-06 | No | Yes |
| ENSMUSG00000033964 | Zbtb41 | 0.4377 | 0.02051 | 0.286 | 0.03032 | No | Yes |
| ENSMUSG00000033972 | Zfp944 | 0.2869 | 0.02594 | 0.3882 | 0.001855 | No | Yes |
| ENSMUSG00000034040 | Galnt17 | -2.216 | 0.001875 | -2.806 | 0.001434 | No | Yes |
| ENSMUSG00000034071 | Zfp551 | 0.5772 | 0.009936 | 0.7208 | 2.392e-05 | No | Yes |
| ENSMUSG00000034160 | Ogt | 0.5781 | 0.001834 | 0.2941 | 0.0004296 | No | Yes |
| ENSMUSG00000034163 | Zfc3h1 | 0.7283 | 0.001593 | 0.4803 | 7.585e-07 | No | Yes |
| ENSMUSG00000034206 | Polq | 0.2832 | 0.02633 | 0.4669 | 4.498e-06 | No | Yes |
| ENSMUSG00000034394 | Lif | -0.8169 | 0.04364 | -1.215 | 2.204e-05 | No | Yes |
| ENSMUSG00000034601 | 2700049A03Rik | 0.2972 | 0.01912 | 0.45 | 2.603e-05 | No | Yes |
| ENSMUSG00000034614 | Pik3ip1 | -0.7095 | 0.03057 | -0.835 | 0.0003468 | No | Yes |
| ENSMUSG00000034647 | Ankrd12 | 0.2677 | 0.01474 | 0.3644 | 2.458e-05 | No | Yes |
| ENSMUSG00000034652 | Cd300a | -4.541 | 0.03026 | -4.288 | 0.04674 | No | Yes |
| ENSMUSG00000034744 | Nagk | -0.4224 | 0.0009888 | -0.2751 | 0.002814 | No | Yes |
| ENSMUSG00000034818 | Celf5 | 1.0019 | 3.733e-05 | 0.8948 | 5.528e-05 | No | Yes |
| ENSMUSG00000034832 | Tet3 | 0.3564 | 0.01607 | 0.4422 | 5.685e-06 | No | Yes |
| ENSMUSG00000034872 | Gipc3 | -0.7288 | 0.01334 | -0.7687 | 0.002807 | No | Yes |
| ENSMUSG00000034917 | Tjp3 | 2.089 | 3.203e-05 | 3.9095 | 1.144e-09 | No | Yes |
| ENSMUSG00000034994 | Eef2 | -0.2888 | 0.01074 | -0.3531 | 2.464e-05 | No | Yes |
| ENSMUSG00000035062 | Zc4h2 | 0.3758 | 0.03683 | 0.6644 | 6.193e-05 | No | Yes |
| ENSMUSG00000035164 | Zc3h12c | 0.3701 | 0.03517 | 0.3723 | 0.008068 | No | Yes |
| ENSMUSG00000035206 | Sppl2b | -0.3526 | 0.03855 | -0.5069 | 0.0001572 | No | Yes |
| ENSMUSG00000035273 | Hpse | -0.681 | 0.006137 | -0.3084 | 0.006151 | No | Yes |
| ENSMUSG00000035314 | Gdpd5 | -0.56 | 0.01775 | -0.6119 | 0.0001063 | No | Yes |
| ENSMUSG00000035342 | Lzts2 | -0.4123 | 0.03043 | -0.4546 | 4.944e-05 | No | Yes |
| ENSMUSG00000035506 | Slc12a8 | -1.793 | 1.998e-05 | -1.213 | 7.533e-12 | No | Yes |
| ENSMUSG00000035576 | L3mbtl1 | 1.397 | 0.001486 | 2.0355 | 8.231e-07 | No | Yes |
| ENSMUSG00000035597 | Prpf39 | 0.3037 | 0.009119 | 0.3225 | 0.0003263 | No | Yes |
| ENSMUSG00000035778 | Ggta1 | -0.4525 | 0.01038 | -0.6744 | 1.41e-12 | No | Yes |
| ENSMUSG00000035790 | Cep19 | 0.4685 | 0.0004652 | 0.3479 | 0.001656 | No | Yes |
| ENSMUSG00000035828 | Pim3 | -0.5853 | 0.00387 | -0.777 | 1.875e-08 | No | Yes |
| ENSMUSG00000035847 | Ids | 0.4418 | 0.001895 | 0.3588 | 6.197e-05 | No | Yes |
| ENSMUSG00000035896 | Rnase1 | -4.646 | 0.0001908 | -3.101 | 5.248e-05 | No | Yes |
| ENSMUSG00000035944 | Ttc38 | -1.423 | 0.01863 | -1.484 | 0.001498 | No | Yes |
| ENSMUSG00000036155 | Mgat5 | -0.5254 | 0.005278 | -0.2894 | 0.003156 | No | Yes |
| ENSMUSG00000036197 | Gxylt1 | 0.445 | 0.003981 | 0.3581 | 0.0006819 | No | Yes |
| ENSMUSG00000036291 | Ap5m1 | 0.4921 | 0.0001515 | 0.6296 | 1.822e-09 | No | Yes |
| ENSMUSG00000036292 | Gramd1c | 0.9432 | 0.001499 | 0.5182 | 0.01353 | No | Yes |
| ENSMUSG00000036430 | Tbcc | -0.8933 | 0.02645 | -0.7256 | 0.001452 | No | Yes |
| ENSMUSG00000036469 | Marchf1 | 2.6993 | 0.02201 | 4.7132 | 0.003632 | No | Yes |
| ENSMUSG00000036480 | Prss56 | -2.566 | 0.00278 | -2.217 | 0.02106 | No | Yes |
| ENSMUSG00000036557 | Stpg4 | 2.9623 | 0.007457 | 2.0977 | 0.03698 | No | Yes |
| ENSMUSG00000036632 | Alg5 | -0.3183 | 0.03312 | -0.4393 | 0.0004338 | No | Yes |
| ENSMUSG00000036718 | Micall2 | -1.265 | 0.0006929 | -1.493 | 2.731e-17 | No | Yes |
| ENSMUSG00000036721 | Zscan12 | 0.4365 | 0.009449 | 0.4436 | 0.0007269 | No | Yes |
| ENSMUSG00000036854 | Hspb6 | -0.4923 | 0.01102 | -0.7576 | 4.892e-06 | No | Yes |
| ENSMUSG00000036858 | Ptcra | 1.4562 | 0.000534 | 1.4539 | 0.002051 | No | Yes |
| ENSMUSG00000037017 | Zscan21 | 0.2927 | 0.04481 | 0.4062 | 0.0001921 | No | Yes |
| ENSMUSG00000037126 | Psd | 0.5222 | 0.03132 | 0.4808 | 0.01046 | No | Yes |
| ENSMUSG00000037217 | Syn1 | 0.9619 | 0.003056 | 1.4601 | 8.069e-06 | No | Yes |
| ENSMUSG00000037259 | Dzank1 | 0.4522 | 0.01972 | 0.5415 | 0.0007798 | No | Yes |
| ENSMUSG00000037262 | Kin | 0.4148 | 0.008544 | 0.4386 | 7.911e-05 | No | Yes |
| ENSMUSG00000037266 | Rsrp1 | 0.5112 | 0.0005392 | 0.5154 | 2.428e-06 | No | Yes |
| ENSMUSG00000037270 | 4932438A13Rik | 0.2853 | 0.01132 | 0.3076 | 0.0003575 | No | Yes |
| ENSMUSG00000037344 | Slc12a9 | -0.4909 | 0.0004275 | -0.2839 | 0.01534 | No | Yes |
| ENSMUSG00000037364 | Srrt | 0.5598 | 0.009376 | 0.5343 | 0.0003542 | No | Yes |
| ENSMUSG00000037411 | Serpine1 | -2.341 | 0.002867 | -1.997 | 4.056e-05 | No | Yes |
| ENSMUSG00000037440 | Vnn1 | -0.946 | 0.005464 | -1.065 | 0.004624 | No | Yes |
| ENSMUSG00000037568 | Vash2 | -0.5792 | 0.02522 | -0.8349 | 6.019e-07 | No | Yes |
| ENSMUSG00000037617 | Spag1 | 0.6535 | 0.0005771 | 0.357 | 0.004127 | No | Yes |
| ENSMUSG00000037679 | Inf2 | -0.7176 | 0.0001716 | -0.8194 | 5.738e-13 | No | Yes |
| ENSMUSG00000037857 | Nufip2 | 0.3962 | 0.01867 | 0.5678 | 2.207e-07 | No | Yes |
| ENSMUSG00000037936 | Scarb1 | -0.3103 | 0.008492 | -0.3761 | 0.0003747 | No | Yes |
| ENSMUSG00000037946 | Fgd3 | -0.5853 | 0.01314 | -0.8348 | 2.176e-05 | No | Yes |
| ENSMUSG00000037974 | Muc5ac | 5.1787 | 0.0004576 | 3.0918 | 0.01126 | No | Yes |
| ENSMUSG00000037992 | Rara | -0.5483 | 0.03629 | -0.3339 | 0.03191 | No | Yes |
| ENSMUSG00000038034 | Igsf8 | -0.3761 | 0.03145 | -0.3016 | 0.01567 | No | Yes |
| ENSMUSG00000038037 | Socs1 | -1.154 | 0.005044 | -1.318 | 0.0006061 | No | Yes |
| ENSMUSG00000038217 | Tlcd2 | -0.4427 | 0.04435 | -0.5252 | 0.0002504 | No | Yes |
| ENSMUSG00000038224 | Serpinf2 | 1.0862 | 0.0042 | 1.5003 | 5.132e-05 | No | Yes |
| ENSMUSG00000038250 | Usp38 | 0.3345 | 0.004678 | 0.5681 | 9.697e-11 | No | Yes |
| ENSMUSG00000038260 | Trpm4 | -0.6118 | 0.01472 | -0.408 | 0.01499 | No | Yes |
| ENSMUSG00000038527 | C1rl | -0.7617 | 0.02153 | -0.7463 | 2.955e-05 | No | Yes |
| ENSMUSG00000038578 | Susd1 | -0.7234 | 0.002941 | -0.5474 | 0.0001825 | No | Yes |
| ENSMUSG00000038838 | Vars2 | -0.3528 | 0.0274 | -0.4725 | 0.0001907 | No | Yes |
| ENSMUSG00000038949 | Cnst | 0.292 | 0.02093 | 0.3699 | 0.0001208 | No | Yes |
| ENSMUSG00000039055 | Eme1 | -0.5337 | 0.03076 | -0.5976 | 0.00099 | No | Yes |
| ENSMUSG00000039099 | Wdr93 | -1.419 | 4.3e-08 | -1.374 | 3.098e-17 | No | Yes |
| ENSMUSG00000039154 | Shd | 0.971 | 0.025 | 1.3153 | 1.329e-05 | No | Yes |
| ENSMUSG00000039195 | Bbln | -0.6763 | 0.001798 | -0.7373 | 6.157e-05 | No | Yes |
| ENSMUSG00000039239 | Tgfb2 | 1.6539 | 0.002255 | 2.0348 | 0.0001991 | No | Yes |
| ENSMUSG00000039347 | Atp6v0e2 | -0.5869 | 0.01079 | -0.4368 | 0.001863 | No | Yes |
| ENSMUSG00000039357 | Fut11 | 0.4424 | 0.003704 | 0.315 | 0.007419 | No | Yes |
| ENSMUSG00000039431 | Mtmr7 | 0.6311 | 0.04274 | 0.5059 | 0.03219 | No | Yes |
| ENSMUSG00000039531 | Zup1 | 0.3337 | 0.03387 | 0.5759 | 2.529e-06 | No | Yes |
| ENSMUSG00000039577 | Nphp4 | -0.3963 | 0.03 | -0.684 | 7.554e-07 | No | Yes |
| ENSMUSG00000039789 | Zfp597 | 0.4064 | 0.008473 | 0.5421 | 1.117e-05 | No | Yes |
| ENSMUSG00000039831 | Arhgap29 | -0.5008 | 0.008897 | -0.4171 | 0.002979 | No | Yes |
| ENSMUSG00000039841 | Zfp800 | 0.3894 | 0.006123 | 0.3018 | 0.004276 | No | Yes |
| ENSMUSG00000039981 | Zc3h12d | -1.298 | 0.02172 | -1.154 | 0.0001386 | No | Yes |
| ENSMUSG00000039997 | Ifi203 | -1.005 | 0.009704 | -0.54 | 5.825e-07 | No | Yes |
| ENSMUSG00000040026 | Saa3 | -0.8735 | 0.04094 | -1.603 | 3.059e-06 | No | Yes |
| ENSMUSG00000040123 | Zmym5 | 0.4894 | 5.532e-05 | 0.3758 | 3.15e-05 | No | Yes |
| ENSMUSG00000040205 | Cuzd1 | 2.9883 | 0.0002283 | 3.8169 | 7.247e-09 | No | Yes |
| ENSMUSG00000040219 | Ttc12 | -0.3555 | 0.02372 | -0.4393 | 0.0002449 | No | Yes |
| ENSMUSG00000040249 | Lrp1 | -0.5902 | 0.007965 | -0.5122 | 3.237e-06 | No | Yes |
| ENSMUSG00000040250 | Ints13 | 0.3753 | 0.002839 | 0.2772 | 0.00293 | No | Yes |
| ENSMUSG00000040554 | Aipl1 | -0.9803 | 0.02438 | -1.451 | 0.001165 | No | Yes |
| ENSMUSG00000040714 | Klc3 | -1.205 | 0.02529 | -1.107 | 0.02884 | No | Yes |
| ENSMUSG00000040738 | Ints8 | 0.2907 | 0.01334 | 0.2763 | 0.00316 | No | Yes |
| ENSMUSG00000040943 | Tet2 | 0.3622 | 0.009485 | 0.28 | 0.00185 | No | Yes |
| ENSMUSG00000041073 | Nacad | 0.9713 | 0.003847 | 1.7012 | 3.656e-07 | No | Yes |
| ENSMUSG00000041096 | Tspyl2 | 0.3126 | 0.006961 | 0.5418 | 9.126e-10 | No | Yes |
| ENSMUSG00000041117 | Ccdc8 | 2.9211 | 0.03298 | 4.9492 | 0.004653 | No | Yes |
| ENSMUSG00000041323 | Ak7 | 1.7801 | 0.0004357 | 2.8371 | 3.915e-07 | No | Yes |
| ENSMUSG00000041328 | Pcf11 | 0.5103 | 0.01956 | 0.3987 | 0.001846 | No | Yes |
| ENSMUSG00000041566 | Tssk1 | 1.4782 | 1.15e-05 | 1.7427 | 9.814e-05 | No | Yes |
| ENSMUSG00000041577 | Prelp | -1.855 | 0.02971 | -0.7913 | 0.008159 | No | Yes |
| ENSMUSG00000041608 | Entpd3 | -2.596 | 0.02037 | -2.427 | 0.001797 | No | Yes |
| ENSMUSG00000041736 | Tspo | -0.5969 | 0.002753 | -0.6089 | 1.131e-06 | No | Yes |
| ENSMUSG00000041831 | Sytl3 | -1.273 | 0.01066 | -1.023 | 0.04524 | No | Yes |
| ENSMUSG00000041846 | Ppp4r3a | 0.3611 | 0.005108 | 0.2728 | 0.001795 | No | Yes |
| ENSMUSG00000041872 | Il17f | 1.3405 | 0.0002694 | 1.6035 | 1.61e-07 | No | Yes |
| ENSMUSG00000041895 | Wipi1 | -0.5265 | 0.01478 | -0.5698 | 2.832e-07 | No | Yes |
| ENSMUSG00000041911 | Dlx1 | 0.5107 | 0.03782 | 0.4593 | 0.02672 | No | Yes |
| ENSMUSG00000041975 | Mettl8 | -0.472 | 0.02333 | -0.734 | 1.356e-07 | No | Yes |
| ENSMUSG00000042082 | Arsb | -0.2729 | 0.02298 | -0.3694 | 5.455e-05 | No | Yes |
| ENSMUSG00000042138 | Msantd2 | 0.3549 | 0.02001 | 0.3297 | 0.0006588 | No | Yes |
| ENSMUSG00000042197 | Zfp451 | 0.2793 | 0.04046 | 0.2656 | 0.01151 | No | Yes |
| ENSMUSG00000042272 | Sestd1 | -0.358 | 0.02255 | -0.2901 | 0.0008771 | No | Yes |
| ENSMUSG00000042404 | Dennd4b | -0.5226 | 0.002861 | -0.6379 | 9.535e-06 | No | Yes |
| ENSMUSG00000042508 | Dmtf1 | 0.4071 | 0.01622 | 0.3967 | 0.0001485 | No | Yes |
| ENSMUSG00000042529 | Kcnj12 | -4.686 | 0.005952 | -5.758 | 0.002056 | No | Yes |
| ENSMUSG00000042595 | Fam199x | 0.5212 | 0.004513 | 0.4221 | 0.001025 | No | Yes |
| ENSMUSG00000042751 | Nmnat2 | -1.118 | 0.02893 | -0.8998 | 0.02814 | No | Yes |
| ENSMUSG00000042793 | Lgr6 | -1.695 | 0.0003936 | -1.168 | 1.019e-06 | No | Yes |
| ENSMUSG00000043017 | Ptgir | -0.4587 | 0.04336 | -0.3573 | 0.01706 | No | Yes |
| ENSMUSG00000043219 | Hoxa6 | 0.5566 | 0.004703 | 0.7293 | 6.957e-05 | No | Yes |
| ENSMUSG00000043313 | Pcdhb19 | 0.55 | 0.02175 | 0.916 | 1.832e-05 | No | Yes |
| ENSMUSG00000043467 | Zbtb37 | 0.4062 | 0.0004726 | 0.506 | 9.941e-08 | No | Yes |
| ENSMUSG00000043468 | Adam30 | 2.5903 | 0.03361 | 4.5895 | 0.006241 | No | Yes |
| ENSMUSG00000043631 | Ecm2 | 1.397 | 0.02174 | 2.5954 | 0.0003141 | No | Yes |
| ENSMUSG00000043633 | Fam221b | 1.439 | 0.0005594 | 0.8938 | 0.01454 | No | Yes |
| ENSMUSG00000043671 | Dpy19l3 | -0.4641 | 0.03216 | -0.3553 | 0.01778 | No | Yes |
| ENSMUSG00000043822 | Adamtsl5 | -0.5678 | 0.00336 | -0.3702 | 0.01587 | No | Yes |
| ENSMUSG00000043923 | Ccdc84 | 0.4706 | 0.002036 | 0.3844 | 0.0009206 | No | Yes |
| ENSMUSG00000043943 | Naalad2 | 0.3846 | 0.002705 | 0.4932 | 1.666e-07 | No | Yes |
| ENSMUSG00000043953 | Ccrl2 | -1.055 | 0.04096 | -0.8589 | 0.04645 | No | Yes |
| ENSMUSG00000044165 | Bcl2l15 | 0.9286 | 0.002128 | 0.6232 | 0.02809 | No | Yes |
| ENSMUSG00000044367 | Slc16a13 | -0.8967 | 0.01235 | -0.5074 | 0.01697 | No | Yes |
| ENSMUSG00000044390 | Tigd3 | 1.2059 | 1.191e-05 | 1.3598 | 7.115e-08 | No | Yes |
| ENSMUSG00000044405 | Adig | 2.2217 | 0.000246 | 3.0854 | 6.281e-06 | No | Yes |
| ENSMUSG00000044595 | Dnd1 | 1.1339 | 0.004219 | 0.8739 | 0.01933 | No | Yes |
| ENSMUSG00000044881 | Coa4 | -0.4054 | 0.0314 | -0.3336 | 0.0272 | No | Yes |
| ENSMUSG00000045094 | Arhgef37 | -1.347 | 0.001443 | -1.876 | 2.672e-14 | No | Yes |
| ENSMUSG00000045362 | Tnfrsf26 | -0.4895 | 0.006767 | -0.5257 | 5.593e-05 | No | Yes |
| ENSMUSG00000045672 | Col27a1 | 1.5518 | 0.0415 | 1.7954 | 0.02897 | No | Yes |
| ENSMUSG00000046020 | Pofut1 | -0.4583 | 4.272e-05 | -0.7112 | 2.563e-17 | No | Yes |
| ENSMUSG00000046138 | 9930021J03Rik | 0.3223 | 0.02076 | 0.3564 | 0.001355 | No | Yes |
| ENSMUSG00000046191 | Pcdhb20 | 0.6892 | 0.02053 | 0.91 | 6.607e-06 | No | Yes |
| ENSMUSG00000046223 | Plaur | -0.7642 | 0.008866 | -1.084 | 0.0001498 | No | Yes |
| ENSMUSG00000046318 | Ccbe1 | -0.9525 | 0.001185 | -0.8235 | 0.0005834 | No | Yes |
| ENSMUSG00000046338 | Gpat2 | 1.046 | 0.03687 | 1.083 | 0.01646 | No | Yes |
| ENSMUSG00000046380 | Jrk | -0.9557 | 0.002828 | -1.152 | 1.827e-07 | No | Yes |
| ENSMUSG00000046387 | Pcdhb17 | 0.4227 | 0.01068 | 0.7331 | 5.206e-09 | No | Yes |
| ENSMUSG00000046404 | Yod1 | 0.8556 | 0.0006915 | 1.0243 | 8.766e-09 | No | Yes |
| ENSMUSG00000046417 | Lrrc75a | -0.4113 | 0.04413 | -0.5567 | 0.0007106 | No | Yes |
| ENSMUSG00000046561 | Arsj | -1.543 | 0.000115 | -1.632 | 2.143e-05 | No | Yes |
| ENSMUSG00000046846 | Spesp1 | 4.304 | 0.02364 | 4.2794 | 0.02161 | No | Yes |
| ENSMUSG00000046971 | Pla2g4f | -0.5743 | 0.04358 | -0.7068 | 0.002891 | No | Yes |
| ENSMUSG00000047021 | Cfap65 | 2.5178 | 0.006354 | 3.262 | 0.005989 | No | Yes |
| ENSMUSG00000047036 | Zfp445 | 0.4683 | 0.0004209 | 0.2894 | 0.0003789 | No | Yes |
| ENSMUSG00000047257 | Prss45 | 3.2373 | 0.01375 | 5.2339 | 0.001527 | No | Yes |
| ENSMUSG00000047264 | Zfp358 | -0.7706 | 0.04251 | -0.5976 | 0.02166 | No | Yes |
| ENSMUSG00000047495 | Dlgap2 | -1.432 | 2.747e-05 | -2.115 | 1.838e-15 | No | Yes |
| ENSMUSG00000047554 | Tmem41b | 0.2919 | 0.007976 | 0.325 | 8.085e-05 | No | Yes |
| ENSMUSG00000047694 | Yipf6 | 0.5421 | 0.0006533 | 0.5292 | 8.83e-09 | No | Yes |
| ENSMUSG00000047878 | A4galt | -2.107 | 0.03275 | -1.295 | 0.04122 | No | Yes |
| ENSMUSG00000048118 | Arid4a | 0.282 | 0.04247 | 0.3089 | 0.004389 | No | Yes |
| ENSMUSG00000048126 | Col6a3 | -0.9043 | 0.001583 | -1.115 | 4.722e-10 | No | Yes |
| ENSMUSG00000048240 | Gng7 | -0.8879 | 0.00213 | -0.4975 | 0.00245 | No | Yes |
| ENSMUSG00000048271 | Rbm33 | 0.3655 | 0.01958 | 0.4347 | 1.217e-05 | No | Yes |
| ENSMUSG00000048495 | Tyw5 | 0.5708 | 0.006829 | 0.4015 | 0.002696 | No | Yes |
| ENSMUSG00000048655 | Ccdc169 | 0.913 | 0.00989 | 1.1652 | 0.0001256 | No | Yes |
| ENSMUSG00000048794 | Cfap100 | 0.9329 | 0.02442 | 1.7417 | 0.0002369 | No | Yes |
| ENSMUSG00000048796 | Cyb561d1 | 0.3494 | 0.009285 | 0.3492 | 0.0007599 | No | Yes |
| ENSMUSG00000049184 | Purg | 0.5393 | 0.01153 | 0.3742 | 0.03016 | No | Yes |
| ENSMUSG00000049241 | Hcar1 | -1.127 | 0.03928 | -0.6742 | 0.02487 | No | Yes |
| ENSMUSG00000049285 | Mblac1 | -1.115 | 0.006879 | -1.115 | 0.02418 | No | Yes |
| ENSMUSG00000049409 | Prokr1 | -0.5098 | 0.02004 | -0.8707 | 1.251e-06 | No | Yes |
| ENSMUSG00000049421 | Zfp260 | 0.3683 | 0.005968 | 0.4802 | 3.456e-05 | No | Yes |
| ENSMUSG00000049580 | Tsku | -0.6747 | 0.003028 | -0.4407 | 0.007328 | No | Yes |
| ENSMUSG00000049606 | Zfp644 | 0.4303 | 0.0004957 | 0.2991 | 0.000629 | No | Yes |
| ENSMUSG00000050212 | Eva1b | -0.8143 | 0.02576 | -1.003 | 0.0006847 | No | Yes |
| ENSMUSG00000050335 | Lgals3 | -0.7902 | 8.44e-10 | -0.9221 | 2.1010000000000002e-21 | No | Yes |
| ENSMUSG00000050578 | Mmp13 | 4.9815 | 0.000889 | 2.9015 | 0.009224 | No | Yes |
| ENSMUSG00000050668 | Gpatch11 | 0.4118 | 0.002442 | 0.3077 | 0.00544 | No | Yes |
| ENSMUSG00000050677 | Ccdc96 | -0.8684 | 0.008548 | -0.872 | 0.0007586 | No | Yes |
| ENSMUSG00000050700 | Emilin3 | 1.4248 | 0.009334 | 2.3093 | 0.000648 | No | Yes |
| ENSMUSG00000050947 | Amigo1 | -0.648 | 0.00208 | -0.3283 | 0.01966 | No | Yes |
| ENSMUSG00000051007 | Gatd1 | -0.5817 | 0.003325 | -0.482 | 0.005778 | No | Yes |
| ENSMUSG00000051029 | Serpinb1b | -0.7205 | 0.03281 | -0.7099 | 0.03678 | No | Yes |
| ENSMUSG00000051076 | Vtcn1 | -1.506 | 0.000104 | -2.07 | 1.398e-10 | No | Yes |
| ENSMUSG00000051166 | Eml5 | 0.7947 | 0.01663 | 0.6735 | 0.003999 | No | Yes |
| ENSMUSG00000051285 | Pcmtd1 | 0.2919 | 0.01224 | 0.344 | 9.286e-05 | No | Yes |
| ENSMUSG00000051316 | Taf7 | 0.3245 | 0.03604 | 0.6278 | 2.291e-07 | No | Yes |
| ENSMUSG00000051439 | Cd14 | -0.7698 | 0.002333 | -0.5881 | 0.002844 | No | Yes |
| ENSMUSG00000051592 | Ccnb3 | 4.2926 | 0.02676 | 4.2705 | 0.02339 | No | Yes |
| ENSMUSG00000051674 | Dcun1d4 | 0.4061 | 0.01603 | 0.5986 | 2.196e-06 | No | Yes |
| ENSMUSG00000051703 | Tmem198 | 0.7085 | 0.01224 | 0.9599 | 0.001031 | No | Yes |
| ENSMUSG00000052151 | Plpp2 | -0.8048 | 3.971e-09 | -0.8493 | 4.005e-15 | No | Yes |
| ENSMUSG00000052293 | Taf9 | 1.9905 | 0.01993 | 1.6047 | 0.04904 | No | Yes |
| ENSMUSG00000052395 | Rft1 | -0.471 | 0.009297 | -0.5048 | 1.248e-05 | No | Yes |
| ENSMUSG00000052572 | Dlg2 | 1.6899 | 0.01612 | 2.3493 | 0.0006791 | No | Yes |
| ENSMUSG00000052593 | Adam17 | -0.2794 | 0.01424 | -0.3636 | 6.009e-05 | No | Yes |
| ENSMUSG00000052819 | Best2 | 1.5579 | 0.0002318 | 1.6631 | 0.002784 | No | Yes |
| ENSMUSG00000052921 | Arhgef15 | -0.9093 | 0.002575 | -1.508 | 6.359e-08 | No | Yes |
| ENSMUSG00000053024 | Cntn2 | 2.9462 | 0.01497 | 2.8949 | 0.01258 | No | Yes |
| ENSMUSG00000053297 | AI854703 | 1.8339 | 0.0002354 | 2.5828 | 5.052e-07 | No | Yes |
| ENSMUSG00000053461 | Hhipl2 | 1.2083 | 0.0002915 | 1.5129 | 6.385e-06 | No | Yes |
| ENSMUSG00000053469 | Tg | -0.8079 | 2.441e-09 | -0.6255 | 4.728e-09 | No | Yes |
| ENSMUSG00000053693 | Mast1 | 0.6479 | 0.005506 | 0.7987 | 0.005548 | No | Yes |
| ENSMUSG00000053746 | Ptrh1 | -0.5777 | 0.04487 | -0.9586 | 1.221e-06 | No | Yes |
| ENSMUSG00000053774 | Ubxn7 | 0.272 | 0.01273 | 0.2929 | 0.0002843 | No | Yes |
| ENSMUSG00000054072 | Iigp1 | -1.387 | 4.035e-21 | -1.082 | 1.733e-10 | No | Yes |
| ENSMUSG00000054074 | Skida1 | -0.5614 | 0.04196 | -0.4836 | 0.03396 | No | Yes |
| ENSMUSG00000054387 | Mdm4 | 0.4546 | 0.008549 | 0.2859 | 0.002112 | No | Yes |
| ENSMUSG00000054446 | Cpa1 | -1.726 | 0.02804 | -2.28 | 0.01406 | No | Yes |
| ENSMUSG00000054612 | Mgmt | -0.4702 | 0.0196 | -0.3262 | 0.03605 | No | Yes |
| ENSMUSG00000054793 | Cadm4 | -1.122 | 1.252e-05 | -0.7124 | 4.992e-08 | No | Yes |
| ENSMUSG00000055053 | Nfic | -0.4006 | 0.03264 | -0.6073 | 1.133e-06 | No | Yes |
| ENSMUSG00000055065 | Ddx17 | 0.3859 | 0.002231 | 0.3629 | 0.0001126 | No | Yes |
| ENSMUSG00000055102 | Zfp819 | 2.2536 | 0.02925 | 3.0055 | 0.006827 | No | Yes |
| ENSMUSG00000055240 | Zfp101 | 0.5824 | 1.144e-05 | 0.4896 | 3.717e-05 | No | Yes |
| ENSMUSG00000055333 | Fat2 | -1.234 | 0.01639 | -1.778 | 2.52e-05 | No | Yes |
| ENSMUSG00000055447 | Cd47 | -0.3739 | 0.01879 | -0.4035 | 0.0005632 | No | Yes |
| ENSMUSG00000055632 | Hmcn2 | -0.4128 | 0.03137 | -0.4266 | 0.002897 | No | Yes |
| ENSMUSG00000055723 | Rras2 | -0.7522 | 0.02302 | -0.6341 | 0.003823 | No | Yes |
| ENSMUSG00000056268 | Dennd1b | 0.2964 | 0.009916 | 0.4705 | 5.234e-08 | No | Yes |
| ENSMUSG00000056498 | Tmem154 | -0.4025 | 0.02119 | -0.4503 | 0.003426 | No | Yes |
| ENSMUSG00000056602 | Fry | -0.9903 | 0.01905 | -0.7325 | 0.03108 | No | Yes |
| ENSMUSG00000056656 | Apol8 | -1.461 | 0.001073 | -0.9472 | 0.02036 | No | Yes |
| ENSMUSG00000056671 | Prelid2 | -0.4612 | 0.02234 | -0.7312 | 6.327e-06 | No | Yes |
| ENSMUSG00000056737 | Capg | -0.734 | 0.00335 | -0.8123 | 2.388e-08 | No | Yes |
| ENSMUSG00000057551 | Zfp317 | 0.3487 | 0.008268 | 0.2943 | 0.001052 | No | Yes |
| ENSMUSG00000057894 | Zfp329 | 0.718 | 0.001378 | 0.456 | 0.0003274 | No | Yes |
| ENSMUSG00000058192 | Zfp846 | 0.4127 | 0.003299 | 0.2728 | 0.005199 | No | Yes |
| ENSMUSG00000058396 | Gpr182 | 0.9023 | 0.002057 | 1.5091 | 1.826e-06 | No | Yes |
| ENSMUSG00000058498 | Rnf207 | 1.3242 | 0.008533 | 1.1666 | 0.0129 | No | Yes |
| ENSMUSG00000058550 | Dppa4 | 0.6824 | 4.447e-05 | 0.6246 | 4.089e-09 | No | Yes |
| ENSMUSG00000058624 | Gda | -1.707 | 1.043e-07 | -1.169 | 1.281e-07 | No | Yes |
| ENSMUSG00000058626 | Capn11 | 1.0369 | 0.03689 | 2.2306 | 1.041e-06 | No | Yes |
| ENSMUSG00000059040 | Eno1b | -0.6055 | 5.307e-05 | -0.5147 | 9.694e-07 | No | Yes |
| ENSMUSG00000059213 | Ddn | 2.2246 | 0.003529 | 2.1514 | 0.003948 | No | Yes |
| ENSMUSG00000059920 | 4930453N24Rik | 0.4234 | 0.001119 | 0.2735 | 0.002766 | No | Yes |
| ENSMUSG00000060600 | Eno3 | -0.6808 | 0.0003971 | -0.8189 | 1.433e-14 | No | Yes |
| ENSMUSG00000061531 | Tmem236 | 1.5083 | 0.0007123 | 2.2554 | 7.596e-07 | No | Yes |
| ENSMUSG00000061666 | Gdpd1 | -0.4662 | 0.005252 | -0.4039 | 0.001502 | No | Yes |
| ENSMUSG00000062252 | Lhfpl5 | 1.7483 | 0.009863 | 1.3605 | 0.04487 | No | Yes |
| ENSMUSG00000062480 | Acat3 | 1.4846 | 0.04674 | 1.7477 | 0.004538 | No | Yes |
| ENSMUSG00000062515 | Fabp4 | -1.107 | 0.001976 | -1.564 | 9.797000000000001e-20 | No | Yes |
| ENSMUSG00000062627 | Mysm1 | 0.3215 | 0.008654 | 0.3503 | 5.772e-05 | No | Yes |
| ENSMUSG00000062785 | Kcnc3 | 1.3675 | 0.04587 | 3.5703 | 0.0008967 | No | Yes |
| ENSMUSG00000062980 | Cped1 | -0.747 | 0.01186 | -0.4418 | 8.674e-05 | No | Yes |
| ENSMUSG00000063281 | Zfp35 | 0.3181 | 0.02055 | 0.4275 | 1.988e-05 | No | Yes |
| ENSMUSG00000063409 | Lrrc43 | 1.9231 | 0.005027 | 1.4036 | 0.02467 | No | Yes |
| ENSMUSG00000063524 | Eno1 | -0.5746 | 0.0001019 | -0.471 | 1.969e-07 | No | Yes |
| ENSMUSG00000063663 | Brwd3 | 0.3355 | 0.01341 | 0.5638 | 1.586e-08 | No | Yes |
| ENSMUSG00000063687 | Pcdhb5 | 0.458 | 0.01435 | 0.5392 | 0.001236 | No | Yes |
| ENSMUSG00000064128 | Cenpj | 0.3709 | 0.01146 | 0.4978 | 7.594e-06 | No | Yes |
| ENSMUSG00000064254 | Ethe1 | -0.4525 | 0.03379 | -0.3389 | 0.006918 | No | Yes |
| ENSMUSG00000064330 | Pde6h | -0.7936 | 0.0402 | -0.9654 | 0.002401 | No | Yes |
| ENSMUSG00000065954 | Tacc1 | -0.4503 | 0.004288 | -0.264 | 0.02854 | No | Yes |
| ENSMUSG00000066026 | Dhrs3 | -1.256 | 0.0004647 | -0.9331 | 8.787e-07 | No | Yes |
| ENSMUSG00000066152 | Slc31a2 | -0.5337 | 0.005273 | -0.6004 | 1.713e-05 | No | Yes |
| ENSMUSG00000066382 | Iqcf5 | -1.274 | 0.02525 | -1.254 | 0.0268 | No | Yes |
| ENSMUSG00000067369 | Trmt2b | 0.3348 | 0.03868 | 0.3105 | 0.007579 | No | Yes |
| ENSMUSG00000067597 | Dgat2l6 | 3.9271 | 4.877e-05 | 5.9263 | 3.229e-06 | No | Yes |
| ENSMUSG00000067889 | Sptbn2 | 0.5319 | 0.04107 | 0.6309 | 0.0005063 | No | Yes |
| ENSMUSG00000068259 | Olfr461 | 1.5943 | 0.005937 | 2.4733 | 1.746e-05 | No | Yes |
| ENSMUSG00000068606 | Gm4841 | -2.406 | 3.099e-05 | -1.572 | 0.01445 | No | Yes |
| ENSMUSG00000068697 | Myoz1 | -0.8222 | 0.03634 | -0.8782 | 0.0168 | No | Yes |
| ENSMUSG00000068735 | Trp53i11 | -0.5482 | 0.02186 | -0.3622 | 0.0408 | No | Yes |
| ENSMUSG00000069189 | Zdhhc11 | 2.6057 | 0.001231 | 3.0612 | 0.0002486 | No | Yes |
| ENSMUSG00000069237 | Fam8a1 | 0.2788 | 0.01118 | 0.4442 | 1.361e-06 | No | Yes |
| ENSMUSG00000069808 | Tlcd3a | -0.459 | 0.01061 | -0.2804 | 0.02785 | No | Yes |
| ENSMUSG00000069920 | B3gnt9 | -0.6657 | 0.00444 | -0.5878 | 0.001564 | No | Yes |
| ENSMUSG00000070003 | Ssbp4 | -0.547 | 0.001224 | -0.5037 | 1.335e-05 | No | Yes |
| ENSMUSG00000070385 | Ampd1 | 1.8066 | 0.00072 | 1.9777 | 2.818e-07 | No | Yes |
| ENSMUSG00000070643 | Sox13 | -1.621 | 0.0004028 | -1.434 | 0.001286 | No | Yes |
| ENSMUSG00000070705 | Eid2b | -0.4232 | 0.02198 | -0.4458 | 0.005344 | No | Yes |
| ENSMUSG00000071176 | Arhgef10 | -0.463 | 0.003417 | -0.5593 | 4.102e-07 | No | Yes |
| ENSMUSG00000071262 | Zfp957 | 2.4105 | 0.03237 | 3.1647 | 0.01159 | No | Yes |
| ENSMUSG00000071533 | Pcnp | 0.2695 | 0.01261 | 0.2893 | 0.0003513 | No | Yes |
| ENSMUSG00000071658 | Gng3 | 1.183 | 0.02281 | 1.4044 | 1.962e-05 | No | Yes |
| ENSMUSG00000071660 | Ttc9c | 0.3308 | 0.01096 | 0.3597 | 0.0001698 | No | Yes |
| ENSMUSG00000072494 | Ppp1r3e | -0.6405 | 0.03549 | -1.061 | 2.6e-05 | No | Yes |
| ENSMUSG00000072572 | Slc39a2 | 0.5291 | 0.02514 | 0.4293 | 0.0003402 | No | Yes |
| ENSMUSG00000072620 | Slfn2 | -1.281 | 2.533e-16 | -1.021 | 4.558e-10 | No | Yes |
| ENSMUSG00000072919 | Noxred1 | 1.6593 | 8.622e-06 | 2.333 | 3.523e-10 | No | Yes |
| ENSMUSG00000073639 | Rab18 | 0.2651 | 0.04373 | 0.2737 | 0.002131 | No | Yes |
| ENSMUSG00000074001 | Klhl40 | 2.8146 | 0.01682 | 2.78 | 0.02166 | No | Yes |
| ENSMUSG00000074158 | Zfp976 | 0.6094 | 0.0001036 | 0.3571 | 0.002913 | No | Yes |
| ENSMUSG00000074264 | Amy1 | 1.7062 | 0.0004677 | 2.0848 | 7.721e-05 | No | Yes |
| ENSMUSG00000074665 | Bpifb4 | -1.514 | 0.00306 | -1.189 | 0.01837 | No | Yes |
| ENSMUSG00000075569 | Rsph10b | 1.5693 | 0.000423 | 1.2838 | 0.001059 | No | Yes |
| ENSMUSG00000075706 | Gpx4 | -0.512 | 0.0005888 | -0.5783 | 2.914e-11 | No | Yes |
| ENSMUSG00000078532 | Nkain1 | -0.6011 | 0.004837 | -0.9589 | 1.987e-12 | No | Yes |
| ENSMUSG00000078671 | Chd2 | 0.3085 | 0.04707 | 0.3541 | 0.001572 | No | Yes |
| ENSMUSG00000078851 | H2aw | 0.7849 | 0.0122 | 0.8195 | 0.02179 | No | Yes |
| ENSMUSG00000078872 | Gm14401 | 0.7347 | 0.004719 | 0.563 | 0.04428 | No | Yes |
| ENSMUSG00000078902 | Gm14443 | 0.7492 | 0.001934 | 0.5451 | 3.298e-05 | No | Yes |
| ENSMUSG00000079304 | Tex52 | 1.2319 | 0.006232 | 0.7195 | 0.04706 | No | Yes |
| ENSMUSG00000079418 | Atg4a | 0.3765 | 0.01881 | 0.5329 | 1.016e-07 | No | Yes |
| ENSMUSG00000079426 | Arpc4 | -0.3689 | 0.008575 | -0.488 | 2.078e-06 | No | Yes |
| ENSMUSG00000079550 | Mpp4 | 1.7879 | 0.009537 | 2.4361 | 0.0004217 | No | Yes |
| ENSMUSG00000084128 | Esrp2 | 1.6061 | 0.01746 | 2.481 | 0.001488 | No | Yes |
| ENSMUSG00000086228 | Ubap1l | 2.9816 | 5.504e-05 | 1.9925 | 0.001284 | No | Yes |
| ENSMUSG00000086316 | Nbdy | 0.4177 | 0.02558 | 0.7152 | 1.609e-07 | No | Yes |
| ENSMUSG00000087119 | Atg4a-ps | 0.565 | 0.03324 | 0.5647 | 0.03094 | No | Yes |
| ENSMUSG00000087403 | Kantr | 0.4114 | 0.01739 | 0.5632 | 9.445e-06 | No | Yes |
| ENSMUSG00000087679 | Tmem250-ps | -0.347 | 0.005903 | -0.4023 | 4.194e-06 | No | Yes |
| ENSMUSG00000089857 | Zfp882 | 0.3674 | 0.01152 | 0.3585 | 0.001264 | No | Yes |
| ENSMUSG00000089876 | Tmem102 | -0.8704 | 0.02551 | -0.9193 | 0.002613 | No | Yes |
| ENSMUSG00000089997 | 1810020O05Rik | 1.4473 | 0.02428 | 2.3189 | 0.0007389 | No | Yes |
| ENSMUSG00000090100 | Ttbk2 | 0.2645 | 0.03296 | 0.3281 | 0.0008125 | No | Yes |
| ENSMUSG00000090110 | Cmc4 | 0.6485 | 0.005369 | 0.5574 | 0.002233 | No | Yes |
| ENSMUSG00000090206 | Tepp | 4.8793 | 0.00256 | 4.8513 | 0.002106 | No | Yes |
| ENSMUSG00000090236 | Car15 | 0.7994 | 0.03354 | 1.5321 | 3.857e-05 | No | Yes |
| ENSMUSG00000090272 | Mndal | -1.656 | 0.01273 | -0.5803 | 0.03372 | No | Yes |
| ENSMUSG00000090439 | Gm17455 | -1.434 | 0.000485 | -1.484 | 0.0002986 | No | Yes |
| ENSMUSG00000090523 | Gypc | -0.623 | 0.007071 | -0.5961 | 1.091e-08 | No | Yes |
| ENSMUSG00000090874 | Olfr733 | -1.076 | 0.0341 | -1.386 | 0.003942 | No | Yes |
| ENSMUSG00000091504 | Vmn2r118 | 5.5813 | 3.543e-05 | 2.6809 | 0.005519 | No | Yes |
| ENSMUSG00000091679 | Vmn2r96 | -1.175 | 1.706e-05 | -0.706 | 0.004308 | No | Yes |
| ENSMUSG00000091735 | Gpr62 | 1.4974 | 0.01728 | 2.5694 | 1.183e-05 | No | Yes |
| ENSMUSG00000091844 | Gm8251 | 2.3153 | 7.383e-06 | 1.9301 | 5.203e-05 | No | Yes |
| ENSMUSG00000094410 | Zbed6 | 0.4057 | 0.0002621 | 0.4123 | 4.176e-05 | No | Yes |
| ENSMUSG00000095377 | Olfr91 | 1.2179 | 0.01875 | 1.8738 | 0.000697 | No | Yes |
| ENSMUSG00000095620 | Csta2 | 0.8467 | 0.03559 | 1.0712 | 0.001649 | No | Yes |
| ENSMUSG00000096035 | Odaph | -1.696 | 0.002105 | -1.579 | 0.01563 | No | Yes |
| ENSMUSG00000096188 | Cmtm4 | -0.3806 | 0.04059 | -0.3147 | 0.001207 | No | Yes |
| ENSMUSG00000096210 | H1f0 | -0.3235 | 0.01941 | -0.3532 | 0.001672 | No | Yes |
| ENSMUSG00000096351 | Samd11 | 1.1079 | 0.01652 | 1.0242 | 0.001931 | No | Yes |
| ENSMUSG00000096798 | Gm6309 | 4.8673 | 0.001705 | 4.8414 | 0.00294 | No | Yes |
| ENSMUSG00000096910 | Zfp955b | 0.3619 | 0.002036 | 0.5066 | 1.624e-09 | No | Yes |
| ENSMUSG00000097392 | Thoc2l | 0.6297 | 0.0004001 | 0.6109 | 4.776e-11 | No | Yes |
| ENSMUSG00000097789 | Gm2115 | -0.6781 | 0.03393 | -0.7989 | 0.003637 | No | Yes |
| ENSMUSG00000098134 | Rnf113a2 | 0.3707 | 0.02655 | 0.2952 | 0.02233 | No | Yes |
| ENSMUSG00000100079 | Ifnab | 3.0619 | 0.006081 | 5.8576 | 0.000115 | No | Yes |
| ENSMUSG00000102976 | Zc3h11a | 0.4559 | 0.0006108 | 0.2938 | 0.004659 | No | Yes |
| ENSMUSG00000104158 | Becn2 | 2.8035 | 0.0004056 | 6.8213 | 1.807e-08 | No | Yes |
| ENSMUSG00000104301 | Wdr49 | 0.8641 | 7.78e-06 | 0.7982 | 5.374e-09 | No | Yes |
| ENSMUSG00000106350 | Kif19b | 2.3091 | 0.002118 | 6.0334 | 6.931e-07 | No | Yes |
| ENSMUSG00000111375 | Btbd8 | -0.4903 | 0.002711 | -0.5263 | 0.0001434 | No | Yes |
| ENSMUSG00000111497 | Gm38431 | 0.9388 | 0.04889 | 2.1228 | 0.0001013 | No | Yes |
| ENSMUSG00000112160 | BC024063 | 0.4541 | 0.008256 | 0.4458 | 0.0009285 | No | Yes |
| ENSMUSG00000116121 | Gm49486 | 1.0421 | 0.02079 | 1.3148 | 0.0008714 | No | Yes |
| ENSMUSG00000117679 | Apbb3 | 0.4016 | 0.04649 | 0.4163 | 0.01002 | No | Yes |
| ENSMUSG00000118125 | Gm50387 | 0.5948 | 0.0426 | 0.556 | 0.03307 | No | Yes |
| ENSMUSG00000118506 | Cfap141 | 0.8109 | 0.01991 | 1.1056 | 2.258e-07 | No | Yes |
| ENSMUSG00000118537 | Shld3 | 0.5067 | 0.03299 | 0.6482 | 0.002235 | No | Yes |
| ENSMUSG00000118667 | Ahnak2 | -0.8052 | 7.713e-06 | -0.7846 | 3.286e-09 | No | Yes |
| ENSMUSG00000118668 | Rps6ka4 | -0.3533 | 0.02193 | -0.3816 | 0.0007256 | No | Yes |
| ENSMUSG00000118671 | Eppk1 | -1.019 | 0.04076 | -1.001 | 0.008573 | No | Yes |
